# Measuring digital stress in Norway: translation and validation of the Digital Stressors Scale

**DOI:** 10.3389/fpsyg.2024.1297194

**Published:** 2024-02-09

**Authors:** Aleksandra Sevic, Njål Foldnes, Kolbjørn Kallesten Brønnick

**Affiliations:** ^1^Department of Public Health, Faculty of Health Sciences, University of Stavanger, Stavanger, Norway; ^2^Cognitive and Behavioral Neuroscience Lab, University of Stavanger, Stavanger, Norway; ^3^SHARE-Centre for Resilience in Healthcare, Faculty of Health Sciences, University of Stavanger, Stavanger, Norway; ^4^Norwegian Centre for Reading Education and Research, University of Stavanger, Stavanger, Norway; ^5^Department of Social Studies, Faculty of Social Sciences, University of Stavanger, Stavanger, Norway

**Keywords:** technostress, digital stress, psychometric testing, translation, validity, reliability, confirmatory factor analysis, employees

## Abstract

**Introduction:**

A better understanding of the effects of the widespread use of information and communication technology (ICT) among employees is important for maintaining their wellbeing, work-life balance, health, and productivity. Thus, having robust and reliable measurement instruments is crucial for quantifying the effects of ICT use, and facilitating the development of effective strategies to promote employee wellbeing.

**Methods:**

Therefore, we translated the Digital Stressors Scale (DSS) to Norwegian and administered it to a convenience sample of 1,228 employees, using the forward-backward translation method. The DSS is a new multidimensional scale consisting of 50 items that measure 10 digital stressors (first-order factors), and a second-order factor of DSS. We assessed the scale's construct validity with confirmatory factor analysis, first by assessing the model fit of each of the sub-scales separately, to facilitate the disaggregated measurement approach, and then the model fit of the whole scale with the second-order factor.

**Results:**

Among the participants, 45.6% completed the whole questionnaire (*n* = 560). The original solution's fit was unsatisfactory in our sample, which led us to perform an exploratory factor analysis. We propose a shorter 8-factor scale with 37 of the original items, which also shows good internal consistency for all the first-order factors.

**Discussion:**

We argue that the disaggregated approach is beneficial for the investigation of the specific creators of digital stress and that conceptually sound measurement models are needed in order to facilitate a more rigorous empirical investigation of digital stressors.

## 1 Introduction

The widespread adoption of information and communication technology (ICT) in today's workplaces has introduced a new issue: digital stress, also known as technostress^1^. While the use of ICT offers numerous advantages such as improved efficiency, connectivity, and productivity, there is a rising concern about the stress that workers face as a result of excessive use or reliance on technology. This may result in adverse consequences for employee health and organisational performance. Empirical evidence on the topic, has found that the consequences of digital stress are far-reaching, and include psychological outcomes such as exhaustion, anxiety, burnout, role conflict, as well as behavioral outcomes such as decreased productivity, task performance, employee innovation and so on (Ragu-Nathan et al., 2008; Tarafdar et al., 2011; Salanova et al., 2013; Fischer et al., 2019, 2021; Nastjuk et al., 2023). The effects of digital stress extend beyond individual consequences and also impact organizations, leading to heightened absenteeism, reduced productivity, decreased organizational commitment, and increased employee turnover rates (Boyer-Davis, 2019). In recent years, there has been a notable increase in research attention toward this field, highlighting the considerable negative impact of digital stress that diminishes the benefits of ICT.

Some of the earliest attempts to measure stress related to technology include The Computer Anxiety Scale (Heinssen Jr et al., 1987) and The Computer Hassles Scale (Hudiburg, 1989). One of the more established, widely recognised and commonly used tools for assessing technostress creators in organisations - The Technostress Creators Inventory was developed by Ragu-Nathan et al. (2008). The scale describes five different technostress creators: Techno-overload, Techno-complexity, Techno-invasion, Techno-uncertainty and Techno-insecurity, and has been widely used in scientific research on digital stress (Fuglseth and Sørebø, 2014; Molino et al., 2020; Zhao et al., 2020; Torres, 2021). Technostress creators or stressors are defined as perceived demands, conditions or circumstances related to the use of ICT that may lead to users experiencing technostress (Fuglseth and Sørebø, 2014). This may manifest as a persistent stream of emails and notifications, frequent distractions, the expectation of being constantly available and accessible, or a demand to stay on top of rapidly changing technology.

In their 2019 article Fischer et al. (2019) argued that the traditional five stressor categories no longer capture the entirety of the digital stress phenomenon. They highlighted the necessity for further exploration into the dimensions of digital stressors, due to the rapid changes in today's technological landscape. Additionally, the authors emphasised the importance of regularly updating the instruments that measure digital stress(ors), in order for them to be able to capture the advancements in technology, patterns of use, and emerging stressors, thereby supporting the content validity of the instrument in question. More recently, Fischer et al. (2021) introduced a novel tool for measuring the perception of digital stressors in the workplace context, known as The Digital Stressors Scale (DSS). With the DSS, authors Fischer et al. (2021) sought to provide an update to the Technostress Creators Inventory (Ragu-Nathan et al., 2008). The newly proposed instrument uses reflective models to measure 10 different digital stressors, conceptualised as 1st order factors, as well as a 2nd order factor of perceived digital stress, which the authors refer to as the DSS.

Since technostress (digital stress) field is still fairly young, having psychometrically sound instruments is of crucial importance for advancing the knowledge base, ensuring the integrity of research, refining the existing hypotheses, and thus further nuancing the understanding of the digital stress phenomenon. In this regard, the process of instrument validation is seen as a continuous one, particularly so for newly established instruments that have yet to undergo thorough empirical investigation (Edwards, 2003; Flora and Flake, 2017; Dima, 2018).

Evaluating construct validity is particularly important in fields such as psychology and social sciences, where studied phenomena cannot be measured directly. Construct validity, thus, provides important insight into whether an instrument actually measures what it was intended to measure. Loevinger (1957) and Nunnally (1978) [as cited in Benson (1998)] described construct validation in three distinctive phases (components): a substantive phase, a structural phase, and an external phase. The substantive phase focuses on constructing a clear definition of the construct within its theoretical domain and identifying the different aspects of the construct through the development of scale items. The structural phase is concerned with examining the relationship between items and the structure of the construct, assessing the extent to which the items are related to each other and to the construct itself (our study is positioned in this phase). Finally, the external phase is focused on determining whether the measures of a given construct are related to the measures of other constructs in an expected manner (Benson, 1998). The external phase aligns with Campbell's concept of nomological validity, i.e., the degree to which the construct measured by the instrument is related to other concepts in a broader theoretical perspective (Campbell, 1960; Edwards, 2003). It is recommended to thoroughly assess aspects of construct validity, such as item-construct relationships and reliability, before delving into the assessment of nomological validity in a broader theoretical framework (Edwards, 2003). The three phases of construct validation are considered complementary and essential in the continuous process of validating a measurement instrument. In this process, confirmatory factor analysis (CFA) is commonly employed in the structural phase, for psychometric evaluation, construct evaluation, measurement invariance testing etc. (Hoyle, 2023).

In their article Fischer et al. (2021) report the development and validation process of the Digital Stressors Scale through the aforementioned construct validation process using a sample from the United States (US). The psychometric properties of the DSS scale in the original article Fischer et al. (2021) were evaluated using partial least squares path modelling (PLS-SEM, Hair et al., 2019). PLS - SEM has been extensively adopted, and vigorously debated by researchers across various disciplines (Rouse and Corbitt, 2008), ranging from marketing (Guenther et al., 2023), quality management (Magno et al., 2022), management information systems (Marcoulides and Saunders, 2006; Goodhue et al., 2012), strategic management (Sarstedt et al., 2014) and so on. According to a recent review of 139 articles that applied PLS-SEM by Zeng et al. (2021), the most common reasons for employing PLS-SEM were small sample size, non-normal data, formative measures and model complexity. The study also highlighted that PLS-SEM is often used when the theoretical measures are not well formed and studies exploratory in nature. Nonetheless, the strengths of PLS-SEM have been challenged by researchers pointing out the shortcomings of this methodological approach compared to covariance based SEM, urging the researchers to use PLS-SEM with caution (Goodhue et al., 2012). Given its limitations when modelling latent variables and conducting formal model fit tests, but also the reflective nature of the DSS model, we chose to perform covariance-based modelling in the present study, which is traditionally used for confirmatory purposes (Rönkkö et al., 2016, 2023).

Moreover, as this is a novel measurement, continuing the validation process in other contexts is necessary. The cross-cultural translation and validation of instruments plays a vital role by providing researchers with reliable tools in different languages, particularly for research topics that have wide-ranging relevance. Considering that digital stressors might differ across countries and organisational contexts, it is important to have robust tools to explore the subtleties of the digital stress phenomenon outside of the US. These variations may arise due to various factors, such as differences in the perception of work-life balance across different countries, the degree of acceptance of technology's widespread use in society, or the modes of communication prevalent in organisational settings. According to Fischer et al. (2021), their research offers initial support for the DSS as an internationally relevant tool for measuring technostress across diverse cultures. Still authors described the somewhat extensive and challenging process of item and factor reduction and called for further investigation of the factor structure of the Digital Stressors Scale.

Against this backdrop, we translated and validated the Digital Stressors Scale (DSS) for the use among Norwegian employees. Validating the translated DSS among Norwegian employees is vital for several reasons. First, it guarantees that the scale effectively measures digital stressors in the workplace within the cultural and linguistic context of Norway. Moreover, validated instruments enable the expansion of existing knowledge and theories. Given the wide-range of adverse effects associated with digital stress among employees, having robust and up-to date instruments for measuring various digital stressors is of both theoretical and practical importance. We aimed to address this by further supporting the development and validation efforts for a new scale by Fischer et al. (2021), the Digital Stressors Scale. By performing a validation of the DSS, our aim is to contribute to a broader body of research on digital stress, and to facilitate the ongoing efforts in the field. Furthermore, adopting an up to date instrument in Norway is particularly important due to the limited research on digital stress in the country so far. This will hopefully, allow us to address the significant gap in the current knowledge base regarding digital stress in Norway. To the best of our knowledge this is the first translation and validation study of the DSS outside the US context.

In light of the above mentioned considerations, the aims of this study were to translate and examine the psychometric properties of the Digital Stressors Scale by examining the factor structure of the DSS in a Norwegian sample, as well as internal consistency of the translated instrument.

## 2 Materials and methods

### 2.1 Instrument

The Digital Stressors Scale (DSS, Fischer et al., 2021 is a comprehensive instrument designed to measure ten different creators of digital stress, or 1st order factors. The DSS consists of 50 items, each measured with a 7-point Likert scale that ranges from strongly disagree (1) to strongly agree (7), with each of the first order factors being measured by five items. The 10 digital stressors are further related to the overarching concept of perceived digital stress, which serves as a second order factor. The DSS has been previously validated on a sample of 1998 employees in the United States and has demonstrated good psychometric properties according to Fischer et al. (2021). The ten 1st order factors measured by the DSS are: Complexity (perception of ICT complexity), Conflicts (work-life balance), Insecurity (fear of being replaced by ICTs), Invasion of Privacy (perceived of invasion of privacy facilitated by ICTs), Overload (information/work overload enabled by ICTs), Safety (of use of ICT), Social environment (pressure from the social involvement), Technical support availability, Usefulness (of ICT tools for work tasks) and Unreliability (of ICT solutions).

### 2.2 Participants and procedure

Between July and November 2022, a sample of employees from the general public, was recruited for the study through social media. The recruitment strategy included the dissemination of the study's participation link strategically across different Facebook groups in different regions of Norway. This was done deliberately to include a diverse section of the general population and ensure participants had varied backgrounds. Study participation links were posted in general help groups (“Hjelp til alt/Help for everything”) that can be found in each region of the country; in the groups for different professionals (psychologists, teachers, nurses, etc.) and in the groups for recruitment of research participants. Data was collected using an online survey platform—SurveyExact. The 50 items comprising the DSS were divided across pages in sets of 10 items per page to decrease respondent fatigue. The first page consisted of the items related to the Complexity and Conflicts factors. The second page included items from the Insecurity and Invasion of privacy factors. On the third page, participants found items related to the Overload and Safety factors. While, the fourth and fifth pages contained items related to the Social environment and Technical support, and Usefulness and Unreliability factors, respectively.

### 2.3 Translation process

To adapt the DSS scale to Norwegian, a forward-backward translation process was employed, following the methodology recommended by Brislin (1970). Firstly, a native Norwegian speaker proficient in English (KKB) and familiar with the purpose and content of each item, performed a forward translation of the instrument. Subsequently, an independent translator, a Norwegian and English bilingual (VLHF), performed a backward translation without prior knowledge of the instrument. AS and KKB conducted a thorough review of the backward translation in comparison to the original instrument, and subsequently addressed any identified discrepancies by making requisite adjustments to the Norwegian version with the purpose of enhancing the semantic equivalence of the translated version.

### 2.4 Statistical analysis

Prior to conducting a confirmatory factor analysis (CFA), we ran a series of preliminary analyses. We used the naniar (Tierney and Cook, 2023) package in R software and generated a summary plot to illustrate the extent of missing data. Thereafter we proceeded with a more in-depth analysis of missing data. Previous studies, particularly on health-related surveys (Elliott et al., 2005), have shown that nonrespondents tend to be less healthy compared to respondents. Therefore we explored if nonresponsiveness in our sample was linked to perceived digital stressors. We did this by investigating the number of missing participants across the five pages of the online survey (see details and R code in Supplementary material). To address the possibility of non-random missingness, we assessed whether there were discernible differences in the scores obtained on the first page between individuals who dropped out by page 5 and those who completed the entire questionnaire. Our aim was to examine whether individuals who dropped out had higher scores on the first two digital stressors in comparison to those who completed the whole questionnaire. *t*-test was employed to test for differences. In order to identify potential multivariate outliers, we employed the robust Minimum Covariance Determinant (MCD) method (Hubert and Debruyne, 2010). Additionally, Mardia's method was used for the assessment of multivariate normality (Von Eye and Bogat, 2004). Subsequently, we examined the correlation plot of the fifty scale items. See page 12 in the Supplementary material for details.

Our further analysis consisted of two parts.

#### 2.4.1 Part 1: initial CFA and reliability analysis

Based on the factor structure of the DSS established by Fischer et al. (2021), we ran the CFA using the Norwegian employee sample to confirm whether the same factor structures (10 first order factors and one 2nd order factor) existed in our sample. Given the novelty of the scale and its validation in a single data sample pertaining to a different country than that investigated in the present study, it is plausible that our data would depart from the original structure proposed by Fischer et al. (2021). For the validity of the DSS in the Norwegian context, it was, therefore, important to assess how well the original findings were replicated in the new context.

We evaluated the model fit, first by assessing the measurement quality of each 1st order factors separately, as well as the factor loadings. After having evaluated each of the ten latent constructs individually, we proceeded to estimate and evaluate the fit of the full second-order model proposed by Fischer et al. (2021). Although there are no definitive cut-offs for model fit indices, the extent to which the full model, and each sub-scale, were deemed adequate was given by the following recommendations (Kline, 2016, p. 269): Root mean square error of approximation (RMSEA) fit index (<0.06 is “close fit”, and <0.08 is “acceptable”, >0.08 “poor”), comparative fit index(CFI) (<0.90 is “poor”, >0.90 is “acceptable”, >0.95 is “good”) and the standardized root mean square (SRMR) (<0.08 is “acceptable”, and <0.06 is “good”)^2^.

Given the ordinal-categorical nature of the scale items, we estimated all factor models using the diagonally weighted least squares (DWLS) estimator based on polychoric correlations. However, as recent studies (Foldnes and Grønneberg, 2022; Grønneberg and Foldnes, 2022) have shown, this approach is contingent upon a normality assumption that is hard to verify in practice. We therefore also treated the data as continuous and employed approximately robust test statistics and standard errors (Satorra and Bentler, 1994). (See Supplementary material for more details). Unless noted, the substantive conclusions from analyses using DWLS or robust maximum likelihood (MLR) were aligned, and results attained by MLR are reported. For all CFA analyses, we employed the package lavaan (Rosseel, 2012).

In order to assess the internal consistency reliability of the first-order factors, we calculated Cronbach's α (along with confidence intervals), and McDonald's ω coefficients. Cronbach's α is widely utilized as a measure of internal consistency in psychology. However, α has known limitations and relies on assumptions that are seldom feasible in practice, such as essential tau equivalence. To address these concerns, we also computed McDonald's ω as an alternative measure of internal consistency (McNeish, 2018; Flora, 2020). For Cronbach's α, cutoff values over 0.80, are generally regarded as indicating excellent reliability for research purposes, while cutoff values over 0.90 are considered as excellent when it comes to instruments used for clinical purposes (Henson, 2001). Given the hierarchical nature of the DSS, we calculated also ω hierarchical which is used to assess the reliability of the second-order factor, while taking into consideration the variance of the lower-order factors (Flora, 2020) For reliability analyses, we employed the package semTools (Jorgensen et al., 2022).

#### 2.4.2 Part 2: exploratory factor analysis

Due to the unsatisfactory model fit of the original solution, we performed a subsequent step in our analysis. In this stage, we conducted an EFA on the 50 items of the Digital Stressors Scale, applying the oblique (oblimin) rotation, to find an optimal number of factors reflecting latent structures among Norwegian employees. To assess the suitability of the data for EFA, the Kaiser-Meyer-Olkin (KMO) measure of sampling adequacy and Bartlett's test of sphericity were examined. The KMO measure assesses the proportion of variance among variables that might be caused by latent factors. Values closer to 1 suggest that there are significant relationships among variables, which indicates that the data is suitable for factor analysis, while Bartlett's test of sphericity tests the null hypothesis that the variables are uncorrelated. A significant result (p <0.05) indicates that the variables are not independent and are suitable for factor analysis. We estimated EFA using normal-theory maximum likelihood estimation (ML). To estimate the adequate number of factors, we used scree plot, Keiser's criterion (eigenvalues > 1) and Horn's parallel analysis (PA) (Hoyle, 2023). We employed a standardized factor loading threshold of .55 as a cutoff. This threshold signifies that at least 30% of the item variance stems from the common factor (Tabachnick et al., 2013). Items with factor loadings below the said threshold were removed in a stepwise fashion. Regarding the items with high-cross loadings on two or more items (>0.31), we followed the recommendations of Worthington and Whittaker (2006) and excluded those items from further analysis, as high cross-loadings suggest that they are complex and influenced by more then one factor. The process of item elimination started with the item that had the highest cross-loading and proceeded sequentially to the item that was the least cross-loading. Following the removal of each item, the analysis was repeated and eventually concluded when no items with cross-loadings remained. To assess the reliability of the 8-factor soultion we caluclated Cronbach's alpha and McDonald's omega coefficients. Lastly, to assess discriminant validity we calculated inter-factor correlations and their confidence intervals(CIs) with sem Tools *discriminant Validity* (Jorgensen et al., 2022) function in line with recommendations by Rönkkö and Cho (2022).

All analyses were performed in the R software environment (R Core Team, 2022) using packages lavaan (Rosseel, 2012), psych (Revelle, 2017), GPArotation (Bernaards and Jennrich, 2005), and semTools (Jorgensen et al., 2022).

## 3 Results

### 3.1 Participants and preliminary data analysis

A total of 1,228 participants entered the study, and 45.6 % of these (560) answered all of the items in the questionnaire (completers). All of the participants in the study were employees. Among completers 78% were females; 68% were either married or cohabiting, and the average age was 42 years (SD = 11.2). Additionally, 70% of the participants had higher educational attainment, with 39% having completed up to 4 years of university education, and 31% having completed more than 4 years of university education. Data from these participants were used for the CFAs and the EFA. For more details on demographics, see Supplementary material.

We explored the missingness in our data. The likelihood of missing response increased over the five pages (ten items on each page) that comprised the scale on the SurveyExact platform. To investigate whether missingness in our data was related to the perception of digital stressors we calculated the mean score of the ten items as reported on page 1 in two groups those who dropped out by the last page (page 5, *n* = 188) vs those who had at least one reply on the last page (*n* = 584). The mean scores were 3.52 and 3.35, respectively. However, this difference was not significant according to the t-test (*t* = 1.43, *df* = 283.15, *p*-value = 0.16). We therefore found little support in the data to claim that missingness in our sample was related to the digital stressors. Therefore we removed the participants that had missing responses on the DSS, and continued the analyses on the complete cases (*n* = 560).

We proceeded with calculating the descriptive statistics for each DSS item (see Table 1). The univariate statistics suggested that the majority of variables within the DSS scale resembled an approximately symmetric distribution. Mardia's tests of multivariate normality strongly supported multivariate non-normality. Therefore, robustified estimators were used in our analyses.

**Table 1:** Summary statistics.

**Statistic**	***N***	**Mean**	**St. Dev**.	**Min**	**Pctl(25)**	**Median**	**Pctl(75)**	**Max**
COMP1	560	3.259	1.772	1	2	3	5	7
COMP2	560	3.525	1.959	1	2	3	5	7
COMP3	560	3.341	1.800	1	2	3	5	7
COMP4	560	4.346	1.985	1	2	5	6	7
COMP5	560	3.945	2.056	1	2	4	6	7
CONF1	560	3.096	1.878	1	1	3	5	7
CONF2	560	2.891	1.813	1	1	2	4	7
CONF3	560	3.275	1.929	1	2	3	5	7
CONF4	560	2.857	1.766	1	1	2	4	7
CONF5	560	3.057	1.810	1	2	3	5	7
INSE1	560	2.071	1.423	1	1	2	3	7
INSE2	560	1.852	1.381	1	1	1	2	7
INSE3	560	1.975	1.423	1	1	1	2	7
INSE4	560	1.736	1.349	1	1	1	2	7
INSE5	560	1.736	1.304	1	1	1	2	7
PRIV1	560	3.279	1.749	1	2	3	5	7
PRIV2	560	3.577	1.788	1	2	4	5	7
PRIV3	560	3.807	1.696	1	2	4	5	7
PRIV4	560	3.961	1.666	1	3	4	5	7
PRIV5	560	3.932	1.687	1	3	4	5	7
OVER1	560	3.700	1.893	1	2	4	5	7
OVER2	560	3.841	1.946	1	2	4	5	7
OVER3	560	4.209	1.887	1	2	4	6	7
OVER4	560	3.202	1.729	1	2	3	4	7
OVER5	560	3.954	1.856	1	2	4	5	7
SAFE1	560	2.675	1.548	1	2	2	4	7
SAFE2	560	2.707	1.578	1	2	2	4	7
SAFE3	560	2.861	1.686	1	2	2	4	7
SAFE4	560	3.000	1.750	1	2	3	4	7
SAFE5	560	2.782	1.643	1	2	2	4	7
SOCI1	560	3.082	1.789	1	2	3	4	7
SOCI2	560	4.023	1.942	1	2	4	6	7
SOCI3	560	2.975	1.730	1	2	2	4	7
SOCI4	560	4.068	1.874	1	2	4	6	7
SOCI5	560	3.255	1.732	1	2	3	5	7
TECH1	560	3.011	1.743	1	2	2	4	7
TECH2	560	3.587	1.946	1	2	3	5	7
TECH3	560	3.861	1.991	1	2	4	6	7
TECH4	560	3.848	1.969	1	2	4	6	7
TECH5	560	3.586	1.972	1	2	3	5	7
USEF1	560	3.957	1.877	1	2	4	5	7
USEF2	560	3.621	1.841	1	2	4	5	7
USEF3	560	4.309	1.813	1	3	5	6	7
USEF4	560	4.441	1.949	1	3	5	6	7
USEF5	560	3.311	1.839	1	2	3	5	7
UNRE1	560	3.955	1.865	1	2	4	5	7
UNRE2	560	3.920	1.900	1	2	4	5	7
UNRE3	560	3.562	1.820	1	2	3	5	7
UNRE4	560	3.461	1.850	1	2	3	5	7
UNRE5	560	4.030	2.024	1	2	4	6	7

*N* indicates the number of observations, Pctl(25) refers to the 25th percentile, Pctl(75) refers to the 75th percentile.

Regarding the multivariate outliers (see Supplementary material for details), we visually inspected large outliers to evaluate the response patterns. This revealed that some of the individuals who presented as outliers, held somewhat contradictory views on specific items, which probably lead to them being classified as outliers. For instance, one responded to items related to the Safety factor, stating, “I have to worry too often whether I might download malicious programs”, with a strong agreement, while also responding to, “I have to worry too often whether I might receive malicious software”, with a strong disagreement and so on. This seemed meaningful to us, as an individual may be confident in their ability to avoid downloading malicious content due to high digital competency for example, but at the same time is also aware of the lack of control over the content they receive online. Given that the outliers we examined gave no clear indication of erratic responses or “respondent fatigue”, we made a decision not to remove the outliers.

Finally, we generated the correlation plot, which allowed the visual inspection of patterns of correlations among variables (see Supplementary material for details). This led to valuable insights. Notably, distinct patterns of relationships emerged among groups of variables associated with the first-order factors. These patterns were discernible, although to varying degrees among different groups of variables (mostly for the first three factors: Complexity, Conflicts and Insecurity, and least for Social Environment). Further examination showed that correlations between some variables exceeded 0.80.

### 3.2 Initial CFA and reliability analysis

Confirmatory factor analysis was employed to assess the construct validity of the proposed model by Fischer et al. (2021). This was done in four steps. First we assessed the model fit on each of the first order factors separately, followed by the whole first-order model assessment. Given the unsatisfactory fit of the original solution, we employed a post-hoc model specification for the first-order factors. Finally we assessed the first and second-order model. Goodness-of-fit tests were applied to each of these.

The 10-factor first order model displayed only a partly convincing fit. Across most of the first-order factors, the Comparative Fit Index (CFI) and Standardized Root Mean Residual (SRMR) indicated an acceptable fit, except for the Invasion of Privacy and Safety factors (see Table 2). However, the Root Mean Square Error of Approximation (RMSEA) and the significant χ^2^ statistic suggested a poor fit. Not surprisingly, the sub-scales with sub-optimal values for CFI, correspondingly displayed higher χ^2^ statistic and RMSEA values, which suggests that these models to an even lesser degree capture the underlying relationships among the variables in our dataset. However, it is important to interpret these results with caution, particularly given the relatively large sample size, as the chi-square statistic tends to become significant with large samples, while the RMSEA tends to indicate poor fit in models with limited degrees of freedom (Kenny et al., 2015). In terms of the standardized factor loadings, all items demonstrated highly salient loadings ranging from 0.61 to 0.95, which indicates that the measurement quality of the individual 1st order factors was satisfactory. For detailed information regarding the specific fit indices and standardized factor loadings for individual items related to the first-order factors, please refer to Table 2. Moreover, the comprehensive model involving all ten first-order factors exhibited a mediocre fit χ^2^ = 3,822.883, df = 1,130.000, CFI = 0.88, RMSEA = 0.07, SRMR = 0.06. These results further confirm the discrepancy between the model-implied and observed data, that was observed while inspecting the first order factors. Given the unsatisfactory fit of this model, we performed a post-hoc model specification. We examined the modification indices and found notable expected parameter change values among ten pairs of items (see Supplementary material). Some of these items were very similar in content, while others appeared to implicitly measure additional dimensions. For instance in Complexity sub-scale, consider item 4, which states, “I often do not find enough time to keep up with new functionalities of ICT at work”, while item 5 states “It would take me too long to completely figure out how to use the ICT that are available to me at work”. Although both items pertain to the Complexity factor of the DSS, they also appear to indirectly allude to the temporal aspect associated with complexity, which could be an additional stressor in itself and might have led the participants to interpret these items in such a way. Therefore, we allowed the residual variance correlations for the abovementioned items, which greatly improved the model fit (see Table 3). Lastly, the second-order model, following Fischer et al. (2021) approach, was estimated and demonstrated a similarly mediocre fit as the first order model (see Table 3). Moreover, we conducted a scaled chi-square difference test for the second-order model vs. the first-order model, which showed that the constraints imposed by the second-order model were inadmissible (Δχ^2^ = 547.03, *p*-value 0.000, df = 35).

**Table 2:** Assessment of measurement quality for each subscale.

	χ^2^*	**RMSEAr**	**CFIr**	**SRMR**	**λ_1_**	**λ_2_**	**λ_3_**	**λ_4_**	**λ_5_**	**α**	**ω**
Complexity	194.92	0.30	0.89	0.05	0.83	0.84	0.85	0.83	0.85	0.92	0.92
Conflicts	29.21	0.13	0.98	0.01	0.80	0.92	0.91	0.93	0.92	0.95	0.95
Insecurity	32.44	0.17	0.96	0.04	0.64	0.95	0.88	0.86	0.89	0.92	0.93
Invasion of privacy	248.44	0.35	0.82	0.08	0.73	0.79	0.83	0.84	0.79	0.90	0.90
Overload	125.25	0.23	0.91	0.07	0.90	0.91	0.62	0.70	0.66	0.88	0.88
Safety	163.25	0.31	0.85	0.07	0.84	0.84	0.71	0.83	0.74	0.89	0.89
Social environment	51.28	0.14	0.95	0.04	0.61	0.76	0.63	0.81	0.80	0.85	0.85
Tech support	44.05	0.14	0.97	0.03	0.74	0.84	0.93	0.91	0.77	0.92	0.93
Usefulness	63.60	0.16	0.95	0.04	0.87	0.88	0.73	0.66	0.68	0.88	0.88
Unreliability	47.28	0.15	0.97	0.03	0.82	0.88	0.85	0.90	0.76	0.92	0.92

λ_1_, …λ_5_: standardized factor loadings, RMSEAr, Root Mean Square Error of Approximation Robust; CFIr, Comparative Fit Index Robust; SRMR, standardised root mean square residual; α, Cronbach's alpha; ω_cat_, McDonald's omega categorical. Gray shading highlights values in the table that meet the specified thresholds. *all χ^2^ *p* < 0.001.

**Table 3:** Model fit indexes for the three models.

	χ^2^*	**df**	**RMSEAr**	**CFIr**	**SRMR**
First order model	3,822.883	1,130.000	0.07	0.88	0.06
First order model with MI	2,637.978	1,120.000	0.05	0.94	0.07
Second order model	4,393.705	1,165.000	0.07	0.87	0.09

* all χ^2^ *p* < 0.001, dr, degrees of freedom; RMSEAr, Root Mean Square Error of Approximation Robust; CFr, Comparative Fit Index Robust; SRMR, standardised root mean square residual.

We proceeded by assessing Cronbach's α along with its confidence intervals (Tables 2, 4) and McDonald's ω for each of the ten sub-scales. The results revealed high internal consistency in both measures across all scales. Notably, the lowest values observed were for the Social Environment factor, with both α and ω coefficients of 0.85. Additionally, the Conflicts factor demonstrated particularly high levels of internal consistency with values of 0.95 for both Cronbach's α McDonald's ω. These findings indicate a high level of internal consistency reliability within the sub-scales. Regarding the reliability of the second-order construct, we calculated ω hierarchical, which was 0.89.

**Table 4:** Confidence intervals for Cronabach's alpha.

**Sub-scale**	**Lower CI α**	**Upper CI α**
Complexity	0.91	0.93
Conflicts	0.95	0.96
Insecurity	0.91	0.93
Invasion of Privacy	0.88	0.91
Overload	0.86	0.90
Safety	0.88	0.91
Social environment	0.82	0.87
Tech support	0.91	0.93
Usefulness	0.86	0.89
Unreliability	0.91	0.93

### 3.3 Exploratory factor analysis

Given the unsatisfactory fit of the original scale, we saw it necessary to investigate the data further with an exploratory factor analysis (EFA) in the third part of our study. We proceeded with calculating the Kaiser-Mayer-Olkin (KMO) and Bartlett's test of sphericity to test for adequacy of the data for performing the EFA. The KMO value was 0.93, which is considered “superb” by Kaiser and Rice (1974), and KMO values for all individual items were > .89, which is considerably over the cut of value of 0.50. Bartlett's test of sphericity yielded a χ^2^(49) = 565.04, *p* = <0.01 indicating that the correlations between variables were sufficient to perform a factor analysis. Details and the R code used, can be found in the Supplementary material. After that, we proceeded with an Exploratory Factor Analysis (EFA) employing a direct oblimin rotation. Our aim was to permit correlation among the primary factors. We employed ML estimation for this process. Regarding the factor extraction, the parallel analysis indicated the presence of 9 factors. However, the resulting 9-factor solution did not yield a meaningful pattern, and involved merging of some of the original factors into one, such as Usefulness and Complexity, Overload and Social environment (this was the case for both factor 6 and 9, with varied items), and Conflicts with Social environment. This lead us to dismiss it as a viable option. Subsequently, we visually assessed the scree plot, which suggested a potential for 7 factors. Unfortunately, the 7-factor solution contained many cross-loading items, which lead us to disregard this solution as well. Kaiser's criterion suggested an 8-factor solution as an alternative. Consequently, we proceeded with an in-depth evaluation of this solution. The 8-factor solution displayed fewer cross-loading items and appeared the most meaningful and closely aligned solution when compared to the original model. Through an iterative process, cross-loadings were systematically eliminated, and items with factor loadings exceeding .55 were retained. (see Table 5 for standardized factor loadings of the 8 factor model; further details and the annotated R code can be found in the Supplementary material). The eight factors included Complexity, Conflicts, Insecurity, Invasion of Privacy, Overload, Safety, Technical Support and Unreliability, comprising a total of 37 items. The inter-factor correlations spanned from 0.21 to 0.68. Certain factors displayed distinct clustering, exhibiting relatively modest correlations with other factors. For instance, the Conflicts factor stood out with relatively low correlations with other factors. On the other hand, certain factors exhibited moderately high correlations with specific counterparts. This was particularly evident in the case of Technical Support, which exhibited strong correlations with Unreliability and Complexity, as well as with Overload. Standardized factor loadings of the eight factor solution are presented in Table 5. For more details about the iterative item deletion process and the r code used, please see the Supplementary material. Both Cronbach's alpha and McDonald's omega were excellent for the 8 factor solution (see details in Table 6). We found no issues when examining discriminant validity for the new solution, with inter-factor correlations and corresponding CIs all under .80, a cutoff generally considered as unproblematic in the literature (Rönkkö and Cho, 2022). For more details see Supplementary material titled *Discriminant Validity*.

**Table 5:** Standardized factor loadings of the eigth factor soultion in EFA.

**A factor analysis table from the psych package in R**								
**1st order factors**	Conf*	**Unre**	**Comp**	**Inse**	**Priv**	**Tech**	**Safe**	**Over**
COMP1	-0.02	0.04	**0.78**	0.05	-0.07	0.04	0.03	0.03
COMP2	-0.03	0.02	**0.80**	0.00	-0.04	0.05	0.08	0.00
COMP3	0.00	0.04	**0.80**	0.06	-0.05	0.00	0.02	0.05
COMP4	0.08	0.02	**0.73**	-0.06	0.14	-0.03	-0.04	0.11
COMP5	0.05	-0.02	**0.84**	-0.01	0.09	0.01	-0.02	-0.03
CONF1	**0.72**	0.07	0.13	0.04	-0.02	0.01	0.03	0.04
CONF2	**0.93**	0.03	0.00	-0.02	-0.03	-0.01	0.01	-0.01
CONF3	**0.93**	-0.01	0.00	-0.03	0.05	-0.01	-0.01	-0.04
CONF4	**0.92**	0.01	0.00	0.01	0.00	-0.01	0.01	0.01
CONF5	**0.89**	-0.04	-0.02	0.02	0.00	0.04	0.02	0.04
INSE2	0.01	0.00	0.03	**0.93**	0.03	-0.02	-0.02	0.02
INSE3	0.02	-0.06	0.04	**0.84**	-0.01	0.07	0.00	0.05
INSE4	-0.03	0.04	-0.05	**0.89**	0.00	-0.01	0.03	-0.05
INSE5	0.00	0.03	-0.01	**0.88**	0.01	-0.03	0.04	-0.01
PRIV1	0.09	-0.03	0.10	0.11	**0.69**	0.10	-0.11	0.02
PRIV2	0.06	0.01	0.05	0.06	**0.79**	0.07	-0.10	0.01
PRIV3	-0.03	0.02	0.01	-0.01	**0.72**	-0.02	0.26	-0.05
PRIV4	-0.01	0.11	-0.02	0.02	**0.76**	-0.03	0.06	0.03
PRIV5	-0.02	-0.01	0.00	-0.04	**0.67**	0.01	0.19	0.08
OVER1	0.02	0.02	0.07	-0.04	-0.04	0.04	0.01	**0.84**
OVER2	-0.06	0.03	0.06	0.04	0.01	0.02	0.01	**0.87**
OVER3	0.19	0.08	-0.15	0.06	0.14	-0.05	0.01	**0.54**
OVER4	0.17	0.00	-0.04	0.10	0.03	0.05	0.02	**0.55**
SAFE1	0.06	-0.05	0.00	0.05	0.11	0.11	**0.63**	0.06
SAFE2	0.03	-0.01	-0.04	0.06	0.06	0.06	**0.66**	0.10
SAFE3	0.04	0.04	-0.04	0.01	0.14	0.02	**0.58**	0.05
SAFE4	-0.01	0.06	0.03	0.02	0.02	-0.03	**0.85**	0.00
SAFE5	0.06	0.00	0.10	0.04	-0.03	0.03	**0.77**	-0.05
TECH2	0.07	0.02	0.02	0.01	0.02	**0.73**	0.04	0.06
TECH3	-0.01	-0.04	0.01	-0.04	0.03	**0.95**	0.00	0.00
TECH4	-0.01	0.08	-0.01	0.03	-0.01	**0.90**	-0.04	-0.02
TECH5	0.00	0.06	0.03	0.01	-0.03	**0.67**	0.12	0.03
UNRE1	0.00	**0.71**	0.06	-0.01	0.10	0.07	-0.03	0.02
UNRE2	-0.04	**0.87**	0.07	0.05	0.04	-0.01	-0.04	-0.02
UNRE3	0.09	**0.85**	-0.08	0.00	-0.03	0.04	-0.01	0.01
UNRE4	0.00	**0.85**	0.02	0.00	-0.03	0.04	0.07	0.00
UNRE5	-0.06	**0.64**	0.05	-0.04	0.01	0.00	0.07	0.18
	**Conf**	**Unre**	**Comp**	**Inse**	**Priv**	**Tech**	**Safe**	**Over**
SS loadings	4.26	3.57	3.54	3.37	3.16	3.12	3.08	2.59
Proportion Var	0.12	0.10	0.10	0.09	0.09	0.08	0.08	0.07
Cumulative Var	0.12	0.21	0.31	0.40	0.48	0.57	0.65	0.72
Conflicts	1.00	0.22	0.31	0.21	0.37	0.32	0.26	0.42
Unreliability	0.22	1.00	0.44	0.23	0.29	0.68	0.29	0.55
Complexity	0.31	0.44	1.00	0.23	0.27	0.53	0.28	0.53
Insecurity	0.21	0.23	0.23	1.00	0.34	0.20	0.37	0.30
Privacy	0.37	0.29	0.27	0.34	1.00	0.30	0.50	0.28
TechSupport	0.32	0.68	0.53	0.20	0.30	1.00	0.30	0.50
Safety	0.26	0.29	0.28	0.37	0.50	0.30	1.00	0.28
Overload	0.42	0.55	0.53	0.30	0.28	0.50	0.28	1.00

*First order factors: Conf, Conflicts; Unre, Unreliability; Comp, Complexity; Inse, Insecurity; Priv, Invasion of Privacy; Tech, Technical support; Safe, Safety; Over, Overload.

**Table 6:** Internal consistency reliability measures for the 8 factor solution.

	**Complexity**	**Conflicts**	**Insecurity**	Privacy*	**Overload**	**Safety**	**TechSupport**	**Unreliability**
α	0.92	0.95	0.94	0.90	0.86	0.89	0.92	0.92
ω*	0.92	0.95	0.94	0.90	0.87	0.89	0.92	0.92

*α, Cronbach's alpha; ω, McDonald's omega; Privacy, Invasion of Privacy; TechSupport, Technical support.

## 4 Discussion

The aim of this study was to investigate the psychometric properties of the translated Digital Stressors Scale among a sample of Norwegian employees, by examining the factor structure of the DSS, as well as internal consistency. The original solution (Fischer et al., 2021) could not be entirely replicated in the Norwegian context. Therefore, we propose a shorter 8 factor solution with 37 of the original 50 items.

### 4.1 Factor structure

Contrary to the original solution proposed by Fischer et al. (2021), the CFA results in our study revealed that the factor structure of the original DSS solution did not replicate within a Norwegian sample. To explore this further, we examined the residuals, modification indices and expected parameter change values. This was done in order to identify the specific parameters, which would lead to an improved fit of the model. *Post-ho*c specification resulted in an improved fit, however, it is, generally, advisable to exercise caution when considering the use of modification indices for several reasons, which are thoroughly elaborated on in the literature (Brown, 2015; Kline, 2016; Hoyle, 2023). These include the potential risk of overfitting and the possibility of capitalising on chance relationships specific to the data set (Hoyle, 2023). As the objective is to provide a more parsimonious explanation of the relationships between variables, correlating errors is often seen as unhelpful, both in theoretical, but also practical sense. As a result, the model may demonstrate a stronger fit within the existing sample, as it is in our case, but potentially perform inadequately when applied to new samples, leading to a decrease in its overall generalizability. For this reason, as recommended by Hoyle (2023) model revisions with the use of modification indices should be guided by and grounded in theory, as additional covariance among items usually suggests the presence of common underlying factors or sources of variability that have not been explicitly accounted for in the model (Hoyle, 2023). In other words, there might be unmodeled factors or constructs that influence the observed variables, which may indicate a lack of conceptual clarity and may lead to poor validity (Kline, 2016). Results in our study suggest that this might be the case with the DSS and should be addressed in future studies and different contexts (Kline, 2016).

Due to the unsatisfactory fit of the CFAs, we proceeded with an EFA, and landed on an 8 factor solution, comprising 37 items, which includes Complexity, Conflicts, Insecurity, Invasion of privacy, Overload, Safety, Technical support and Unreliability factors of the original solution. The 8-factor model explained 72% of variance and showed excellent reliability for each sub-scale. We encountered challenges with two latent constructs, namely, Social Environment and Usefulness. Some items from the Social environment factor cross loaded on items from the Overload factor, while some items from the Usefulness factor cross-loaded to Technical Support factor (see Supplementary material for details). This suggested a conceptual overlap, or lack of clarity that probably affected the strength of the relationship between these items and the latent factors. This finding was surprising, as it would have been more logical if the overlap concerned the Unreliability and Technical Support factors, which may be seen as a function of one another, i.e., that technical support is necessary when the technology is unreliable. This, however, was not the case. It is important to note that, in terms of content, both Social Environment and Usefulness, represent important digital stressors. Therefore, the results from our study suggest a need to improve item clarity and overall content validity of these sub-scales in order to improve the validity of these constructs. Additionally, low factor loadings led us to remove the rest of the items in these two scales, as well as three items from other sub-scales (Item 1 from the Invasion of Privacy scale, item 5 from the Overload scale, and item 1 from the Technical Support scale.).

### 4.2 Conceptual considerations for future research

One of the critical considerations in scale development and validation is the model specification. In this study, one noteworthy conceptual concern arose regarding the second order construct of the DSS. The issue concerns the distinction between reflective and formative models. Reflective models assume that the latent factors cause the observed items, while in formative models, factors are caused by the observed items (Kline, 2016). In the case of the DSS, first order factors measure various digital stressors, such as Complexity, Conflicts, etc., through a reflective measurement model. However, the authors define the second order model which measures digital stress, also through a reflective measurement model, rather than a formative one, after comparing the difference between path coefficients and the explained variance between a reflective and a formative model (Fischer et al., 2021). The rationale was described as: “Therefore, as there is no clear indication for a formative specification, a reflective specification was chosen instead, in line with Ragu-Nathan et al. (2008)” (Fischer et al., 2021, p. 11). This, in our opinion demands closer examination. For instance, let's consider an item from the Complexity scale, “I often find it too complicated to accomplish a task using the ICT that are available to me at work”. It is reasonable to regard this item as an observable manifestation of the latent construct of Complexity. However, if we look at the second order factor of digital stress, it becomes difficult to see digital stress, which is typically a response to stressors, as a cause for Complexity of the ICT solutions at work. Closer content analysis of the various items across the DSS shows that some items are more a measure of digital stress, like “I feel that my private life suffers due to ICT enabling work-related problems to reach me everywhere”, while others are better suited for measuring digital stressors, like “I feel that the ICT that are available to me at work are too confusing”^3^. While the advantage of the DSS compared to previous scales is that the items are intended to capture the transactional nature of digital stress (Fischer et al., 2021) this conceptual inconsistency highlights the need for a deeper examination of the model specification, but also a additional content analysis of the items of the DSS.

### 4.3 Reliability and validity

We examined the reliability of the sub-scales of the DSS and estimated both Cronbach's alpha and McDonald's omega as parameters of internal consistency. The results were satisfactory for all the sub-scales. These results support the disaggregated approach, meaning that the sub-scales are conceptually homogeneous. Coupled with the salient standardized factor loadings, this indicates that the constructs are robustly measured, suggests that the sub-scales can be used as the independent measures for the different digital stressors and further the understanding of the nature of digital stress phenomenon.

### 4.4 Strengths and limitations

The study has some notable strengths. This is the first study to comprehensively investigate the psychometric properties of the DSS outside of the US. The study, addresses a critical gap in the scientific literature, by providing the first translation and validation of the DSS in a Norwegian context. This enables the use of DSS beyond the original context. Second, the study employs rigorous methodology by, first translating the instrument according to recommended guidelines, and further by using the covariance-based latent modelling, such as confirmatory factor analysis for construct validation. Third, all the code used in the study has been included in the Supplementary material, with the intention to aid replicability and transparency in research practices, as well as to offer a practical guide for future studies that wish to translate and validate existing scales. Furthermore, the study points to the challenges related to the conceptualisation of the second-order model of the DSS (reflective vs formative), and calls for the closer examination of the model specification and further content analysis. The study represents an important milestone in the validation of the DSS which will, hopefully, lead to the DSS becoming a more robust tool for measuring digital stressors. Several limitations should be considered when interpreting the results from this study. First, we performed the study on a convenience sample from the general population in Norway, which may limit the generalizability of the results beyond the context of this study. Second, the study sample was predominantly female, which may further limit the generalizability of the results. Finally, the scale items were translated to Norwegian, which might have introduced variation in the interpretation of the items, although we aimed for semantic equivalence throughout the translation process.

## 5 Conclusion

To the best of our knowledge, this is the first translation and validation of the DSS on the sample outside of the US. Given that the original solution of the DSS could not be entirely replicated in the Norwegian context, we performed an exploratory factor analysis and propose a shorter 8 - factor solution with 37 of the original 50 items of the DSS. The sub-scales of the DSS may serve as measures of individual stressors in a disaggregated approach, still we advise that this is done with caution due to varying degrees of conceptual clarity for some of the subs-scales.

It is evident that some refinement to fully capture the conceptual nuances remains, and further validation studies of the 8-factor model proposed in our study are needed. As the field of digital stress (technostress) is still relatively young and continues to progress, it is essential to recognise that the path toward a comprehensive understanding of the digital stress is ongoing and that further conceptual work and refinement of measurement models is needed for the development of robust tools. This study can be considered as an important step toward a psychometrically robust Norwegian version of Digital Stressors Scale.

## Data availability statement

The datasets presented in this study can be found in online repositories. The names of the repository/repositories and accession number(s) can be found below: https://osf.io/3mcaf/?view_only=fcdeba789fad4b138b54ce7f96ef1e95.

## Ethics statement

Ethical review and approval was not required for the study on human participants in accordance with the local legislation and institutional requirements. The studies were conducted in accordance with the local legislation and institutional requirements. The participants provided their written informed consent to participate in this study.

## Author contributions

AS: Conceptualization, Data curation, Formal analysis, Investigation, Methodology, Project administration, Validation, Writing – original draft, Writing – review & editing, Visualization. NF: Formal analysis, Methodology, Supervision, Validation, Writing – review & editing. KB: Conceptualization, Investigation, Methodology, Supervision, Validation, Writing – review & editing.

## Footnotes

^1^We will use these terms interchangeably thought the text.

^2^It should be noted that RMSEA tends to be inflated in simpler models with fewer degrees of freedom.

^3^“I feel that my private life suffers due to ICT enabling work-related problems to reach me everywhere” is a part of the Conflicts sub-scale and “I feel that the ICT that are available to me at work are too confusing” is a part of the Complexity sub-scale.
