# Supplementary material for: Measuring digital stress in Norway: translation and validation of the Digital Stressors Scale
Source: Front Psychol. 2024 Feb 9;15:1297194. doi: 10.3389/fpsyg.2024.1297194 (PMC10884271; doi:10.3389/fpsyg.2024.1297194)
Supplement: Supplementary file 1 [file Data_Sheet_1.PDF]

The Digital Stressors Scale

Innledning

Informasjons- og kommunikasjonsteknologi (IKT) blir gradvis mer utbredt i samfunnet og det er ingen tvil om at IKT har blitt en viktig faktor i mange menneskers liv – nå er det vanskelig å forestille seg et liv uten IKT. Individer, organisasjoner og samfunn nyter godt av IKT-bruk på mange måter; økt tilgang til informasjon i tillegg til økt produktivitet er bare noen eksempler. I denne sammenhengen vil vi undersøke dine oppfatninger angående digitalisering av dine profesjonelle omgivelser.

Forklaring IKT

Vi ber deg være klar over at forkortelsen IKT, "Informasjons- og kommunikasjonsteknologi", blir brukt for å referere til mange ulike teknologier som vanligvis finnes i kontormiljøer.

Vanligvis, når vi refererer til IKT, mener vi:

- Mobilteknologi (f.eks smarttelefon, portabel PC, pager, PDA)
- Nettverksteknologi (f.eks internett, intranett, VPN)
- Kommunikasjonsteknologi (f.eks e-post, talepost)
- Bedriftsprogramvare (f.eks PeopleSoft®, SAP®, Oracle® applikasjoner)

- Generisk programvareteknologi (f.eks tekstbehandling, regneark, presentasjonsprogamvare)
- Samarbeidsteknologi (f.eks "instant messaging", videokonferanser, telekonferanser)
- Annen arbeidsspesifikk teknologi

Den faktiske undersøkelsen vil begynne på neste side. Spørsmålene vil omhandle din oppfatning av teknologi i ditt profesjonelle miljø, ditt personlige velvære, samt egenskaper ved din væremåte slik du selv vurderer dem.

Hvis du er usikker på hvordan du skal besvare et spørsmål, enten fordi det ikke er klart for deg hvordan du skal svare eller fordi utsagnet ikke gjelder din situasjon, velg «vet ikke». Takk!

Subskala 10-faktor løsning	Items	1	2	3	4	5	6	7
Kompleksitet (COMP 1)	Jeg finner at det ofte er for komplisert å løse en oppgave ved å bruke IKT som er tilgjengelig for meg på jobben.							
Kompleksitet (COMP 2)	Jeg trenger ofte mer tid enn forventet til å løse en oppgave ved å bruke IKT som er tilgjengelig på jobb.							
Kompleksitet (COMP 3)	Jeg føler at tilgjengelig IKT på jobben er for forvirrende.							
Kompleksitet (COMP 4)	Jeg finner ofte ikke tid nok til å følge med på ny IKT funksjonalitet på jobben.							
Kompleksitet (COMP 5)	Det ville tatt meg for lang tid å finne fullstendig ut av hvordan jeg skal bruke IKT som er tilgjengelig på jobb.							
Konflikter (CONF 1)	Jeg føler at privatlivet mitt lider under at IKT gjør at arbeidsrelaterte problemer kan nå meg hvor som helst.							
Konflikter (CONF 2)	Det er for vanskelig for meg å skille mellom privatliv og arbeid på grunn av IKT.							

Konflikter (CONF 3)	IKT gjør det vanskeligere å skape klare grenser mellom mitt privatliv og arbeidsliv.							
Konflikter (CONF 4)	Min jobb-fritid-balanse lider på grunn av IKT.							
Konflikter (CONF 5)	Allestedsnærværende IKT forstyrrer min jobb-fritid-balanse.							
Usikkerhet (INSE 1)	Jeg føler at jobben min er truet på grunn av IKT.							
Usikkerhet (INSE 2)	Jeg frykter at jeg kan bli erstattet på jobb på grunn av økt standardisering av arbeidsprosesser som er muliggjort av IKT.							
Usikkerhet (INSE 3)	Jeg kan ikke være optimistisk med tanke på min langsiktige jobbtrygghet på grunn av IKT automatisering.							
Usikkerhet (INSE 4)	Jeg frykter at jeg kunne erstattes av maskiner.							
Usikkerhet (INSE 5)	Jeg frykter at digitalisering vil koste meg jobben min.							
Forstyrrelse av privatlivet (PRIV 1)	Jeg frykter at min bruk av IKT er mindre konfidensiell enn jeg ville likt.							
Forstyrrelse av privatlivet (PRIV 2)	Jeg frykter at informasjonen jeg utveksler ved bruk av IKT ikke er så beskyttet som jeg ville likt.							
Forstyrrelse av privatlivet (PRIV 3)	Jeg frykter at ondsinnede utenforstående (f.eks. hackere) lett kan kopiere min identitet på grunn av IKT.							
Forstyrrelse av privatlivet (PRIV 4)	Min personlige informasjon er for lett tilgjengelig på grunn av IKT.							
Forstyrrelse av privatlivet (PRIV 5)	Jeg frykter at mine personlige data lett kan bli stjålet av andre online.							
Overbelastning (OVER 1)	På grunn av IKT har jeg for mye å gjøre.							
Overbelastning (OVER 2)	På grunn av IKT har jeg for mange ulike ting å gjøre på jobb.							
Overbelastning (OVER 3)	IKT gjør at det er for lett for andre individer å sende meg mer arbeid.							
Overbelastning (OVER 4)	Jeg har aldri ledig tid, fordi timeplanen min er for tett organisert av IKT.							

Overbelastning (OVER 5)	Det kommer en konstant strøm av arbeidsrelatert informasjon gjennom IKT som jeg bare ikke kan følge med på.							
Sikkerhet (SAFE 1)	Jeg må bekymre meg for ofte om jeg kan komme til å laste ned ondsinnet programvare.							
Sikkerhet (SAFE 2)	Jeg må bekymre meg for ofte om jeg kan komme til å motta ondsinnet e-post.							
Sikkerhet (SAFE 3)	Jeg frykter at hackere kan få tilgang til bedriftshemmeligheter på grunn av en tabbe jeg gjør.							
Sikkerhet (SAFE 4)	Jeg føler meg engstelig når jeg får en e-post fra noen jeg ikke kjenner siden det kunne være et ondsinnet angrep.							
Sikkerhet (SAFE 5)	E-post fra avsendere jeg ikke kjenner gjør meg nervøs.							
Sosialt miljø (SOCI 1)	På grunn av IKT har jeg for mye å gjøre med andres problemer.							
Sosialt miljø (SOCI 2)	Jeg tenker at IKT genererer en for stor forventning om at jeg må være mulig å nå overalt og når som helst.							
Sosialt miljø (SOCI 3)	For mye tid går tapt på jobb på grunn av irrelevant kommunikasjon med andre folk på sosiale medier.							
Sosialt miljø (SOCI 4)	Jeg føler at IKT skaper uønskede sosiale normer (f.eks. at e-post skal besvares umiddelbart).							
Sosialt miljø (SOCI 5)	Det er for vanskelig å ta en pause fra sosiale interaksjoner på jobb på grunn av IKT kommunikasjonsmuligheter.							
Teknisk støtte (TECH 1)	Jeg må bekymre meg for IKT-relaterte problemer siden organisasjonen vår ikke tilbyr nok støtte til å fjerne dem.							
Teknisk støtte (TECH 2)	I tilfelle IKT-relaterte problemer, skjer det for ofte at det ikke er nok støtte på jobb.							
Teknisk støtte (TECH 3)	Jeg tenker at det hender for ofte at teknisk støtte ikke er tilgjengelig når jeg trenger det.							
Teknisk støtte (TECH 4)	Jeg må ofte vente lenge fordi tekniske problemer ikke kan løses adekvat i vår organisasjon.							

Teknisk støtte (TECH 5)	Jeg frykter at et teknisk problem jeg har på jobb ikke ville kunne bli løst av andre på jobb.							
Nytte (USEF 1)	Jeg tenker at kravene på jobben min og funksjonene gitt av IKT-løsningene jeg bruker ikke passer godt nok sammen.							
Nytte (USEF 2)	Jeg tenker at jeg ikke får nok nytte av å bruke IKT som jeg er gitt på jobben min til å utføre oppgavene mine.							
Nytte (USEF 3)	IKT som jeg bruker på jobb er fullt av for mange funksjoner som jeg aldri trenger.							
Nytte (USEF 4)	Det trengs for mange ulike systemer til å fullføre oppgavene jeg må gjøre på en vanlig dag på jobb.							
Nytte (USEF 5)	Jeg tenker at mesteparten av IKT jeg har fått på jobb ikke er nyttig nok og at jeg kunne jobbet uten det.							
Upålitelighet (UNRE 1)	Jeg tenker at jeg for ofte blir konfrontert med uventet fungering fra IKT som jeg bruker på jobb (f. eks nedetid eller lang responstid).							
Upålitelighet (UNRE 2)	Jeg tenker at jeg taper for mye tid på grunn av teknisk svikt.							
Upålitelighet (UNRE 3)	Jeg tenker at jeg bruker for mye tid på å prøve å fikse tekniske feil.							
Upålitelighet (UNRE 4)	For mye av min tid på jobb kastes bort på å skulle håndtere upålitelig IKT.							
Upålitelighet (UNRE 5)	Det daglige bryderiet med IKT (f.eks. trege programmer eller uventet atferd) plager meg virkelig.							

Subscales and items for the 8 factor solution

Subskala 8-faktor løsning	Items	1	2	3	4	5	6	7
Kompleksitet (COMP 1)	Jeg finner at det ofte er for komplisert å løse en oppgave ved å bruke IKT som er tilgjengelig for meg på jobben.							
Kompleksitet (COMP 2)	Jeg trenger ofte mer tid enn forventet til å løse en oppgave ved å bruke IKT som er tilgjengelig på jobb.							
Kompleksitet (COMP 3)	Jeg føler at tilgjengelig IKT på jobben er for forvirrende.							

Kompleksitet (COMP 4)	Jeg finner ofte ikke tid nok til å følge med på ny IKT funksjonalitet på jobben.							
Kompleksitet (COMP 5)	Det ville tatt meg for lang tid å finne fullstendig ut av hvordan jeg skal bruke IKT som er tilgjengelig på jobb.							
Konflikter (CONF 1)	Jeg føler at privatlivet mitt lider under at IKT gjør at arbeidsrelaterte problemer kan nå meg hvor som helst.							
Konflikter (CONF 2)	Det er for vanskelig for meg å skille mellom privatliv og arbeid på grunn av IKT.							
Konflikter (CONF 3)	IKT gjør det vanskeligere å skape klare grenser mellom mitt privatliv og arbeidsliv.							
Konflikter (CONF 4)	Min jobb-fritid-balanse lider på grunn av IKT.							
Konflikter (CONF 5)	Allestedsnærværende IKT forstyrrer min jobb-fritid-balanse.							
Usikkerhet (INSE 2)	Jeg frykter at jeg kan bli erstattet på jobb på grunn av økt standardisering av arbeidsprosesser som er muliggjort av IKT.							
Usikkerhet (INSE 3)	Jeg kan ikke være optimistisk med tanke på min langsiktige jobbtrygghet på grunn av IKT automatisering.							
Usikkerhet (INSE 4)	Jeg frykter at jeg kunne erstattes av maskiner.							
Usikkerhet (INSE 5)	Jeg frykter at digitalisering vil koste meg jobben min.							
Forstyrrelse av privatlivet (PRIV 1)	Jeg frykter at min bruk av IKT er mindre konfidensiell enn jeg ville likt.							
Forstyrrelse av privatlivet (PRIV 2)	Jeg frykter at informasjonen jeg utveksler ved bruk av IKT ikke er så beskyttet som jeg ville likt.							
Forstyrrelse av privatlivet (PRIV 3)	Jeg frykter at ondsinnede utenforstående (f.eks. hackere) lett kan kopiere min identitet på grunn av IKT.							
Forstyrrelse av privatlivet (PRIV 4)	Min personlige informasjon er for lett tilgjengelig på grunn av IKT.							
Forstyrrelse av privatlivet (PRIV 5)	Jeg frykter at mine personlige data lett kan bli stjålet av andre online.							
Overbelastning (OVER 1)	På grunn av IKT har jeg for mye å gjøre.							

Overbelastning (OVER 2)	På grunn av IKT har jeg for mange ulike ting å gjøre på jobb.							
Overbelastning (OVER 3)	IKT gjør at det er for lett for andre individer å sende meg mer arbeid.							
Overbelastning (OVER 4)	Jeg har aldri ledig tid, fordi timeplanen min er for tett organisert av IKT.							
Sikkerhet (SAFE 1)	Jeg må bekymre meg for ofte om jeg kan komme til å laste ned ondsinnet programvare.							
Sikkerhet (SAFE 2)	Jeg må bekymre meg for ofte om jeg kan komme til å motta ondsinnet e-post.							
Sikkerhet (SAFE 3)	Jeg frykter at hackere kan få tilgang til bedriftshemmeligheter på grunn av en tabbe jeg gjør.							
Sikkerhet (SAFE 4)	Jeg føler meg engstelig når jeg får en e-post fra noen jeg ikke kjenner siden det kunne være et ondsinnet angrep.							
Sikkerhet (SAFE 5)	E-post fra avsendere jeg ikke kjenner gjør meg nervøs.							
Teknisk støtte (TECH 2)	I tilfelle IKT-relaterte problemer, skjer det for ofte at det ikke er nok støtte på jobb.							
Teknisk støtte (TECH 3)	Jeg tenker at det hender for ofte at teknisk støtte ikke er tilgjengelig når jeg trenger det.							
Teknisk støtte (TECH 4)	Jeg må ofte vente lenge fordi tekniske problemer ikke kan løses adekvat i vår organisasjon.							
Teknisk støtte (TECH 5)	Jeg frykter at et teknisk problem jeg har på jobb ikke ville kunne bli løst av andre på jobb.							
Upålitelighet (UNRE 1)	Jeg tenker at jeg for ofte blir konfrontert med uventet fungering fra IKT som jeg bruker på jobb (f. eks nedetid eller lang responstid).							
Upålitelighet (UNRE 2)	Jeg tenker at jeg taper for mye tid på grunn av teknisk svikt.							
Upålitelighet (UNRE 3)	Jeg tenker at jeg bruker for mye tid på å prøve å fikse tekniske feil.							

Upålitelighet (UNRE 4)	For mye av min tid på jobb kastes bort på å skulle håndtere upålitelig IKT.							
Upålitelighet (UNRE 5)	Det daglige bryderiet med IKT (f.eks. trege programmer eller uventet atferd) plager meg virkelig.							
