# Supplementary material for: Measuring digital stress in Norway: translation and validation of the Digital Stressors Scale
Source: Front Psychol. 2024 Feb 9;15:1297194. doi: 10.3389/fpsyg.2024.1297194 (PMC10884271; doi:10.3389/fpsyg.2024.1297194)
Supplement: Supplementary file 2 [file Data_Sheet_2.PDF]

Discriminant validity

Sevic A., Foldnes N., Broennick K.

Table of contents

Discriminant validity assessment for the 8-factor solution 1

Discriminant validity assessment for the 8-factor solution

```
library(psych)
library(haven)
library(REdaS)
```

```
Loading required package: grid
```

```
library(readxl)
library(GPArotation)
```

```
Attaching package: 'GPArotation'
```

```
The following objects are masked from 'package:psych':

    equamax, varimin
```

```
library(faoutlier)
```

```
Loading required package: sem
```

```
Loading required package: mvtnorm
```

```
Loading required package: parallel
```

```r
library(lavaan)
```

```
This is lavaan 0.6-17
lavaan is FREE software! Please report any bugs.


Attaching package: 'lavaan'


The following objects are masked from 'package:sem':

    cfa, sem


The following object is masked from 'package:psych':

    cor2cov
```

```r
#import data
nor <- read_sav("onlydss.sav")
```

```r
# Define a combined CFA model for all subscales
combinedCfaModel <- 'COMP =~ COMP1 + COMP2 + COMP3 + COMP4 + COMP5
                    CONF =~ CONF1 + CONF2 + CONF3 + CONF4 + CONF5
                    INSE =~ INSE2 + INSE3 + INSE4 + INSE5
                    PRIV =~ PRIV1 + PRIV2 + PRIV3 + PRIV4 + PRIV5
                    OVER =~ OVER1 + OVER2 + OVER3 + OVER4
                    SAFE =~ SAFE1 + SAFE2 + SAFE3 + SAFE4 + SAFE5
                    TECH =~ TECH2 + TECH3 + TECH4 + TECH5
                    UNRE =~ UNRE1 + UNRE2 + UNRE3 + UNRE4 + UNRE5'

# Fit the combined CFA model
fitCombined <- cfa(combinedCfaModel, data = nor, estimator = "MLR", std.lv = TRUE)

summary(fitCombined, ci=TRUE)
```

```
lavaan 0.6.17 ended normally after 41 iterations

  Estimator                                         ML
```

```
  Optimization method                           NLMINB
  Number of model parameters                       102

  Number of observations                           560

Model Test User Model:
                                              Standard      Scaled
  Test Statistic                              2296.747    1934.294
  Degrees of freedom                               601         601
  P-value (Chi-square)                           0.000       0.000
  Scaling correction factor                                  1.187
    Yuan-Bentler correction (Mplus variant)

Parameter Estimates:

  Standard errors                             Sandwich
  Information bread                           Observed
  Observed information based on                Hessian

Latent Variables:
                   Estimate  Std.Err  z-value  P(>|z|) ci.lower ci.upper
  COMP =~
    COMP1             1.464    0.057   25.660    0.000    1.352    1.576
    COMP2             1.646    0.055   30.159    0.000    1.539    1.753
    COMP3             1.533    0.049   31.058    0.000    1.437    1.630
    COMP4             1.652    0.064   25.815    0.000    1.527    1.777
    COMP5             1.745    0.058   29.898    0.000    1.631    1.859
  CONF =~
    CONF1             1.501    0.058   25.797    0.000    1.387    1.615
    CONF2             1.660    0.049   33.574    0.000    1.563    1.757
    CONF3             1.758    0.045   39.506    0.000    1.671    1.845
    CONF4             1.645    0.049   33.666    0.000    1.550    1.741
    CONF5             1.667    0.047   35.811    0.000    1.576    1.759
  INSE =~
    INSE2             1.307    0.069   19.080    0.000    1.173    1.442
    INSE3             1.237    0.070   17.756    0.000    1.101    1.374
    INSE4             1.172    0.077   15.259    0.000    1.021    1.323
    INSE5             1.159    0.070   16.607    0.000    1.023    1.296
  PRIV =~
    PRIV1             1.303    0.074   17.538    0.000    1.158    1.449
    PRIV2             1.425    0.068   20.892    0.000    1.292    1.559
    PRIV3             1.406    0.057   24.455    0.000    1.293    1.518
    PRIV4             1.384    0.057   24.317    0.000    1.273    1.496
```

PRIV5	1.323	0.065	20.264	0.000	1.195	1.451
OVER =~						
OVER1	1.704	0.050	34.409	0.000	1.607	1.801
OVER2	1.790	0.048	37.601	0.000	1.697	1.883
OVER3	1.146	0.075	15.342	0.000	0.999	1.292
OVER4	1.169	0.067	17.540	0.000	1.038	1.299
SAFE =~						
SAFE1	1.296	0.076	16.958	0.000	1.146	1.446
SAFE2	1.308	0.069	18.893	0.000	1.172	1.443
SAFE3	1.206	0.070	17.243	0.000	1.069	1.343
SAFE4	1.446	0.074	19.472	0.000	1.300	1.591
SAFE5	1.224	0.083	14.826	0.000	1.062	1.386
TECH =~						
TECH2	1.610	0.056	28.869	0.000	1.501	1.720
TECH3	1.834	0.044	41.354	0.000	1.747	1.921
TECH4	1.804	0.045	40.158	0.000	1.716	1.892
TECH5	1.536	0.060	25.410	0.000	1.417	1.654
UNRE =~						
UNRE1	1.535	0.055	27.687	0.000	1.426	1.643
UNRE2	1.666	0.050	33.647	0.000	1.569	1.763
UNRE3	1.540	0.053	28.815	0.000	1.435	1.644
UNRE4	1.666	0.046	35.869	0.000	1.575	1.757
UNRE5	1.561	0.055	28.322	0.000	1.453	1.669

Covariances:

	Estimate	Std.Err	z-value	P(>|z|)	ci.lower	ci.upper
COMP ~~						
CONF	0.365	0.041	8.789	0.000	0.283	0.446
INSE	0.276	0.044	6.327	0.000	0.190	0.361
PRIV	0.390	0.045	8.642	0.000	0.301	0.478
OVER	0.629	0.032	19.473	0.000	0.566	0.693
SAFE	0.405	0.040	10.082	0.000	0.327	0.484
TECH	0.581	0.034	17.052	0.000	0.515	0.648
UNRE	0.527	0.037	14.406	0.000	0.455	0.599
CONF ~~						
INSE	0.228	0.048	4.752	0.000	0.134	0.322
PRIV	0.417	0.041	10.119	0.000	0.336	0.498
OVER	0.457	0.041	11.087	0.000	0.376	0.538
SAFE	0.359	0.045	7.964	0.000	0.271	0.447
TECH	0.343	0.042	8.240	0.000	0.261	0.425
UNRE	0.261	0.044	5.870	0.000	0.174	0.348
INSE ~~						
PRIV	0.409	0.038	10.652	0.000	0.334	0.484

```
    OVER              0.344    0.042    8.168    0.000    0.261    0.426
    SAFE              0.446    0.045    9.944    0.000    0.358    0.534
    TECH              0.221    0.042    5.266    0.000    0.139    0.303
    UNRE              0.263    0.041    6.465    0.000    0.183    0.343
  PRIV ~~
    OVER              0.383    0.044    8.687    0.000    0.296    0.469
    SAFE              0.653    0.033   19.714    0.000    0.588    0.718
    TECH              0.394    0.043    9.246    0.000    0.311    0.478
    UNRE              0.397    0.043    9.336    0.000    0.314    0.481
  OVER ~~
    SAFE              0.417    0.043    9.596    0.000    0.332    0.502
    TECH              0.568    0.035   16.144    0.000    0.499    0.637
    UNRE              0.626    0.032   19.629    0.000    0.564    0.689
  SAFE ~~
    TECH              0.403    0.042    9.619    0.000    0.321    0.485
    UNRE              0.403    0.043    9.395    0.000    0.319    0.487
  TECH ~~
    UNRE              0.726    0.027   26.434    0.000    0.673    0.780

Variances:
                   Estimate  Std.Err  z-value  P(>|z|) ci.lower ci.upper
   .COMP1             0.992    0.117    8.473    0.000    0.762    1.221
   .COMP2             1.121    0.122    9.158    0.000    0.881    1.361
   .COMP3             0.884    0.088   10.044    0.000    0.712    1.057
   .COMP4             1.204    0.147    8.178    0.000    0.916    1.493
   .COMP5             1.175    0.134    8.789    0.000    0.913    1.437
   .CONF1             1.266    0.103   12.286    0.000    1.064    1.468
   .CONF2             0.528    0.061    8.714    0.000    0.410    0.647
   .CONF3             0.623    0.092    6.756    0.000    0.442    0.803
   .CONF4             0.404    0.048    8.505    0.000    0.311    0.497
   .CONF5             0.492    0.076    6.502    0.000    0.344    0.640
   .INSE2             0.196    0.038    5.127    0.000    0.121    0.271
   .INSE3             0.490    0.090    5.469    0.000    0.314    0.665
   .INSE4             0.442    0.071    6.206    0.000    0.303    0.582
   .INSE5             0.354    0.081    4.353    0.000    0.194    0.513
   .PRIV1             1.355    0.158    8.598    0.000    1.046    1.664
   .PRIV2             1.159    0.147    7.880    0.000    0.871    1.448
   .PRIV3             0.894    0.110    8.154    0.000    0.679    1.109
   .PRIV4             0.854    0.106    8.040    0.000    0.646    1.062
   .PRIV5             1.090    0.127    8.562    0.000    0.841    1.340
   .OVER1             0.674    0.095    7.095    0.000    0.488    0.860
   .OVER2             0.576    0.104    5.524    0.000    0.371    0.780
   .OVER3             2.242    0.148   15.177    0.000    1.953    2.532
```

```
   .OVER4            1.617    0.127   12.738    0.000    1.368    1.866
   .SAFE1            0.711    0.137    5.203    0.000    0.443    0.979
   .SAFE2            0.776    0.139    5.567    0.000    0.503    1.049
   .SAFE3            1.384    0.131   10.606    0.000    1.129    1.640
   .SAFE4            0.967    0.164    5.908    0.000    0.646    1.287
   .SAFE5            1.198    0.165    7.257    0.000    0.874    1.521
   .TECH2            1.188    0.117   10.197    0.000    0.960    1.417
   .TECH3            0.592    0.073    8.141    0.000    0.449    0.734
   .TECH4            0.616    0.083    7.451    0.000    0.454    0.778
   .TECH5            1.523    0.152   10.034    0.000    1.226    1.821
   .UNRE1            1.116    0.107   10.397    0.000    0.906    1.326
   .UNRE2            0.826    0.099    8.325    0.000    0.632    1.021
   .UNRE3            0.936    0.108    8.646    0.000    0.724    1.148
   .UNRE4            0.642    0.068    9.450    0.000    0.509    0.776
   .UNRE5            1.654    0.129   12.829    0.000    1.402    1.907
    COMP             1.000                               1.000    1.000
    CONF             1.000                               1.000    1.000
    INSE             1.000                               1.000    1.000
    PRIV             1.000                               1.000    1.000
    OVER             1.000                               1.000    1.000
    SAFE             1.000                               1.000    1.000
    TECH             1.000                               1.000    1.000
    UNRE             1.000                               1.000    1.000
```

```r
semTools::reliability(fitCombined)
```

```
            COMP      CONF      INSE      PRIV      OVER      SAFE      TECH
alpha  0.9223086 0.9525503 0.9403550 0.8971104 0.8642120 0.8918915 0.9186499
omega  0.9232185 0.9533841 0.9413611 0.8973915 0.8684702 0.8928921 0.9215426
omega2 0.9232185 0.9533841 0.9413611 0.8973915 0.8684702 0.8928921 0.9215426
omega3 0.9233399 0.9541262 0.9420148 0.8974292 0.8542191 0.8930025 0.9241917
avevar 0.7070732 0.8039492 0.8009060 0.6365261 0.6323012 0.6260871 0.7470105
            UNRE
alpha  0.9234861
omega  0.9246039
omega2 0.9246039
omega3 0.9268518
avevar 0.7106580
```

```r
semTools::discriminantValidity(fitCombined)
```

	lhs	op	rhs	est	ci.lower	ci.upper	Df	AIC	BIC	Chisq
1	COMP	~~	CONF	0.3647240	0.2833897	0.4460584	602	66280.85	66717.98	2804.836
2	COMP	~~	INSE	0.2755675	0.1902050	0.3609301	602	66367.26	66804.39	2891.246
3	COMP	~~	PRIV	0.3896698	0.3012986	0.4780410	602	66219.09	66656.22	2743.076
4	COMP	~~	OVER	0.6293570	0.5660105	0.6927035	602	65981.78	66418.90	2505.765
5	COMP	~~	SAFE	0.4053526	0.3265497	0.4841554	602	66208.72	66645.84	2732.703
6	COMP	~~	TECH	0.5813547	0.5145354	0.6481740	602	66041.75	66478.87	2565.734
7	COMP	~~	UNRE	0.5269954	0.4552961	0.5986946	602	66098.65	66535.78	2622.637
8	CONF	~~	INSE	0.2276620	0.1337610	0.3215631	602	66433.93	66871.05	2957.910
9	CONF	~~	PRIV	0.4171440	0.3363463	0.4979418	602	66211.47	66648.59	2735.453
10	CONF	~~	OVER	0.4568258	0.3760683	0.5375833	602	66171.22	66608.34	2695.202
11	CONF	~~	SAFE	0.3589030	0.2705774	0.4472286	602	66275.62	66712.74	2799.598
12	CONF	~~	TECH	0.3429523	0.2613736	0.4245310	602	66309.75	66746.87	2833.734
13	CONF	~~	UNRE	0.2610279	0.1738671	0.3481888	602	66391.80	66828.92	2915.782
14	INSE	~~	PRIV	0.4089965	0.3337388	0.4842542	602	66217.86	66654.98	2741.840
15	INSE	~~	OVER	0.3437673	0.2612792	0.4262554	602	66289.99	66727.12	2813.976
16	INSE	~~	SAFE	0.4464332	0.3584431	0.5344234	602	66179.90	66617.02	2703.880
17	INSE	~~	TECH	0.2209669	0.1387178	0.3032160	602	66426.51	66863.63	2950.494
18	INSE	~~	UNRE	0.2630446	0.1832939	0.3427953	602	66385.00	66822.12	2908.979
19	PRIV	~~	OVER	0.3828742	0.2964852	0.4692631	602	66218.04	66655.16	2742.019
20	PRIV	~~	SAFE	0.6531756	0.5882380	0.7181131	602	65937.73	66374.85	2461.713
21	PRIV	~~	TECH	0.3943046	0.3107197	0.4778894	602	66221.88	66659.00	2745.858
22	PRIV	~~	UNRE	0.3973955	0.3139693	0.4808217	602	66220.57	66657.69	2744.552
23	OVER	~~	SAFE	0.4172219	0.3320047	0.5024391	602	66186.55	66623.68	2710.536
24	OVER	~~	TECH	0.5677440	0.4988182	0.6366698	602	66050.12	66487.24	2574.101
25	OVER	~~	UNRE	0.6263317	0.5637919	0.6888714	602	65987.55	66424.67	2511.529
26	SAFE	~~	TECH	0.4027241	0.3206645	0.4847838	602	66215.52	66652.65	2739.506
27	SAFE	~~	UNRE	0.4029733	0.3189063	0.4870403	602	66216.10	66653.22	2740.080
28	TECH	~~	UNRE	0.7264283	0.6725664	0.7802902	602	65890.90	66328.02	2414.881

	Chisq diff	Df diff	Pr(>Chisq)
1	834.50332	1	1.700645e-183
2	491.90700	1	5.481158e-109
3	2471.31177	1	0.000000e+00
4	237.79901	1	1.187489e-53
5	186.68493	1	1.682568e-42
6	349.44231	1	5.605125e-78
7	484.84440	1	1.886353e-107
8	361.91070	1	1.080283e-80
9	1984.39850	1	0.000000e+00
10	573.62226	1	9.149173e-127
11	264.37851	1	1.906053e-59
12	780.26897	1	1.051984e-171
13	1398.25869	1	5.021330e-306

14	234.98622	1	4.875071e-53
15	404.56819	1	5.578425e-90
16	135.83969	1	2.163076e-31
17	412.56863	1	1.011595e-91
18	365.92928	1	1.440552e-81
19	17375.54384	1	0.000000e+00
20	45.49819	1	1.527802e-11
21	569.58856	1	6.899459e-126
22	935.79828	1	1.621333e-205
23	268.14004	1	2.885925e-60
24	585.69637	1	2.162790e-129
25	165.20025	1	8.271161e-38
26	244.59110	1	3.923558e-55
27	292.84580	1	1.192592e-65
28	118.75271	1	1.186345e-27
