# Supplementary material for: Measuring digital stress in Norway: translation and validation of the Digital Stressors Scale
Source: Front Psychol. 2024 Feb 9;15:1297194. doi: 10.3389/fpsyg.2024.1297194 (PMC10884271; doi:10.3389/fpsyg.2024.1297194)
Supplement: Supplementary file 3 [file Data_Sheet_3.PDF]

DSS

Sevic A., Foldnes N., Broennick K.

Table of contents

Data . . . 2

Results **2**

Import data . . . 2

Preliminary analyses . . . 3

Descriptives . . . 3

Missing data analysis . . . 5

Multivariate outliers . . . 6

Mardia's Multivariate Normality test . . . 7

Difference in scores on page 1 between those who completed and those who dropped out . . . 8

Correlation plot . . . 11

Confirmatory factor analysis **11**

Complexity factor . . . 11

Conflicts factor . . . 14

Insecurity factor . . . 17

Invasion of privacy factor . . . 19

Overload factor . . . 21

Safety factor . . . 24

Social Environment factor . . . 26

Technical support factor . . . 28

Usefulness factor . . . 31

Unreliability factor . . . 33

CFA for the whole model . . . 35

Second order reflective model . . . 37

Modification indices . . . 40

MI for bad fitting models . . . 40

MI for good fitting models for comparison . . . 42

MI for the rest . . . 42

CFA with MI 43

Reliability analyses **51**

Cronbach's Alpha, Alpha CIs and McDonald's Omega for subscales **51**

McDonald's Omega categorical for subscales 55

Exploratory Factor Analysis **58**

Data

For this analysis we've used the Norwegian data, in order to validate the Digital Stressors Scale on a Norwegian sample.

```
library(haven)
library(readxl)
library(readr)
library(psych)
library(lavaan)
library(stringr)
library(dplyr)
library(faoutlier)
```

Results

Import data

```
#import data
nor <- read_sav("onlydss.sav")
aus <- readxl::read_excel("final data set of DSS.xlsx")[, 2:51]
colnames(aus) <- str_remove_all(names(aus), "0")



#MISSING
all <- readRDS("complete.data")
names(all)[11:60] <- names(nor)
naniar::vis_miss(all)
```

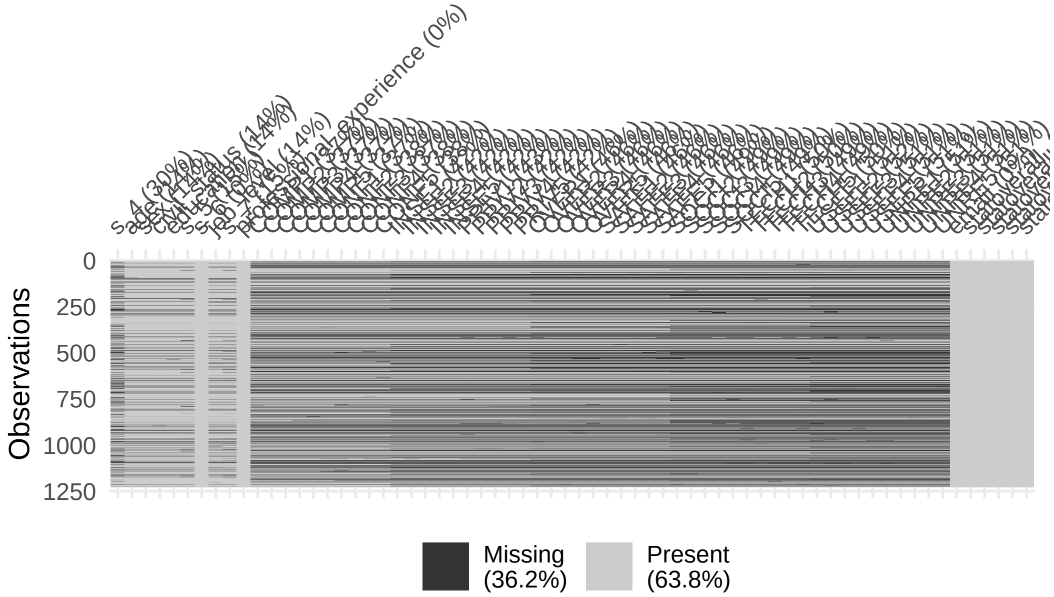

Preliminary analyses

Descriptives

```
#Descriptives
describe(nor)
```

	vars	n	mean	sd	median	trimmed	mad	min	max	range	skew	kurtosis	se
COMP1	1	560	3.26	1.77	3.0	3.15	1.48	1	7	6	0.31	-1.06	0.07
COMP2	2	560	3.52	1.96	3.0	3.44	2.97	1	7	6	0.25	-1.27	0.08
COMP3	3	560	3.34	1.80	3.0	3.25	1.48	1	7	6	0.34	-1.09	0.08
COMP4	4	560	4.35	1.99	5.0	4.43	2.97	1	7	6	-0.28	-1.23	0.08
COMP5	5	560	3.94	2.06	4.0	3.93	2.97	1	7	6	0.00	-1.37	0.09
CONF1	6	560	3.10	1.88	3.0	2.93	2.97	1	7	6	0.52	-0.95	0.08
CONF2	7	560	2.89	1.81	2.0	2.69	1.48	1	7	6	0.66	-0.75	0.08
CONF3	8	560	3.28	1.93	3.0	3.16	2.97	1	7	6	0.35	-1.21	0.08
CONF4	9	560	2.86	1.77	2.0	2.65	1.48	1	7	6	0.72	-0.64	0.07
CONF5	10	560	3.06	1.81	2.5	2.90	2.22	1	7	6	0.53	-0.91	0.08
INSE1	11	560	2.07	1.42	2.0	1.79	1.48	1	7	6	1.48	1.59	0.06
INSE2	12	560	1.85	1.38	1.0	1.53	0.00	1	7	6	1.91	3.04	0.06
INSE3	13	560	1.98	1.42	1.0	1.67	0.00	1	7	6	1.71	2.33	0.06

```
INSE4    14 560 1.74 1.35    1.0    1.39 0.00   1    7     6  2.10     3.69 0.06
INSE5    15 560 1.74 1.30    1.0    1.40 0.00   1    7     6  2.16     4.21 0.06
PRIV1    16 560 3.28 1.75    3.0    3.18 1.48   1    7     6  0.39    -0.99 0.07
PRIV2    17 560 3.58 1.79    4.0    3.53 2.97   1    7     6  0.18    -1.10 0.08
PRIV3    18 560 3.81 1.70    4.0    3.79 1.48   1    7     6  0.10    -0.91 0.07
PRIV4    19 560 3.96 1.67    4.0    3.95 1.48   1    7     6  0.02    -0.86 0.07
PRIV5    20 560 3.93 1.69    4.0    3.91 1.48   1    7     6  0.07    -0.88 0.07
OVER1    21 560 3.70 1.89    4.0    3.64 2.97   1    7     6  0.13    -1.14 0.08
OVER2    22 560 3.84 1.95    4.0    3.80 2.97   1    7     6  0.04    -1.24 0.08
OVER3    23 560 4.21 1.89    4.0    4.25 2.97   1    7     6 -0.19    -1.16 0.08
OVER4    24 560 3.20 1.73    3.0    3.08 1.48   1    7     6  0.43    -0.82 0.07
OVER5    25 560 3.95 1.86    4.0    3.94 2.97   1    7     6 -0.02    -1.12 0.08
SAFE1    26 560 2.67 1.55    2.0    2.48 1.48   1    7     6  0.94     0.08 0.07
SAFE2    27 560 2.71 1.58    2.0    2.51 1.48   1    7     6  0.95     0.06 0.07
SAFE3    28 560 2.86 1.69    2.0    2.67 1.48   1    7     6  0.79    -0.33 0.07
SAFE4    29 560 3.00 1.75    2.5    2.82 2.22   1    7     6  0.68    -0.58 0.07
SAFE5    30 560 2.78 1.64    2.0    2.60 1.48   1    7     6  0.81    -0.27 0.07
SOCI1    31 560 3.08 1.79    3.0    2.92 1.48   1    7     6  0.57    -0.77 0.08
SOCI2    32 560 4.02 1.94    4.0    4.03 2.97   1    7     6 -0.04    -1.22 0.08
SOCI3    33 560 2.98 1.73    2.0    2.79 1.48   1    7     6  0.72    -0.52 0.07
SOCI4    34 560 4.07 1.87    4.0    4.08 2.97   1    7     6 -0.11    -1.14 0.08
SOCI5    35 560 3.26 1.73    3.0    3.15 1.48   1    7     6  0.41    -0.91 0.07
TECH1    36 560 3.01 1.74    2.0    2.84 1.48   1    7     6  0.65    -0.65 0.07
TECH2    37 560 3.59 1.95    3.0    3.50 2.97   1    7     6  0.25    -1.21 0.08
TECH3    38 560 3.86 1.99    4.0    3.83 2.97   1    7     6  0.06    -1.30 0.08
TECH4    39 560 3.85 1.97    4.0    3.81 2.97   1    7     6  0.03    -1.28 0.08
TECH5    40 560 3.59 1.97    3.0    3.51 2.97   1    7     6  0.20    -1.28 0.08
USEF1    41 560 3.96 1.88    4.0    3.95 2.97   1    7     6  0.05    -1.13 0.08
USEF2    42 560 3.62 1.84    4.0    3.58 2.97   1    7     6  0.17    -1.18 0.08
USEF3    43 560 4.31 1.81    5.0    4.35 1.48   1    7     6 -0.19    -1.03 0.08
USEF4    44 560 4.44 1.95    5.0    4.54 2.97   1    7     6 -0.31    -1.15 0.08
USEF5    45 560 3.31 1.84    3.0    3.17 1.48   1    7     6  0.48    -0.84 0.08
UNRE1    46 560 3.96 1.86    4.0    3.93 2.97   1    7     6  0.10    -1.11 0.08
UNRE2    47 560 3.92 1.90    4.0    3.90 2.97   1    7     6  0.05    -1.18 0.08
UNRE3    48 560 3.56 1.82    3.0    3.48 1.48   1    7     6  0.28    -1.01 0.08
UNRE4    49 560 3.46 1.85    3.0    3.36 1.48   1    7     6  0.38    -1.04 0.08
UNRE5    50 560 4.03 2.02    4.0    4.04 2.97   1    7     6 -0.03    -1.32 0.09
```

```r
#Filter rows with complete responses in columns 11 to 60
complete_responses <- all[complete.cases(all[, 11:60]), ]

#Select relevant columns from the filtered dataset
```

```r
tab <- select(complete_responses, c("age", "sex", "education", "civil_status"))

#Filter for valid age and calculate statistics
validage <- tab[tab$age < 1000 & !is.na(tab$age), "age"]
age_stats <- round(c(mean = mean(validage), sd = sd(validage)), 1)
print(age_stats)
```

```
mean   sd
42.7 10.9
```

```r
#Proportional tables for sex, education, and civil status
prop.table(table(tab$sex))
```

```
          1           2           3
0.783154122 0.213261649 0.003584229
```

```r
prop.table(table(tab$education))
```

```
          1           2           3           4
0.008960573 0.279569892 0.399641577 0.311827957
```

```r
prop.table(table(tab$civil_status))
```

```
          1           2           3           4           5           6
0.211469534 0.308243728 0.378136201 0.012544803 0.082437276 0.007168459
```

Missing data analysis

```r
naniar::vis_miss(nor)
```

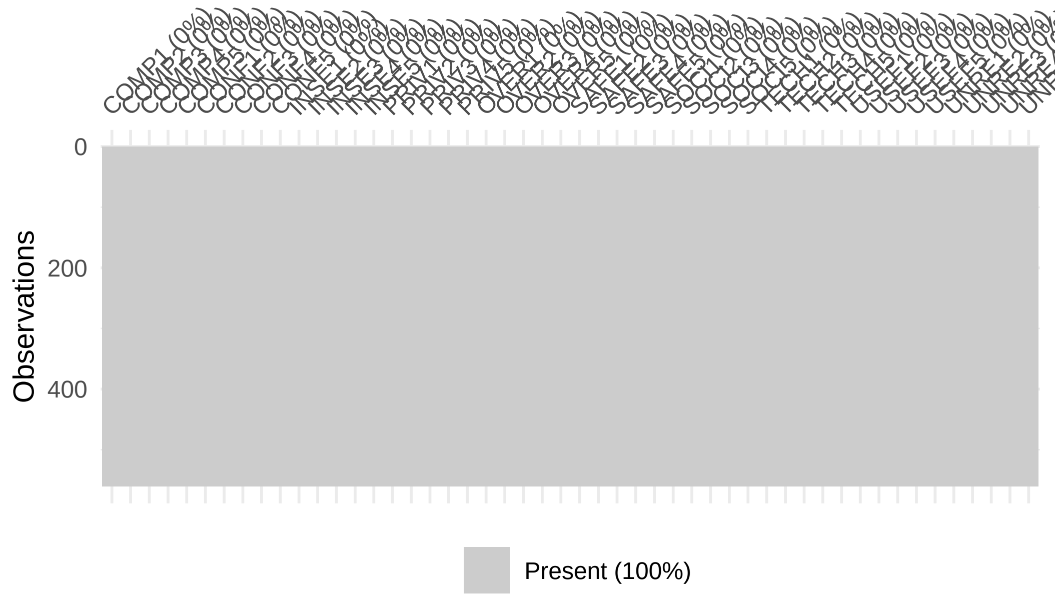

Multivariate outliers

```
out= robustMD(nor, method = "mcd") #Outlier detection performed on complete cases.
print(out)
```

```
         mah p  sig
117 506.7504 0 ****
27  501.8619 0 ****
4   485.4109 0 ****
63  372.0737 0 ****
149 329.2038 0 ****
121 313.2997 0 ****
493 312.2685 0 ****
108 294.2188 0 ****
46  293.3693 0 ****
239 290.6495 0 ****
```

```
plot(out)
```

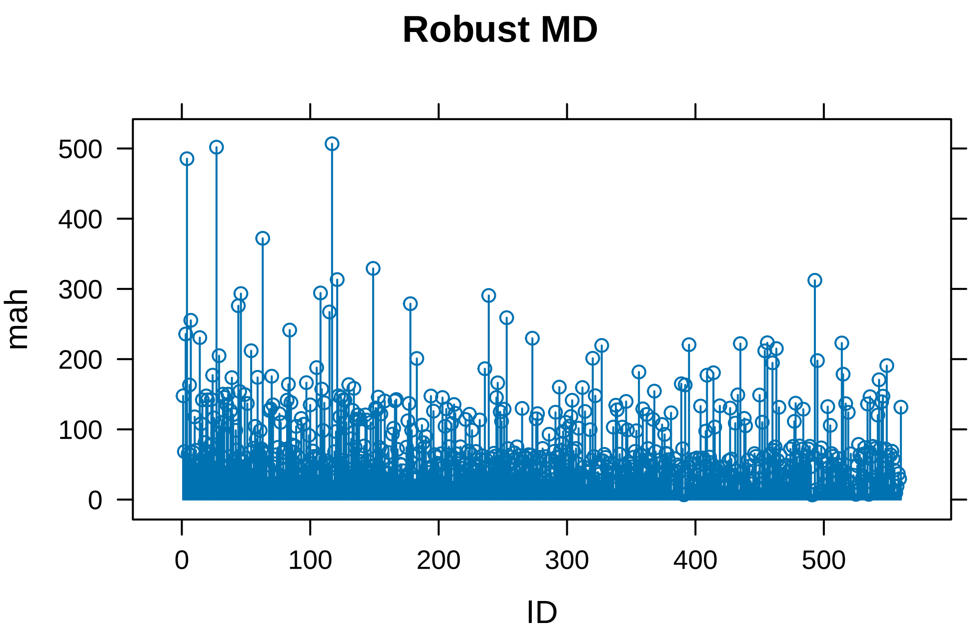

Mardia's Multivariate Normality test

```
mardia(nor, na.rm = TRUE, plot = TRUE)
```

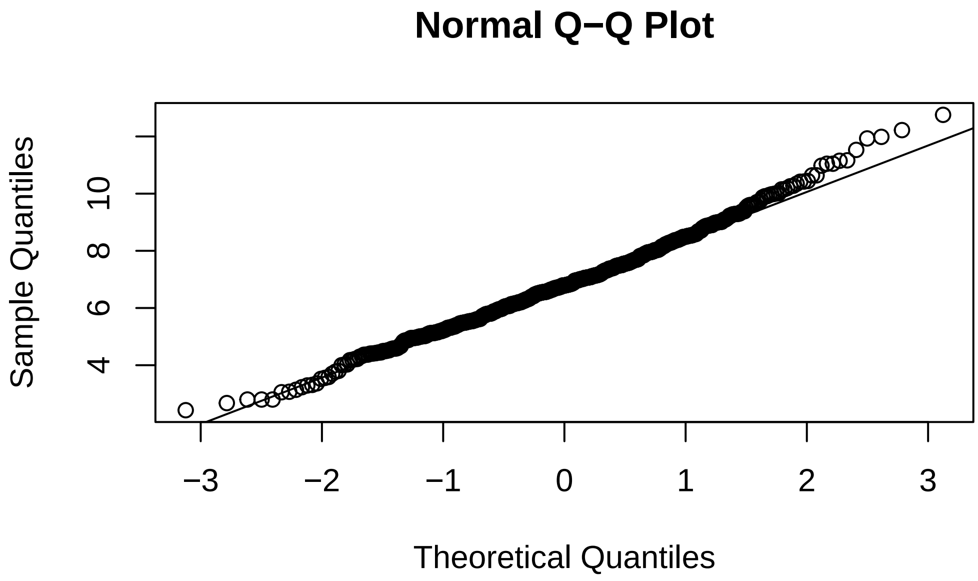


```
Call: mardia(x = nor, na.rm = TRUE, plot = TRUE)

Mardia tests of multivariate skew and kurtosis
Use describe(x) the to get univariate tests
n.obs = 560   num.vars =  50
b1p =  423.6   skew =  39536.45  with probability  <=  0
 small sample skew =  39756.58  with probability <=  0
b2p =  3080.03   kurtosis =  78.76  with probability <=  0
```

Difference in scores on page 1 between those who completed and those who dropped out

```
#page 1
all$page1_missing <- rowSums(is.na(dplyr::select(all, contains(c("COMP", "CONF"), ignore.c
table(all$page1_missing)
```

```
  0   1   4   5   7  10
757  10   3   1   1 456
```

```
all <- all[all$page1_missing < 10, ]
naniar::vis_miss(all)
```

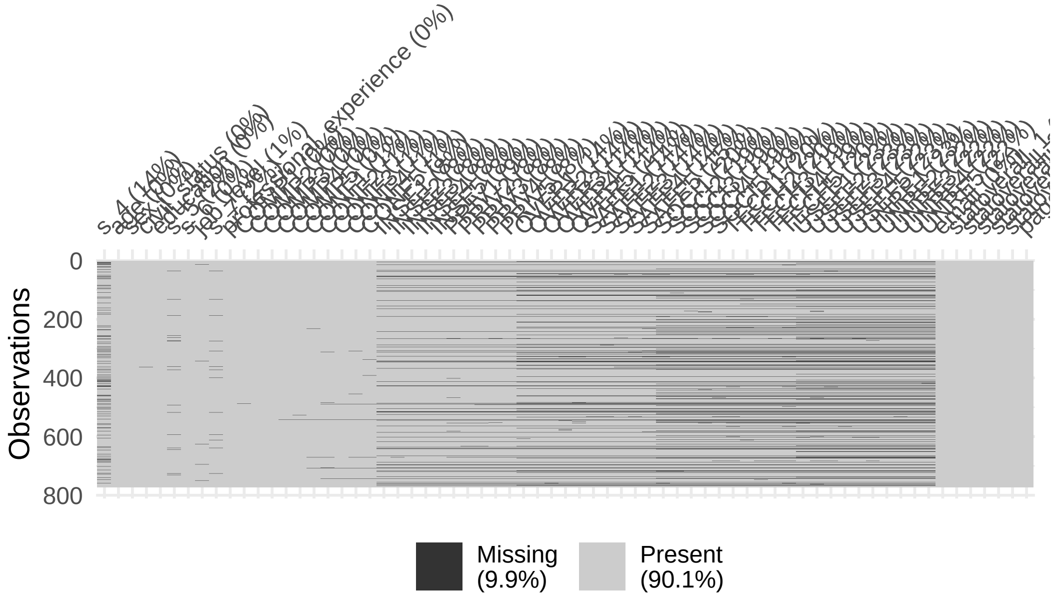


```
round(100*mean(is.na(dplyr::select(all, contains(c("COMP", "CONF"), ignore.case = F)))), 1
```

```
[1] 0.4
```

```
round(100*mean(is.na(dplyr::select(all, contains(c("INSE", "PRIV"), ignore.case = F)))), 1
```

```
[1] 8
```

```
round(100*mean(is.na(dplyr::select(all, contains(c("OVER", "SAFE"), ignore.case = F)))), 1
```

```
[1] 14.1
```

```
round(100*mean(is.na(dplyr::select(all, contains(c("SOCI", "TECH"), ignore.case = F)))), 1
```

```
[1] 19.5
```

```r
round(100*mean(is.na(dplyr::select(all, contains(c("USEF", "UNRE"), ignore.case = F)))), 1
```

```
[1] 22.6
```

```r
## look at stress in ten first items, in two groups: those who completed the last items vs
comp_idx <- complete.cases(dplyr::select(all, contains(c("COMP", "CONF"))))
tmp <- all[comp_idx, ]
comp_last <-  complete.cases(dplyr::select(tmp, contains(c("UNRE"))))

stress.df <- data.frame(stress=rowMeans(dplyr::select(all, contains(c("COMP", "CONF"))) %>
                        finished=comp_last)

stress.df %>% group_by(finished) %>% summarise(mean(stress), n())
```

```
# A tibble: 2 x 3
  finished `mean(stress)` `n()`
  <lgl>             <dbl> <int>
1 FALSE              3.53   167
2 TRUE               3.35   590
```

```r
t.test(stress.df$stress~stress.df$finished)
```

```
    Welch Two Sample t-test

data:  stress.df$stress by stress.df$finished
t = 1.3981, df = 252.87, p-value = 0.1633
alternative hypothesis: true difference in means between group FALSE and group TRUE is not e
95 percent confidence interval:
 -0.07296987  0.43011592
sample estimates:
mean in group FALSE  mean in group TRUE
           3.528743            3.350169
```

Correlation plot

```
all <- dplyr::select(nor, contains(c("COMP", "CONF","INSE", "PRIV", "OVER", "SAFE",
                                     "SOCI", "TECH","USEF", "UNRE" ), ignore.case = F))
corr <- cor(nor, use="pairwise.complete.obs")
```

Confirmatory factor analysis

Complexity factor

```
m1 <- 'f1=~COMP1 + COMP2 + COMP3 + COMP4 + COMP5'
complexityfactor <- lavaan::cfa(m1, data=nor, estimator = "MLR")
summary(complexityfactor,fit.measures=TRUE,standardized=TRUE)
```

```
lavaan 0.6.17 ended normally after 20 iterations

  Estimator                                         ML
  Optimization method                           NLMINB
  Number of model parameters                        10

  Number of observations                           560

Model Test User Model:
                                              Standard      Scaled
  Test Statistic                               262.992     194.917
  Degrees of freedom                                 5           5
  P-value (Chi-square)                           0.000       0.000
  Scaling correction factor                                  1.349
    Yuan-Bentler correction (Mplus variant)

Model Test Baseline Model:

  Test statistic                              2260.798    1401.591
  Degrees of freedom                                10          10
  P-value                                        0.000       0.000
  Scaling correction factor                                  1.613

User Model versus Baseline Model:
```

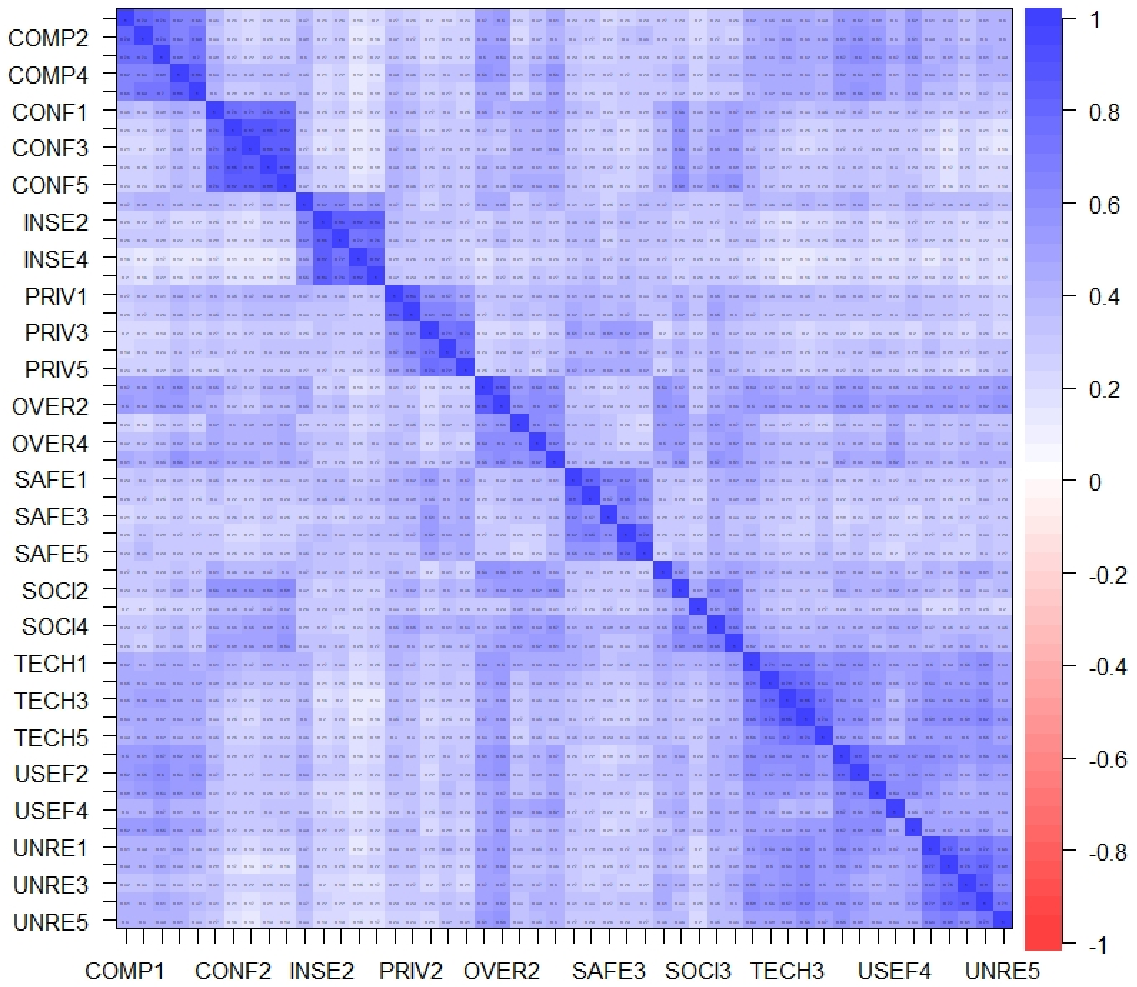

Figure 1: Correlation plot

```
  Comparative Fit Index (CFI)                    0.885       0.864
  Tucker-Lewis Index (TLI)                       0.771       0.727

  Robust Comparative Fit Index (CFI)                         0.886
  Robust Tucker-Lewis Index (TLI)                            0.772

Loglikelihood and Information Criteria:

  Loglikelihood user model (H0)              -4785.555   -4785.555
  Scaling correction factor                                  1.139
      for the MLR correction
  Loglikelihood unrestricted model (H1)      -4654.059   -4654.059
  Scaling correction factor                                  1.209
      for the MLR correction

  Akaike (AIC)                                9591.110    9591.110
  Bayesian (BIC)                              9634.389    9634.389
  Sample-size adjusted Bayesian (SABIC)       9602.645    9602.645

Root Mean Square Error of Approximation:

  RMSEA                                          0.304       0.260
  90 Percent confidence interval - lower         0.273       0.234
  90 Percent confidence interval - upper         0.335       0.288
  P-value H_0: RMSEA <= 0.050                    0.000       0.000
  P-value H_0: RMSEA >= 0.080                    1.000       1.000

  Robust RMSEA                                               0.303
  90 Percent confidence interval - lower                     0.267
  90 Percent confidence interval - upper                     0.340
  P-value H_0: Robust RMSEA <= 0.050                         0.000
  P-value H_0: Robust RMSEA >= 0.080                         1.000

Standardized Root Mean Square Residual:

  SRMR                                           0.055       0.055

Parameter Estimates:

  Standard errors                             Sandwich
  Information bread                           Observed
  Observed information based on                Hessian
```

```
Latent Variables:
                   Estimate  Std.Err  z-value  P(>|z|)   Std.lv  Std.all
  f1 =~
    COMP1             1.000                               1.467    0.828
    COMP2             1.126    0.031   35.966    0.000    1.651    0.844
    COMP3             1.045    0.036   28.679    0.000    1.533    0.852
    COMP4             1.116    0.070   15.917    0.000    1.637    0.826
    COMP5             1.194    0.069   17.404    0.000    1.751    0.852

Variances:
                   Estimate  Std.Err  z-value  P(>|z|)   Std.lv  Std.all
   .COMP1             0.983    0.128    7.689    0.000    0.983    0.314
   .COMP2             1.104    0.132    8.334    0.000    1.104    0.288
   .COMP3             0.885    0.090    9.859    0.000    0.885    0.273
   .COMP4             1.252    0.165    7.597    0.000    1.252    0.318
   .COMP5             1.155    0.153    7.553    0.000    1.155    0.274
    f1                2.152    0.175   12.289    0.000    1.000    1.000
```

```
fitmeasures(complexityfactor, c("cfi", "tli", "rmsea","srmr", "chisq", "df"))
```

```
    cfi     tli   rmsea    srmr   chisq      df
  0.885   0.771   0.304   0.055 262.992   5.000
```

Conflicts factor

```
m2 <- 'f2=~CONF1 + CONF2 + CONF3 + CONF4 + CONF5'
conflictsfactor <- lavaan::cfa(m2, data=nor, estimator = "MLR")
summary(conflictsfactor,fit.measures=TRUE,standardized=TRUE)
```

```
lavaan 0.6.17 ended normally after 26 iterations

  Estimator                                         ML
  Optimization method                           NLMINB
  Number of model parameters                        10

  Number of observations                           560

Model Test User Model:
                                              Standard      Scaled
  Test Statistic                                61.050      29.205
```

```
  Degrees of freedom                                 5           5
  P-value (Chi-square)                           0.000       0.000
  Scaling correction factor                                  2.090
    Yuan-Bentler correction (Mplus variant)

Model Test Baseline Model:

  Test statistic                              3051.541    1242.378
  Degrees of freedom                                10          10
  P-value                                        0.000       0.000
  Scaling correction factor                                  2.456

User Model versus Baseline Model:

  Comparative Fit Index (CFI)                    0.982       0.980
  Tucker-Lewis Index (TLI)                       0.963       0.961

  Robust Comparative Fit Index (CFI)                         0.983
  Robust Tucker-Lewis Index (TLI)                            0.967

Loglikelihood and Information Criteria:

  Loglikelihood user model (H0)              -4180.032   -4180.032
  Scaling correction factor                                  1.689
      for the MLR correction
  Loglikelihood unrestricted model (H1)      -4149.507   -4149.507
  Scaling correction factor                                  1.823
      for the MLR correction

  Akaike (AIC)                                8380.063    8380.063
  Bayesian (BIC)                              8423.343    8423.343
  Sample-size adjusted Bayesian (SABIC)       8391.598    8391.598

Root Mean Square Error of Approximation:

  RMSEA                                          0.141       0.093
  90 Percent confidence interval - lower         0.111       0.071
  90 Percent confidence interval - upper         0.174       0.116
  P-value H_0: RMSEA <= 0.050                    0.000       0.001
  P-value H_0: RMSEA >= 0.080                    0.999       0.844

  Robust RMSEA                                               0.134
  90 Percent confidence interval - lower                     0.090
```

```
  90 Percent confidence interval - upper                         0.184
  P-value H_0: Robust RMSEA <= 0.050                             0.002
  P-value H_0: Robust RMSEA >= 0.080                             0.976

Standardized Root Mean Square Residual:

  SRMR                                           0.014       0.014

Parameter Estimates:

  Standard errors                             Sandwich
  Information bread                           Observed
  Observed information based on                Hessian

Latent Variables:
                   Estimate  Std.Err  z-value  P(>|z|)   Std.lv  Std.all
  f2 =~
    CONF1             1.000                               1.494    0.796
    CONF2             1.113    0.037   30.316    0.000    1.663    0.918
    CONF3             1.180    0.042   28.399    0.000    1.762    0.914
    CONF4             1.101    0.038   29.107    0.000    1.645    0.933
    CONF5             1.114    0.038   29.145    0.000    1.665    0.920

Variances:
                   Estimate  Std.Err  z-value  P(>|z|)   Std.lv  Std.all
   .CONF1             1.288    0.104   12.367    0.000    1.288    0.366
   .CONF2             0.518    0.061    8.553    0.000    0.518    0.158
   .CONF3             0.609    0.092    6.620    0.000    0.609    0.164
   .CONF4             0.406    0.049    8.332    0.000    0.406    0.130
   .CONF5             0.501    0.078    6.417    0.000    0.501    0.153
    f2                2.231    0.174   12.788    0.000    1.000    1.000
```

```r
fitmeasures(conflictsfactor, c("cfi", "tli", "rmsea","srmr", "chisq", "df"))
```

```
   cfi    tli  rmsea   srmr  chisq     df 
 0.982  0.963  0.141  0.014 61.050  5.000 
```

Insecurity factor

```
m3<- 'f3=~INSE1 + INSE2 + INSE3 + INSE4 + INSE5'
insecurityfactor <- lavaan::cfa(m3, data=nor, estimator = "MLR")
summary(insecurityfactor,fit.measures=TRUE,standardized=TRUE)
```

```
lavaan 0.6.17 ended normally after 24 iterations

  Estimator                                         ML
  Optimization method                           NLMINB
  Number of model parameters                        10

  Number of observations                           560

Model Test User Model:
                                              Standard      Scaled
  Test Statistic                               100.875      32.435
  Degrees of freedom                                 5           5
  P-value (Chi-square)                           0.000       0.000
  Scaling correction factor                                  3.110
    Yuan-Bentler correction (Mplus variant)

Model Test Baseline Model:

  Test statistic                              2437.173     638.046
  Degrees of freedom                                10          10
  P-value                                        0.000       0.000
  Scaling correction factor                                  3.820

User Model versus Baseline Model:

  Comparative Fit Index (CFI)                    0.960       0.956
  Tucker-Lewis Index (TLI)                       0.921       0.913

  Robust Comparative Fit Index (CFI)                         0.964
  Robust Tucker-Lewis Index (TLI)                            0.929

Loglikelihood and Information Criteria:

  Loglikelihood user model (H0)              -3694.425   -3694.425
  Scaling correction factor                                  3.513
```

```
    for the MLR correction
  Loglikelihood unrestricted model (H1)      -3643.987   -3643.987
  Scaling correction factor                                  3.379
    for the MLR correction

  Akaike (AIC)                                7408.849    7408.849
  Bayesian (BIC)                              7452.129    7452.129
  Sample-size adjusted Bayesian (SABIC)       7420.384    7420.384

Root Mean Square Error of Approximation:

  RMSEA                                          0.185       0.099
  90 Percent confidence interval - lower         0.155       0.081
  90 Percent confidence interval - upper         0.217       0.118
  P-value H_0: RMSEA <= 0.050                    0.000       0.000
  P-value H_0: RMSEA >= 0.080                    1.000       0.959

  Robust RMSEA                                               0.175
  90 Percent confidence interval - lower                     0.120
  90 Percent confidence interval - upper                     0.234
  P-value H_0: Robust RMSEA <= 0.050                         0.000
  P-value H_0: Robust RMSEA >= 0.080                         0.997

Standardized Root Mean Square Residual:

  SRMR                                           0.035       0.035

Parameter Estimates:

  Standard errors                             Sandwich
  Information bread                           Observed
  Observed information based on                Hessian

Latent Variables:
                   Estimate  Std.Err  z-value  P(>|z|)   Std.lv  Std.all
  f3 =~
    INSE1             1.000                               0.903    0.635
    INSE2             1.454    0.103   14.134    0.000    1.313    0.951
    INSE3             1.378    0.091   15.076    0.000    1.245    0.876
    INSE4             1.287    0.120   10.763    0.000    1.162    0.862
    INSE5             1.278    0.105   12.194    0.000    1.154    0.885

Variances:
```

```
                 Estimate  Std.Err  z-value  P(>|z|)   Std.lv  Std.all
   .INSE1           1.205    0.121    9.924    0.000    1.205    0.596
   .INSE2           0.181    0.033    5.406    0.000    0.181    0.095
   .INSE3           0.472    0.088    5.351    0.000    0.472    0.233
   .INSE4           0.466    0.074    6.293    0.000    0.466    0.257
   .INSE5           0.367    0.075    4.870    0.000    0.367    0.216
    f3              0.815    0.134    6.089    0.000    1.000    1.000
```

```
fitmeasures(insecurityfactor, c("cfi", "tli", "rmsea","srmr", "chisq", "df"))
```

```
    cfi     tli   rmsea    srmr   chisq      df 
  0.960   0.921   0.185   0.035 100.875   5.000 
```

Invasion of privacy factor

```
m4<- 'f4=~PRIV1 + PRIV2 + PRIV3 + PRIV4 + PRIV5'
privacyfactor<- lavaan::cfa(m4, data=nor, estimator = "MLR")
summary(privacyfactor,fit.measures=TRUE,standardized=TRUE)
```

```
lavaan 0.6.17 ended normally after 22 iterations

  Estimator                                         ML
  Optimization method                           NLMINB
  Number of model parameters                        10

  Number of observations                           560

Model Test User Model:
                                              Standard      Scaled
  Test Statistic                               344.343     248.445
  Degrees of freedom                                 5           5
  P-value (Chi-square)                           0.000       0.000
  Scaling correction factor                                  1.386
    Yuan-Bentler correction (Mplus variant)

Model Test Baseline Model:

  Test statistic                              1922.501    1113.588
  Degrees of freedom                                10          10
```

```
  P-value                                        0.000       0.000
  Scaling correction factor                                  1.726

User Model versus Baseline Model:

  Comparative Fit Index (CFI)                    0.823       0.779
  Tucker-Lewis Index (TLI)                       0.645       0.559

  Robust Comparative Fit Index (CFI)                         0.823
  Robust Tucker-Lewis Index (TLI)                            0.646

Loglikelihood and Information Criteria:

  Loglikelihood user model (H0)              -4694.371   -4694.371
  Scaling correction factor                                  1.298
      for the MLR correction
  Loglikelihood unrestricted model (H1)      -4522.199   -4522.199
  Scaling correction factor                                  1.327
      for the MLR correction

  Akaike (AIC)                                9408.741    9408.741
  Bayesian (BIC)                              9452.020    9452.020
  Sample-size adjusted Bayesian (SABIC)       9420.275    9420.275

Root Mean Square Error of Approximation:

  RMSEA                                          0.348       0.295
  90 Percent confidence interval - lower         0.317       0.269
  90 Percent confidence interval - upper         0.380       0.322
  P-value H_0: RMSEA <= 0.050                    0.000       0.000
  P-value H_0: RMSEA >= 0.080                    1.000       1.000

  Robust RMSEA                                               0.347
  90 Percent confidence interval - lower                     0.311
  90 Percent confidence interval - upper                     0.385
  P-value H_0: Robust RMSEA <= 0.050                         0.000
  P-value H_0: Robust RMSEA >= 0.080                         1.000

Standardized Root Mean Square Residual:

  SRMR                                           0.080       0.080

Parameter Estimates:
```

```
  Standard errors                                 Sandwich
  Information bread                               Observed
  Observed information based on                    Hessian

Latent Variables:
                   Estimate  Std.Err  z-value  P(>|z|)   Std.lv  Std.all
  f4 =~
    PRIV1             1.000                               1.284    0.735
    PRIV2             1.106    0.031   35.511    0.000    1.420    0.795
    PRIV3             1.093    0.089   12.342    0.000    1.403    0.828
    PRIV4             1.092    0.086   12.628    0.000    1.402    0.842
    PRIV5             1.033    0.095   10.835    0.000    1.326    0.787

Variances:
                   Estimate  Std.Err  z-value  P(>|z|)   Std.lv  Std.all
   .PRIV1             1.405    0.178    7.909    0.000    1.405    0.460
   .PRIV2             1.175    0.165    7.100    0.000    1.175    0.368
   .PRIV3             0.901    0.116    7.759    0.000    0.901    0.314
   .PRIV4             0.805    0.116    6.956    0.000    0.805    0.291
   .PRIV5             1.083    0.139    7.814    0.000    1.083    0.381
    f4                1.649    0.211    7.798    0.000    1.000    1.000
```

```
fitmeasures(privacyfactor, c("cfi", "tli", "rmsea","srmr", "chisq", "df"))
```

```
    cfi     tli   rmsea    srmr   chisq      df 
  0.823   0.645   0.348   0.080 344.343   5.000 
```

Overload factor

```
m5<- 'f5=~OVER1 + OVER2 + OVER3 + OVER4+ OVER5'
overloadfactor<- lavaan::cfa(m5, data=nor, estimator = "MLR")
summary(overloadfactor,fit.measures=TRUE,standardized=TRUE)
```

```
lavaan 0.6.17 ended normally after 25 iterations

  Estimator                                         ML
  Optimization method                           NLMINB
  Number of model parameters                        10
```

```
  Number of observations                           560

Model Test User Model:
                                              Standard      Scaled
  Test Statistic                               153.619     125.245
  Degrees of freedom                                 5           5
  P-value (Chi-square)                           0.000       0.000
  Scaling correction factor                                  1.227
    Yuan-Bentler correction (Mplus variant)

Model Test Baseline Model:

  Test statistic                              1616.602     961.410
  Degrees of freedom                                10          10
  P-value                                        0.000       0.000
  Scaling correction factor                                  1.681

User Model versus Baseline Model:

  Comparative Fit Index (CFI)                    0.907       0.874
  Tucker-Lewis Index (TLI)                       0.815       0.747

  Robust Comparative Fit Index (CFI)                         0.908
  Robust Tucker-Lewis Index (TLI)                            0.816

Loglikelihood and Information Criteria:

  Loglikelihood user model (H0)              -4977.577   -4977.577
  Scaling correction factor                                  1.296
      for the MLR correction
  Loglikelihood unrestricted model (H1)      -4900.767   -4900.767
  Scaling correction factor                                  1.273
      for the MLR correction

  Akaike (AIC)                                9975.153    9975.153
  Bayesian (BIC)                             10018.433   10018.433
  Sample-size adjusted Bayesian (SABIC)       9986.688    9986.688

Root Mean Square Error of Approximation:

  RMSEA                                          0.230       0.207
  90 Percent confidence interval - lower         0.200       0.180
  90 Percent confidence interval - upper         0.262       0.236
```

```
  P-value H_0: RMSEA <= 0.050                    0.000       0.000
  P-value H_0: RMSEA >= 0.080                    1.000       1.000

  Robust RMSEA                                               0.230
  90 Percent confidence interval - lower                     0.196
  90 Percent confidence interval - upper                     0.265
  P-value H_0: Robust RMSEA <= 0.050                         0.000
  P-value H_0: Robust RMSEA >= 0.080                         1.000

Standardized Root Mean Square Residual:

  SRMR                                           0.072       0.072

Parameter Estimates:

  Standard errors                             Sandwich
  Information bread                           Observed
  Observed information based on                Hessian

Latent Variables:
                   Estimate  Std.Err  z-value  P(>|z|)   Std.lv  Std.all
  f5 =~
    OVER1             1.000                               1.694    0.896
    OVER2             1.046    0.026   40.133    0.000    1.772    0.911
    OVER3             0.695    0.051   13.565    0.000    1.178    0.625
    OVER4             0.709    0.046   15.307    0.000    1.202    0.696
    OVER5             0.721    0.048   15.070    0.000    1.222    0.659

Variances:
                   Estimate  Std.Err  z-value  P(>|z|)   Std.lv  Std.all
   .OVER1             0.708    0.107    6.587    0.000    0.708    0.198
   .OVER2             0.641    0.123    5.195    0.000    0.641    0.170
   .OVER3             2.167    0.155   13.975    0.000    2.167    0.610
   .OVER4             1.538    0.132   11.607    0.000    1.538    0.516
   .OVER5             1.943    0.144   13.475    0.000    1.943    0.565
    f5                2.870    0.178   16.161    0.000    1.000    1.000
```

```
fitmeasures(overloadfactor, c("cfi", "tli", "rmsea","srmr", "chisq", "df"))
```

```
    cfi     tli   rmsea    srmr   chisq      df 
  0.907   0.815   0.230   0.072 153.619   5.000 
```

Safety factor

```r
m6<- 'f6=~SAFE1 + SAFE2 + SAFE3 + SAFE4 + SAFE5'
safetyfactor<- lavaan::cfa(m6, data=nor, estimator = "MLR")
summary(safetyfactor,fit.measures=TRUE,standardized=TRUE)
```

```
lavaan 0.6.17 ended normally after 24 iterations

  Estimator                                         ML
  Optimization method                           NLMINB
  Number of model parameters                        10

  Number of observations                           560

Model Test User Model:
                                              Standard      Scaled
  Test Statistic                               278.228     163.252
  Degrees of freedom                                 5           5
  P-value (Chi-square)                           0.000       0.000
  Scaling correction factor                                  1.704
    Yuan-Bentler correction (Mplus variant)

Model Test Baseline Model:

  Test statistic                              1819.655     723.356
  Degrees of freedom                                10          10
  P-value                                        0.000       0.000
  Scaling correction factor                                  2.516

User Model versus Baseline Model:

  Comparative Fit Index (CFI)                    0.849       0.778
  Tucker-Lewis Index (TLI)                       0.698       0.556

  Robust Comparative Fit Index (CFI)                         0.850
  Robust Tucker-Lewis Index (TLI)                            0.699

Loglikelihood and Information Criteria:

  Loglikelihood user model (H0)              -4583.891   -4583.891
  Scaling correction factor                                  2.114
```

```
      for the MLR correction
  Loglikelihood unrestricted model (H1)      -4444.777   -4444.777
  Scaling correction factor                                  1.977
      for the MLR correction

  Akaike (AIC)                                9187.782    9187.782
  Bayesian (BIC)                              9231.062    9231.062
  Sample-size adjusted Bayesian (SABIC)       9199.317    9199.317

Root Mean Square Error of Approximation:

  RMSEA                                          0.312       0.238
  90 Percent confidence interval - lower         0.282       0.214
  90 Percent confidence interval - upper         0.344       0.262
  P-value H_0: RMSEA <= 0.050                    0.000       0.000
  P-value H_0: RMSEA >= 0.080                    1.000       1.000

  Robust RMSEA                                               0.310
  90 Percent confidence interval - lower                     0.270
  90 Percent confidence interval - upper                     0.352
  P-value H_0: Robust RMSEA <= 0.050                         0.000
  P-value H_0: Robust RMSEA >= 0.080                         1.000

Standardized Root Mean Square Residual:

  SRMR                                           0.065       0.065

Parameter Estimates:

  Standard errors                             Sandwich
  Information bread                           Observed
  Observed information based on                Hessian

Latent Variables:
                   Estimate  Std.Err  z-value  P(>|z|)   Std.lv  Std.all
  f6 =~
    SAFE1             1.000                               1.297    0.839
    SAFE2             1.017    0.034   30.239    0.000    1.319    0.837
    SAFE3             0.921    0.081   11.432    0.000    1.195    0.709
    SAFE4             1.114    0.144    7.717    0.000    1.445    0.826
    SAFE5             0.937    0.138    6.776    0.000    1.216    0.741

Variances:
```

```
                 Estimate  Std.Err  z-value  P(>|z|)   Std.lv  Std.all
   .SAFE1           0.709    0.205    3.453    0.001    0.709    0.296
   .SAFE2           0.745    0.204    3.652    0.000    0.745    0.300
   .SAFE3           1.410    0.136   10.405    0.000    1.410    0.497
   .SAFE4           0.969    0.261    3.706    0.000    0.969    0.317
   .SAFE5           1.217    0.244    4.986    0.000    1.217    0.452
    f6              1.682    0.255    6.599    0.000    1.000    1.000
```

```
fitmeasures(safetyfactor, c("cfi", "tli", "rmsea","srmr", "chisq", "df"))
```

```
    cfi     tli   rmsea    srmr   chisq      df 
  0.849   0.698   0.312   0.065 278.228   5.000 
```

Social Environment factor

```
m7<- 'f7=~SOCI1 + SOCI2 + SOCI3 + SOCI4 + SOCI5'
sefactor<- lavaan::cfa(m7, data=nor, estimator = "MLR")
summary(sefactor,fit.measures=TRUE,standardized=TRUE)
```

```
lavaan 0.6.17 ended normally after 24 iterations

  Estimator                                         ML
  Optimization method                           NLMINB
  Number of model parameters                        10

  Number of observations                           560

Model Test User Model:
                                              Standard      Scaled
  Test Statistic                                61.410      51.278
  Degrees of freedom                                 5           5
  P-value (Chi-square)                           0.000       0.000
  Scaling correction factor                                  1.198
    Yuan-Bentler correction (Mplus variant)                       

Model Test Baseline Model:

  Test statistic                              1157.594     836.115
  Degrees of freedom                                10          10
  P-value                                        0.000       0.000
```

```
  Scaling correction factor                                  1.384

User Model versus Baseline Model:

  Comparative Fit Index (CFI)                    0.951       0.944
  Tucker-Lewis Index (TLI)                       0.902       0.888

  Robust Comparative Fit Index (CFI)                         0.952
  Robust Tucker-Lewis Index (TLI)                            0.903

Loglikelihood and Information Criteria:

  Loglikelihood user model (H0)              -5086.114   -5086.114
  Scaling correction factor                                  1.060
      for the MLR correction
  Loglikelihood unrestricted model (H1)      -5055.409   -5055.409
  Scaling correction factor                                  1.106
      for the MLR correction

  Akaike (AIC)                               10192.229   10192.229
  Bayesian (BIC)                             10235.508   10235.508
  Sample-size adjusted Bayesian (SABIC)      10203.763   10203.763

Root Mean Square Error of Approximation:

  RMSEA                                          0.142       0.129
  90 Percent confidence interval - lower         0.111       0.101
  90 Percent confidence interval - upper         0.175       0.159
  P-value H_0: RMSEA <= 0.050                    0.000       0.000
  P-value H_0: RMSEA >= 0.080                    0.999       0.997

  Robust RMSEA                                               0.141
  90 Percent confidence interval - lower                     0.107
  90 Percent confidence interval - upper                     0.177
  P-value H_0: Robust RMSEA <= 0.050                         0.000
  P-value H_0: Robust RMSEA >= 0.080                         0.998

Standardized Root Mean Square Residual:

  SRMR                                           0.041       0.041

Parameter Estimates:
```

```
  Standard errors                                 Sandwich
  Information bread                               Observed
  Observed information based on                    Hessian

Latent Variables:
                   Estimate  Std.Err  z-value  P(>|z|)   Std.lv  Std.all
  f7 =~
    SOCI1             1.000                               1.098    0.615
    SOCI2             1.348    0.081   16.723    0.000    1.480    0.763
    SOCI3             1.000    0.092   10.918    0.000    1.098    0.635
    SOCI4             1.375    0.098   14.090    0.000    1.510    0.806
    SOCI5             1.268    0.093   13.666    0.000    1.392    0.805

Variances:
                   Estimate  Std.Err  z-value  P(>|z|)   Std.lv  Std.all
   .SOCI1             1.987    0.158   12.588    0.000    1.987    0.622
   .SOCI2             1.576    0.151   10.408    0.000    1.576    0.418
   .SOCI3             1.784    0.141   12.643    0.000    1.784    0.597
   .SOCI4             1.226    0.136    9.024    0.000    1.226    0.350
   .SOCI5             1.056    0.109    9.688    0.000    1.056    0.353
    f7                1.206    0.156    7.749    0.000    1.000    1.000
```

```
fitmeasures(sefactor, c("cfi", "tli", "rmsea","srmr", "chisq", "df"))
```

```
   cfi    tli  rmsea   srmr  chisq     df 
 0.951  0.902  0.142  0.041 61.410  5.000 
```

Technical support factor

```
m8<- 'f8=~TECH1 + TECH2 + TECH3 + TECH4 + TECH5'
tsfactor<- lavaan::cfa(m8, data=nor, estimator = "MLR")
summary(tsfactor,fit.measures=TRUE,standardized=TRUE)
```

```
lavaan 0.6.17 ended normally after 23 iterations

  Estimator                                         ML
  Optimization method                           NLMINB
  Number of model parameters                        10
```

```
  Number of observations                           560

Model Test User Model:
                                              Standard      Scaled
  Test Statistic                                61.308      44.047
  Degrees of freedom                                 5           5
  P-value (Chi-square)                           0.000       0.000
  Scaling correction factor                                  1.392
    Yuan-Bentler correction (Mplus variant)

Model Test Baseline Model:

  Test statistic                              2181.713    1237.476
  Degrees of freedom                                10          10
  P-value                                        0.000       0.000
  Scaling correction factor                                  1.763

User Model versus Baseline Model:

  Comparative Fit Index (CFI)                    0.974       0.968
  Tucker-Lewis Index (TLI)                       0.948       0.936

  Robust Comparative Fit Index (CFI)                         0.975
  Robust Tucker-Lewis Index (TLI)                            0.950

Loglikelihood and Information Criteria:

  Loglikelihood user model (H0)              -4739.691   -4739.691
  Scaling correction factor                                  1.283
      for the MLR correction
  Loglikelihood unrestricted model (H1)      -4709.038   -4709.038
  Scaling correction factor                                  1.319
      for the MLR correction

  Akaike (AIC)                                9499.383    9499.383
  Bayesian (BIC)                              9542.662    9542.662
  Sample-size adjusted Bayesian (SABIC)       9510.917    9510.917

Root Mean Square Error of Approximation:

  RMSEA                                          0.142       0.118
  90 Percent confidence interval - lower         0.111       0.092
  90 Percent confidence interval - upper         0.175       0.146
```

```
  P-value H_0: RMSEA <= 0.050                     0.000       0.000
  P-value H_0: RMSEA >= 0.080                     0.999       0.991

  Robust RMSEA                                                0.139
  90 Percent confidence interval - lower                      0.103
  90 Percent confidence interval - upper                      0.179
  P-value H_0: Robust RMSEA <= 0.050                          0.000
  P-value H_0: Robust RMSEA >= 0.080                          0.996

Standardized Root Mean Square Residual:

  SRMR                                            0.027       0.027

Parameter Estimates:

  Standard errors                              Sandwich
  Information bread                            Observed
  Observed information based on                 Hessian

Latent Variables:
                   Estimate  Std.Err  z-value  P(>|z|)   Std.lv  Std.all
  f8 =~
    TECH1             1.000                               1.283    0.737
    TECH2             1.270    0.053   24.155    0.000    1.630    0.838
    TECH3             1.434    0.067   21.535    0.000    1.841    0.925
    TECH4             1.396    0.065   21.599    0.000    1.792    0.911
    TECH5             1.184    0.065   18.278    0.000    1.520    0.771

Variances:
                   Estimate  Std.Err  z-value  P(>|z|)   Std.lv  Std.all
   .TECH1             1.385    0.103   13.431    0.000    1.385    0.457
   .TECH2             1.124    0.116    9.691    0.000    1.124    0.297
   .TECH3             0.568    0.075    7.536    0.000    0.568    0.144
   .TECH4             0.660    0.087    7.559    0.000    0.660    0.170
   .TECH5             1.573    0.155   10.118    0.000    1.573    0.405
    f8                1.647    0.152   10.817    0.000    1.000    1.000
```

```r
fitmeasures(tsfactor, c("cfi", "tli", "rmsea","srmr", "chisq", "df"))
```

```
   cfi    tli  rmsea   srmr  chisq     df 
 0.974  0.948  0.142  0.027 61.308  5.000 
```

Usefulness factor

```
m9<- 'f9=~USEF1 + USEF2 + USEF3 + USEF4 + USEF5'
usefulnessfactor<- lavaan::cfa(m9, data=nor, estimator = "MLR")
summary(usefulnessfactor,fit.measures=TRUE,standardized=TRUE)
```

```
lavaan 0.6.17 ended normally after 24 iterations

  Estimator                                         ML
  Optimization method                           NLMINB
  Number of model parameters                        10

  Number of observations                           560

Model Test User Model:
                                              Standard      Scaled
  Test Statistic                                82.538      63.601
  Degrees of freedom                                 5           5
  P-value (Chi-square)                           0.000       0.000
  Scaling correction factor                                  1.298
    Yuan-Bentler correction (Mplus variant)

Model Test Baseline Model:

  Test statistic                              1496.586    1069.871
  Degrees of freedom                                10          10
  P-value                                        0.000       0.000
  Scaling correction factor                                  1.399

User Model versus Baseline Model:

  Comparative Fit Index (CFI)                    0.948       0.945
  Tucker-Lewis Index (TLI)                       0.896       0.889

  Robust Comparative Fit Index (CFI)                         0.949
  Robust Tucker-Lewis Index (TLI)                            0.897

Loglikelihood and Information Criteria:

  Loglikelihood user model (H0)              -5005.774   -5005.774
  Scaling correction factor                                  0.985
      for the MLR correction
```

```
  Loglikelihood unrestricted model (H1)      -4964.505   -4964.505
  Scaling correction factor                                  1.090
      for the MLR correction

  Akaike (AIC)                               10031.548   10031.548
  Bayesian (BIC)                             10074.827   10074.827
  Sample-size adjusted Bayesian (SABIC)      10043.083   10043.083

Root Mean Square Error of Approximation:

  RMSEA                                          0.166       0.145
  90 Percent confidence interval - lower         0.136       0.118
  90 Percent confidence interval - upper         0.199       0.173
  P-value H_0: RMSEA <= 0.050                    0.000       0.000
  P-value H_0: RMSEA >= 0.080                    1.000       1.000

  Robust RMSEA                                               0.165
  90 Percent confidence interval - lower                     0.130
  90 Percent confidence interval - upper                     0.202
  P-value H_0: Robust RMSEA <= 0.050                         0.000
  P-value H_0: Robust RMSEA >= 0.080                         1.000

Standardized Root Mean Square Residual:

  SRMR                                           0.043       0.043

Parameter Estimates:

  Standard errors                             Sandwich
  Information bread                           Observed
  Observed information based on                Hessian

Latent Variables:
                   Estimate  Std.Err  z-value  P(>|z|)   Std.lv  Std.all
  f9 =~
    USEF1             1.000                               1.634    0.872
    USEF2             0.994    0.028   35.768    0.000    1.625    0.884
    USEF3             0.804    0.046   17.316    0.000    1.314    0.726
    USEF4             0.782    0.045   17.233    0.000    1.278    0.656
    USEF5             0.763    0.046   16.465    0.000    1.247    0.679

Variances:
                   Estimate  Std.Err  z-value  P(>|z|)   Std.lv  Std.all
```

```
   .USEF1             0.845    0.094    8.951    0.000    0.845    0.240
   .USEF2             0.742    0.086    8.614    0.000    0.742    0.219
   .USEF3             1.554    0.127   12.234    0.000    1.554    0.474
   .USEF4             2.160    0.140   15.413    0.000    2.160    0.569
   .USEF5             1.820    0.135   13.467    0.000    1.820    0.539
    f9                2.671    0.168   15.901    0.000    1.000    1.000
```

```
fitmeasures(usefulnessfactor, c("cfi", "tli", "rmsea","srmr", "chisq", "df"))
```

```
   cfi    tli  rmsea   srmr  chisq     df 
 0.948  0.896  0.166  0.043 82.538  5.000 
```

Unreliability factor

```
m10<- 'f10=~UNRE1 + UNRE2 + UNRE3 + UNRE4 + UNRE5'
unreliabilityfactor<- lavaan::cfa(m10,data=nor, estimator = "MLR")
summary(unreliabilityfactor,fit.measures=TRUE,standardized=TRUE)
```

```
lavaan 0.6.17 ended normally after 21 iterations

  Estimator                                         ML
  Optimization method                           NLMINB
  Number of model parameters                        10

  Number of observations                           560

Model Test User Model:
                                              Standard      Scaled
  Test Statistic                                74.152      47.278
  Degrees of freedom                                 5           5
  P-value (Chi-square)                           0.000       0.000
  Scaling correction factor                                  1.568
    Yuan-Bentler correction (Mplus variant)                       

Model Test Baseline Model:

  Test statistic                              2175.519    1186.715
  Degrees of freedom                                10          10
  P-value                                        0.000       0.000
```

```
  Scaling correction factor                                  1.833

User Model versus Baseline Model:

  Comparative Fit Index (CFI)                    0.968       0.964
  Tucker-Lewis Index (TLI)                       0.936       0.928

  Robust Comparative Fit Index (CFI)                         0.969
  Robust Tucker-Lewis Index (TLI)                            0.939

Loglikelihood and Information Criteria:

  Loglikelihood user model (H0)              -4702.935   -4702.935
  Scaling correction factor                                  1.267
      for the MLR correction
  Loglikelihood unrestricted model (H1)      -4665.859   -4665.859
  Scaling correction factor                                  1.368
      for the MLR correction

  Akaike (AIC)                                9425.871    9425.871
  Bayesian (BIC)                              9469.150    9469.150
  Sample-size adjusted Bayesian (SABIC)       9437.405    9437.405

Root Mean Square Error of Approximation:

  RMSEA                                          0.157       0.123
  90 Percent confidence interval - lower         0.127       0.098
  90 Percent confidence interval - upper         0.190       0.149
  P-value H_0: RMSEA <= 0.050                    0.000       0.000
  P-value H_0: RMSEA >= 0.080                    1.000       0.998

  Robust RMSEA                                               0.154
  90 Percent confidence interval - lower                     0.116
  90 Percent confidence interval - upper                     0.195
  P-value H_0: Robust RMSEA <= 0.050                         0.000
  P-value H_0: Robust RMSEA >= 0.080                         0.999

Standardized Root Mean Square Residual:

  SRMR                                           0.025       0.025

Parameter Estimates:
```

```
  Standard errors                             Sandwich
  Information bread                           Observed
  Observed information based on                Hessian

Latent Variables:
                   Estimate  Std.Err  z-value  P(>|z|)   Std.lv  Std.all
  f10 =~
    UNRE1             1.000                               1.525    0.818
    UNRE2             1.098    0.034   32.360    0.000    1.675    0.883
    UNRE3             1.015    0.045   22.595    0.000    1.548    0.852
    UNRE4             1.092    0.044   24.850    0.000    1.665    0.901
    UNRE5             1.013    0.042   24.413    0.000    1.545    0.764

Variances:
                   Estimate  Std.Err  z-value  P(>|z|)   Std.lv  Std.all
   .UNRE1             1.146    0.117    9.775    0.000    1.146    0.330
   .UNRE2             0.797    0.103    7.764    0.000    0.797    0.221
   .UNRE3             0.909    0.112    8.102    0.000    0.909    0.275
   .UNRE4             0.644    0.072    8.904    0.000    0.644    0.188
   .UNRE5             1.703    0.134   12.705    0.000    1.703    0.416
    f10               2.325    0.175   13.260    0.000    1.000    1.000
```

```
fitmeasures(unreliabilityfactor, c("cfi", "tli", "rmsea","srmr", "chisq", "df"))
```

```
   cfi    tli  rmsea   srmr  chisq     df 
 0.968  0.936  0.157  0.025 74.152  5.000 
```

CFA for the whole model

```
#put all 1 factor cfas in list
cfasyntaxlist <- list(COMP="COMP=~COMP1 + COMP2 + COMP3 + COMP4 + COMP5",
                      CONF="CONF=~CONF1 + CONF2 + CONF3 + CONF4 + CONF5",
                      INSE="INSE =~ INSE1 + INSE2 + INSE3 + INSE4 + INSE5",
                      PRIV="PRIV =~ PRIV1 + PRIV2 + PRIV3 + PRIV4 + PRIV5",
                      OVER="OVER =~ OVER1 + OVER2 + OVER3 + OVER4+ OVER5",
                      SAFE="SAFE =~ SAFE1 + SAFE2 + SAFE3 + SAFE4 + SAFE5",
                      SOCI="SOCI=~ SOCI1 + SOCI2 + SOCI3 + SOCI4 + SOCI5",
                      TECH= "TECH =~ TECH1 + TECH2 + TECH3 + TECH4 + TECH5",
                      USEF ="USEF =~ USEF1 + USEF2 + USEF3 + USEF4 + USEF5",
                      UNRE ="UNRE =~ UNRE1 + UNRE2 + UNRE3 + UNRE4 + UNRE5"
```

```
)



cfas <- lapply(cfasyntaxlist, function(model){
  f <- lavaan::cfa(model, nor, estimator="MLR", std.lv=T)
})

table1 <- lapply(cfas, function(f){
  fms <-  lavaan::fitmeasures(f, c("chisq.scaled", "rmsea.robust", "cfi.robust", "srmr"))
  loadings <- lavaan::standardizedsolution(f)$est.std[1:5]
  return(c(fms, loadings))
})

tab1.df <- do.call(rbind, table1)
colnames(tab1.df) <- c("chisq", "rmsea", "cfi", "srmr", paste0("l", 1:5))
print(xtable::xtable(tab1.df))
```

```
% latex table generated in R 4.1.3 by xtable 1.8-4 package
% Tue Jan 16 10:27:48 2024
\begin{table}[ht]
\centering
\begin{tabular}{rrrrrrrrrr}
  \hline
 & chisq & rmsea & cfi & srmr & l1 & l2 & l3 & l4 & l5 \\
  \hline
COMP & 194.92 & 0.30 & 0.89 & 0.05 & 0.83 & 0.84 & 0.85 & 0.83 & 0.85 \\
  CONF & 29.21 & 0.13 & 0.98 & 0.01 & 0.80 & 0.92 & 0.91 & 0.93 & 0.92 \\
  INSE & 32.44 & 0.17 & 0.96 & 0.04 & 0.64 & 0.95 & 0.88 & 0.86 & 0.89 \\
  PRIV & 248.44 & 0.35 & 0.82 & 0.08 & 0.73 & 0.79 & 0.83 & 0.84 & 0.79 \\
  OVER & 125.25 & 0.23 & 0.91 & 0.07 & 0.90 & 0.91 & 0.62 & 0.70 & 0.66 \\
  SAFE & 163.25 & 0.31 & 0.85 & 0.07 & 0.84 & 0.84 & 0.71 & 0.83 & 0.74 \\
  SOCI & 51.28 & 0.14 & 0.95 & 0.04 & 0.61 & 0.76 & 0.63 & 0.81 & 0.80 \\
  TECH & 44.05 & 0.14 & 0.97 & 0.03 & 0.74 & 0.84 & 0.93 & 0.91 & 0.77 \\
  USEF & 63.60 & 0.16 & 0.95 & 0.04 & 0.87 & 0.88 & 0.73 & 0.66 & 0.68 \\
  UNRE & 47.28 & 0.15 & 0.97 & 0.03 & 0.82 & 0.88 & 0.85 & 0.90 & 0.76 \\
   \hline
\end{tabular}
\end{table}
```

```
firstorder <- paste(cfasyntaxlist, collapse=";")
f1 <- lavaan::cfa(firstorder, nor, estimator="MLR", std.lv=T)
fitmeasures(f1, c("cfi", "tli", "rmsea", "srmr", "chisq", "df"))
```

```
     cfi      tli    rmsea     srmr    chisq       df
   0.888    0.879    0.065    0.063 3822.883 1130.000
```

Second order reflective model

```
secondorder <- paste("DS =~",paste(names(cfasyntaxlist), collapse="+"))
full <- paste(secondorder, "\n", firstorder)
f2 <- lavaan::cfa(full, nor, estimator="MLR", std.lv=T)
lavaan::standardizedsolution(f2)
```

```
    lhs op   rhs est.std    se       z pvalue ci.lower ci.upper
1    DS =~  COMP   0.742 0.027  27.942      0    0.690    0.794
2    DS =~  CONF   0.541 0.040  13.588      0    0.463    0.619
3    DS =~  INSE   0.432 0.041  10.407      0    0.351    0.513
4    DS =~  PRIV   0.595 0.040  14.889      0    0.516    0.673
5    DS =~  OVER   0.823 0.027  31.037      0    0.771    0.875
6    DS =~  SAFE   0.582 0.040  14.434      0    0.503    0.661
7    DS =~  SOCI   0.796 0.029  27.476      0    0.739    0.853
8    DS =~  TECH   0.796 0.023  34.022      0    0.750    0.842
9    DS =~  USEF   0.882 0.022  41.012      0    0.840    0.924
10   DS =~  UNRE   0.767 0.027  28.446      0    0.714    0.819
11 COMP =~ COMP1   0.825 0.023  36.252      0    0.781    0.870
12 COMP =~ COMP2   0.839 0.019  43.089      0    0.801    0.878
13 COMP =~ COMP3   0.854 0.016  52.584      0    0.823    0.886
14 COMP =~ COMP4   0.833 0.023  36.639      0    0.788    0.877
15 COMP =~ COMP5   0.850 0.019  44.411      0    0.813    0.888
16 CONF =~ CONF1   0.801 0.020  40.748      0    0.762    0.840
17 CONF =~ CONF2   0.916 0.011  85.905      0    0.895    0.937
18 CONF =~ CONF3   0.912 0.014  66.967      0    0.885    0.939
19 CONF =~ CONF4   0.933 0.009 104.702      0    0.915    0.950
20 CONF =~ CONF5   0.922 0.013  72.161      0    0.897    0.947
21 INSE =~ INSE1   0.643 0.039  16.445      0    0.567    0.720
22 INSE =~ INSE2   0.951 0.009 100.641      0    0.932    0.969
23 INSE =~ INSE3   0.877 0.024  37.150      0    0.831    0.924
24 INSE =~ INSE4   0.859 0.025  34.872      0    0.811    0.908
25 INSE =~ INSE5   0.884 0.022  40.074      0    0.841    0.927
```

26	PRIV	=~	PRIV1	0.763	0.044	17.468	0	0.678	0.849
27	PRIV	=~	PRIV2	0.816	0.037	22.232	0	0.744	0.888
28	PRIV	=~	PRIV3	0.808	0.031	26.150	0	0.748	0.869
29	PRIV	=~	PRIV4	0.832	0.030	27.591	0	0.773	0.891
30	PRIV	=~	PRIV5	0.773	0.038	20.184	0	0.698	0.848
31	OVER	=~	OVER1	0.877	0.021	41.194	0	0.835	0.919
32	OVER	=~	OVER2	0.900	0.020	44.173	0	0.860	0.939
33	OVER	=~	OVER3	0.642	0.034	18.608	0	0.574	0.709
34	OVER	=~	OVER4	0.714	0.031	22.753	0	0.653	0.776
35	OVER	=~	OVER5	0.694	0.032	21.560	0	0.631	0.757
36	SAFE	=~	SAFE1	0.842	0.040	21.159	0	0.764	0.920
37	SAFE	=~	SAFE2	0.836	0.037	22.677	0	0.763	0.908
38	SAFE	=~	SAFE3	0.711	0.032	22.097	0	0.648	0.774
39	SAFE	=~	SAFE4	0.822	0.039	21.148	0	0.746	0.899
40	SAFE	=~	SAFE5	0.742	0.047	15.624	0	0.649	0.835
41	SOCI	=~	SOCI1	0.646	0.033	19.411	0	0.580	0.711
42	SOCI	=~	SOCI2	0.769	0.024	32.574	0	0.723	0.815
43	SOCI	=~	SOCI3	0.617	0.035	17.424	0	0.548	0.687
44	SOCI	=~	SOCI4	0.796	0.024	33.387	0	0.749	0.843
45	SOCI	=~	SOCI5	0.800	0.021	37.521	0	0.758	0.841
46	TECH	=~	TECH1	0.762	0.022	34.591	0	0.719	0.805
47	TECH	=~	TECH2	0.848	0.018	48.040	0	0.814	0.883
48	TECH	=~	TECH3	0.912	0.012	76.731	0	0.889	0.936
49	TECH	=~	TECH4	0.903	0.014	65.726	0	0.876	0.930
50	TECH	=~	TECH5	0.778	0.025	31.514	0	0.729	0.826
51	USEF	=~	USEF1	0.857	0.016	51.971	0	0.825	0.890
52	USEF	=~	USEF2	0.862	0.016	54.743	0	0.832	0.893
53	USEF	=~	USEF3	0.740	0.026	28.727	0	0.689	0.790
54	USEF	=~	USEF4	0.679	0.027	25.192	0	0.626	0.732
55	USEF	=~	USEF5	0.706	0.027	26.110	0	0.653	0.760
56	UNRE	=~	UNRE1	0.825	0.020	41.596	0	0.786	0.864
57	UNRE	=~	UNRE2	0.877	0.016	55.255	0	0.846	0.908
58	UNRE	=~	UNRE3	0.848	0.020	42.652	0	0.809	0.887
59	UNRE	=~	UNRE4	0.900	0.012	77.753	0	0.877	0.923
60	UNRE	=~	UNRE5	0.771	0.021	37.211	0	0.730	0.811
61	COMP1	~~	COMP1	0.319	0.038	8.492	0	0.245	0.393
62	COMP2	~~	COMP2	0.295	0.033	9.030	0	0.231	0.359
63	COMP3	~~	COMP3	0.270	0.028	9.723	0	0.216	0.324
64	COMP4	~~	COMP4	0.306	0.038	8.087	0	0.232	0.380
65	COMP5	~~	COMP5	0.277	0.033	8.497	0	0.213	0.341
66	CONF1	~~	CONF1	0.358	0.031	11.379	0	0.297	0.420
67	CONF2	~~	CONF2	0.160	0.020	8.208	0	0.122	0.199
68	CONF3	~~	CONF3	0.168	0.025	6.775	0	0.120	0.217

69	CONF4	~~	CONF4	0.130	0.017	7.828	0	0.098	0.163
70	CONF5	~~	CONF5	0.151	0.024	6.403	0	0.105	0.197
71	INSE1	~~	INSE1	0.586	0.050	11.634	0	0.487	0.685
72	INSE2	~~	INSE2	0.096	0.018	5.345	0	0.061	0.131
73	INSE3	~~	INSE3	0.230	0.041	5.556	0	0.149	0.311
74	INSE4	~~	INSE4	0.261	0.042	6.168	0	0.178	0.344
75	INSE5	~~	INSE5	0.218	0.039	5.599	0	0.142	0.295
76	PRIV1	~~	PRIV1	0.417	0.067	6.256	0	0.287	0.548
77	PRIV2	~~	PRIV2	0.334	0.060	5.573	0	0.216	0.451
78	PRIV3	~~	PRIV3	0.347	0.050	6.940	0	0.249	0.445
79	PRIV4	~~	PRIV4	0.308	0.050	6.133	0	0.209	0.406
80	PRIV5	~~	PRIV5	0.403	0.059	6.816	0	0.287	0.519
81	OVER1	~~	OVER1	0.231	0.037	6.188	0	0.158	0.304
82	OVER2	~~	OVER2	0.191	0.037	5.210	0	0.119	0.263
83	OVER3	~~	OVER3	0.588	0.044	13.302	0	0.502	0.675
84	OVER4	~~	OVER4	0.490	0.045	10.937	0	0.402	0.578
85	OVER5	~~	OVER5	0.518	0.045	11.585	0	0.430	0.606
86	SAFE1	~~	SAFE1	0.291	0.067	4.339	0	0.159	0.422
87	SAFE2	~~	SAFE2	0.302	0.062	4.900	0	0.181	0.422
88	SAFE3	~~	SAFE3	0.495	0.046	10.818	0	0.405	0.584
89	SAFE4	~~	SAFE4	0.324	0.064	5.061	0	0.198	0.449
90	SAFE5	~~	SAFE5	0.450	0.070	6.392	0	0.312	0.588
91	SOCI1	~~	SOCI1	0.583	0.043	13.587	0	0.499	0.667
92	SOCI2	~~	SOCI2	0.408	0.036	11.244	0	0.337	0.480
93	SOCI3	~~	SOCI3	0.619	0.044	14.147	0	0.533	0.705
94	SOCI4	~~	SOCI4	0.366	0.038	9.639	0	0.292	0.440
95	SOCI5	~~	SOCI5	0.360	0.034	10.574	0	0.294	0.427
96	TECH1	~~	TECH1	0.420	0.034	12.508	0	0.354	0.485
97	TECH2	~~	TECH2	0.280	0.030	9.364	0	0.222	0.339
98	TECH3	~~	TECH3	0.168	0.022	7.736	0	0.125	0.210
99	TECH4	~~	TECH4	0.185	0.025	7.468	0	0.137	0.234
100	TECH5	~~	TECH5	0.395	0.038	10.308	0	0.320	0.471
101	USEF1	~~	USEF1	0.265	0.028	9.366	0	0.209	0.320
102	USEF2	~~	USEF2	0.256	0.027	9.431	0	0.203	0.310
103	USEF3	~~	USEF3	0.452	0.038	11.868	0	0.378	0.527
104	USEF4	~~	USEF4	0.539	0.037	14.745	0	0.468	0.611
105	USEF5	~~	USEF5	0.501	0.038	13.102	0	0.426	0.576
106	UNRE1	~~	UNRE1	0.319	0.033	9.748	0	0.255	0.383
107	UNRE2	~~	UNRE2	0.230	0.028	8.266	0	0.176	0.285
108	UNRE3	~~	UNRE3	0.281	0.034	8.344	0	0.215	0.347
109	UNRE4	~~	UNRE4	0.190	0.021	9.104	0	0.149	0.231
110	UNRE5	~~	UNRE5	0.406	0.032	12.703	0	0.343	0.468
111	DS	~~	DS	1.000	0.000	NA	NA	1.000	1.000

```
112  COMP ~~  COMP   0.450 0.039  11.435      0    0.373    0.527
113  CONF ~~  CONF   0.707 0.043  16.404      0    0.623    0.792
114  INSE ~~  INSE   0.814 0.036  22.700      0    0.743    0.884
115  PRIV ~~  PRIV   0.646 0.048  13.605      0    0.553    0.739
116  OVER ~~  OVER   0.322 0.044   7.374      0    0.237    0.408
117  SAFE ~~  SAFE   0.661 0.047  14.073      0    0.569    0.753
118  SOCI ~~  SOCI   0.366 0.046   7.928      0    0.275    0.456
119  TECH ~~  TECH   0.366 0.037   9.837      0    0.293    0.439
120  USEF ~~  USEF   0.222 0.038   5.863      0    0.148    0.297
121  UNRE ~~  UNRE   0.412 0.041   9.981      0    0.331    0.493
```

```
lavaan::fitmeasures(f2, c("chisq.scaled", "rmsea.robust", "cfi.robust", "srmr"))
```

```
chisq.scaled rmsea.robust   cfi.robust         srmr
    3778.571        0.068        0.873        0.087
```

```
anova(f1,f2)
```

```
Scaled Chi-Squared Difference Test (method = "satorra.bentler.2001")

lavaan NOTE:
    The "Chisq" column contains standard test statistics, not the
    robust test that should be reported per model. A robust difference
    test is a function of two standard (not robust) statistics.

     Df   AIC   BIC  Chisq Chisq diff Df diff Pr(>Chisq)
f1 1130 90292 90920 3822.9
f2 1165 90793 91269 4393.7     547.03      35  < 2.2e-16 ***
---
Signif. codes:  0 '***' 0.001 '**' 0.01 '*' 0.05 '.' 0.1 ' ' 1
```

Modification indices

MI for bad fitting models

```
modindices(cfas$PRIV) %>% arrange(-mi) %>% head(5)
```

```
    lhs op   rhs      mi    epc sepc.lv sepc.all sepc.nox
1 PRIV1 ~~ PRIV2 321.059  1.238   1.238    0.964    0.964
2 PRIV2 ~~ PRIV5  59.410 -0.498  -0.498   -0.442   -0.442
3 PRIV3 ~~ PRIV5  58.169  0.463   0.463    0.469    0.469
4 PRIV1 ~~ PRIV5  50.813 -0.468  -0.468   -0.379   -0.379
5 PRIV4 ~~ PRIV5  31.794  0.336   0.336    0.360    0.360
```

```
modindices(cfas$COMP) %>% arrange(-mi)  %>% head(5)
```

```
    lhs op   rhs      mi    epc sepc.lv sepc.all sepc.nox
1 COMP4 ~~ COMP5 242.517  1.112   1.112    0.925    0.925
2 COMP1 ~~ COMP2 100.365  0.611   0.611    0.587    0.587
3 COMP1 ~~ COMP5  64.345 -0.511  -0.511   -0.479   -0.479
4 COMP2 ~~ COMP4  55.332 -0.509  -0.509   -0.433   -0.433
5 COMP1 ~~ COMP4  40.085 -0.397  -0.397   -0.358   -0.358
```

```
modindices(cfas$SAFE) %>% arrange(-mi) %>% head(5)
```

```
    lhs op   rhs      mi    epc sepc.lv sepc.all sepc.nox
1 SAFE4 ~~ SAFE5 235.528  0.958   0.958    0.882    0.882
2 SAFE1 ~~ SAFE2 190.648  0.738   0.738    1.015    1.015
3 SAFE1 ~~ SAFE4  86.563 -0.548  -0.548   -0.661   -0.661
4 SAFE2 ~~ SAFE5  59.726 -0.433  -0.433   -0.455   -0.455
5 SAFE1 ~~ SAFE5  47.079 -0.377  -0.377   -0.406   -0.406
```

```
modindices(cfas$OVER) %>% arrange(-mi)  %>% head(5)
```

```
    lhs op   rhs      mi    epc sepc.lv sepc.all sepc.nox
1 OVER1 ~~ OVER2 177.712  1.507   1.507    2.236    2.236
2 OVER4 ~~ OVER5  61.815  0.631   0.631    0.365    0.365
3 OVER3 ~~ OVER4  60.894  0.656   0.656    0.359    0.359
4 OVER2 ~~ OVER4  29.669 -0.398  -0.398   -0.401   -0.401
5 OVER3 ~~ OVER5  26.073  0.477   0.477    0.233    0.233
```

MI for good fitting models for comparison

```
modindices(cfas$TECH) %>% arrange(-mi) %>% head(5)
```

```
    lhs op   rhs     mi    epc sepc.lv sepc.all sepc.nox
1 TECH1 ~~ TECH2 46.969  0.421   0.421    0.337    0.337
2 TECH2 ~~ TECH4 23.322 -0.287  -0.287   -0.334   -0.334
3 TECH3 ~~ TECH4 14.240  0.255   0.255    0.417    0.417
4 TECH4 ~~ TECH5 11.178  0.205   0.205    0.201    0.201
5 TECH1 ~~ TECH3  9.126 -0.167  -0.167   -0.188   -0.188
```

```
modindices(cfas$UNRE) %>% arrange(-mi)  %>% head(5)
```

```
    lhs op   rhs     mi    epc sepc.lv sepc.all sepc.nox
1 UNRE3 ~~ UNRE4 42.989  0.354   0.354    0.463    0.463
2 UNRE1 ~~ UNRE2 39.315  0.360   0.360    0.377    0.377
3 UNRE2 ~~ UNRE4 36.594 -0.346  -0.346   -0.484   -0.484
4 UNRE1 ~~ UNRE3 18.956 -0.246  -0.246   -0.241   -0.241
5 UNRE3 ~~ UNRE5 14.727 -0.250  -0.250   -0.201   -0.201
```

```
modindices(cfas$CONF) %>% arrange(-mi) %>% head(5)
```

```
    lhs op   rhs     mi    epc sepc.lv sepc.all sepc.nox
1 CONF2 ~~ CONF3 48.742  0.248   0.248    0.441    0.441
2 CONF4 ~~ CONF5 40.075  0.203   0.203    0.450    0.450
3 CONF2 ~~ CONF5 28.412 -0.177  -0.177   -0.347   -0.347
4 CONF3 ~~ CONF4 13.935 -0.128  -0.128   -0.257   -0.257
5 CONF2 ~~ CONF4  7.166 -0.086  -0.086   -0.188   -0.188
```

MI for the rest

```
modindices(cfas$INSE) %>% arrange(-mi) %>% head(5)
```

```
    lhs op   rhs     mi    epc sepc.lv sepc.all sepc.nox
1 INSE4 ~~ INSE5 59.378  0.184   0.184    0.444    0.444
2 INSE1 ~~ INSE4 40.537 -0.225  -0.225   -0.301   -0.301
3 INSE2 ~~ INSE5 25.298 -0.128  -0.128   -0.497   -0.497
```

```
4 INSE1 ~~ INSE3 23.149  0.174   0.174    0.231    0.231
5 INSE3 ~~ INSE4 22.198 -0.124  -0.124   -0.264   -0.264
```

```
modindices(cfas$SOCI) %>% arrange(-mi)  %>% head(5)
```

```
    lhs op   rhs     mi    epc sepc.lv sepc.all sepc.nox
1 SOCI1 ~~ SOCI2 34.385  0.555   0.555    0.314    0.314
2 SOCI3 ~~ SOCI5 26.372  0.414   0.414    0.302    0.302
3 SOCI2 ~~ SOCI3 18.914 -0.396  -0.396   -0.236   -0.236
4 SOCI2 ~~ SOCI5 14.596 -0.363  -0.363   -0.281   -0.281
5 SOCI1 ~~ SOCI4 11.861 -0.311  -0.311   -0.200   -0.200
```

```
modindices(cfas$USEF) %>% arrange(-mi) %>% head(5)
```

```
    lhs op   rhs     mi    epc sepc.lv sepc.all sepc.nox
1 USEF1 ~~ USEF2 59.937  0.737   0.737    0.931    0.931
2 USEF1 ~~ USEF3 38.175 -0.476  -0.476   -0.415   -0.415
3 USEF3 ~~ USEF4 34.083  0.515   0.515    0.281    0.281
4 USEF3 ~~ USEF5 26.290  0.419   0.419    0.249    0.249
5 USEF2 ~~ USEF4 19.770 -0.359  -0.359   -0.284   -0.284
```

CFA with MI

```
cfasyntaxlistMI <- c(
  "COMP =~ COMP1 + COMP2 + COMP3 + COMP4 + COMP5",
  "CONF =~ CONF1 + CONF2 + CONF3 + CONF4 + CONF5",
  "INSE =~ INSE1 + INSE2 + INSE3 + INSE4 + INSE5",
  "PRIV =~ PRIV1 + PRIV2 + PRIV3 + PRIV4 + PRIV5",
  "OVER =~ OVER1 + OVER2 + OVER3 + OVER4 + OVER5",
  "SAFE =~ SAFE1 + SAFE2 + SAFE3 + SAFE4 + SAFE5",
  "SOCI =~ SOCI1 + SOCI2 + SOCI3 + SOCI4 + SOCI5",
  "TECH =~ TECH1 + TECH2 + TECH3 + TECH4 + TECH5",
  "USEF =~ USEF1 + USEF2 + USEF3 + USEF4 + USEF5",
  "UNRE =~ UNRE1 + UNRE2 + UNRE3 + UNRE4 + UNRE5",
  "COMP1 ~~ COMP2",
  "COMP4 ~~ COMP5",
  "CONF2 ~~ CONF3",
  "PRIV1 ~~ PRIV2",
  "OVER1 ~~ OVER2",
```

```r
  "SAFE4 ~~ SAFE5",
  "USEF1 ~~ USEF2",
  "INSE4 ~~ INSE5",
  "SAFE1 ~~ SAFE2",
  "TECH1 ~~ TECH2"
)

cfasMI <- lapply(cfasyntaxlistMI, function(model) {
  fMI <- lavaan::cfa(model, data = nor, estimator = "MLR", std.lv = TRUE)
})

wholemodelMI <- lavaan::cfa(cfasyntaxlistMI, data = nor, estimator = "MLR")

summary(wholemodelMI, fit.measures = TRUE, standardized = TRUE)
```

```
lavaan 0.6.17 ended normally after 100 iterations

  Estimator                                         ML
  Optimization method                           NLMINB
  Number of model parameters                       155

  Number of observations                           560

Model Test User Model:
                                              Standard      Scaled
  Test Statistic                              2637.978    2277.387
  Degrees of freedom                              1120        1120
  P-value (Chi-square)                           0.000       0.000
  Scaling correction factor                                  1.158
    Yuan-Bentler correction (Mplus variant)

Model Test Baseline Model:

  Test statistic                             25360.741   20929.029
  Degrees of freedom                              1225        1225
  P-value                                        0.000       0.000
  Scaling correction factor                                  1.212

User Model versus Baseline Model:

  Comparative Fit Index (CFI)                    0.937       0.941
  Tucker-Lewis Index (TLI)                       0.931       0.936
```

```
  Robust Comparative Fit Index (CFI)                         0.944
  Robust Tucker-Lewis Index (TLI)                            0.939

Loglikelihood and Information Criteria:

  Loglikelihood user model (H0)          -44408.568  -44408.568
  Scaling correction factor                               1.440
      for the MLR correction
  Loglikelihood unrestricted model (H1)  -43089.579  -43089.579
  Scaling correction factor                               1.193
      for the MLR correction

  Akaike (AIC)                            89127.136   89127.136
  Bayesian (BIC)                          89797.966   89797.966
  Sample-size adjusted Bayesian (SABIC)   89305.920   89305.920

Root Mean Square Error of Approximation:

  RMSEA                                       0.049       0.043
  90 Percent confidence interval - lower      0.047       0.041
  90 Percent confidence interval - upper      0.052       0.045
  P-value H_0: RMSEA <= 0.050                 0.704       1.000
  P-value H_0: RMSEA >= 0.080                 0.000       0.000

  Robust RMSEA                                            0.046
  90 Percent confidence interval - lower                  0.044
  90 Percent confidence interval - upper                  0.049
  P-value H_0: Robust RMSEA <= 0.050                      0.989
  P-value H_0: Robust RMSEA >= 0.080                      0.000

Standardized Root Mean Square Residual:

  SRMR                                        0.065       0.065

Parameter Estimates:

  Standard errors                          Sandwich
  Information bread                        Observed
  Observed information based on             Hessian

Latent Variables:
                   Estimate  Std.Err  z-value  P(>|z|)   Std.lv  Std.all
```

COMP =~						
COMP1	1.000				1.450	0.819
COMP2	1.118	0.034	32.835	0.000	1.621	0.828
COMP3	1.099	0.038	28.977	0.000	1.592	0.885
COMP4	1.062	0.050	21.198	0.000	1.539	0.776
COMP5	1.136	0.048	23.487	0.000	1.646	0.801
CONF =~						
CONF1	1.000				1.502	0.801
CONF2	1.079	0.035	30.922	0.000	1.621	0.894
CONF3	1.144	0.040	28.452	0.000	1.719	0.892
CONF4	1.103	0.038	29.037	0.000	1.657	0.939
CONF5	1.124	0.038	29.537	0.000	1.688	0.933
INSE =~						
INSE1	1.000				0.923	0.650
INSE2	1.437	0.104	13.825	0.000	1.327	0.961
INSE3	1.354	0.089	15.272	0.000	1.251	0.880
INSE4	1.223	0.116	10.573	0.000	1.129	0.838
INSE5	1.218	0.100	12.195	0.000	1.125	0.863
PRIV =~						
PRIV1	1.000				1.130	0.647
PRIV2	1.135	0.040	28.454	0.000	1.283	0.718
PRIV3	1.286	0.071	18.036	0.000	1.453	0.858
PRIV4	1.247	0.067	18.641	0.000	1.410	0.847
PRIV5	1.234	0.074	16.751	0.000	1.395	0.827
OVER =~						
OVER1	1.000				1.406	0.743
OVER2	1.072	0.034	31.087	0.000	1.507	0.775
OVER3	0.946	0.058	16.332	0.000	1.330	0.706
OVER4	0.954	0.050	19.234	0.000	1.341	0.776
OVER5	1.028	0.052	19.708	0.000	1.445	0.779
SAFE =~						
SAFE1	1.000				1.248	0.807
SAFE2	0.988	0.036	27.572	0.000	1.232	0.782
SAFE3	1.012	0.063	16.069	0.000	1.262	0.749
SAFE4	1.111	0.067	16.523	0.000	1.386	0.793
SAFE5	0.915	0.067	13.692	0.000	1.141	0.695
SOCI =~						
SOCI1	1.000				1.167	0.653
SOCI2	1.316	0.072	18.272	0.000	1.537	0.792
SOCI3	0.883	0.076	11.570	0.000	1.031	0.596
SOCI4	1.271	0.081	15.731	0.000	1.483	0.792
SOCI5	1.165	0.074	15.651	0.000	1.360	0.786
TECH =~						

```
    TECH1             1.000                               1.284    0.738
    TECH2             1.258    0.053   23.633    0.000    1.615    0.831
    TECH3             1.419    0.066   21.448    0.000    1.822    0.916
    TECH4             1.402    0.064   21.762    0.000    1.801    0.915
    TECH5             1.198    0.064   18.585    0.000    1.539    0.781
  USEF =~
    USEF1             1.000                               1.518    0.810
    USEF2             0.982    0.029   34.070    0.000    1.491    0.811
    USEF3             0.903    0.045   19.900    0.000    1.370    0.756
    USEF4             0.877    0.047   18.754    0.000    1.332    0.684
    USEF5             0.886    0.045   19.589    0.000    1.346    0.733
  UNRE =~
    UNRE1             1.000                               1.539    0.826
    UNRE2             1.080    0.032   33.385    0.000    1.662    0.876
    UNRE3             1.001    0.042   23.791    0.000    1.541    0.847
    UNRE4             1.082    0.041   26.621    0.000    1.666    0.901
    UNRE5             1.013    0.040   25.560    0.000    1.559    0.771

Covariances:
                   Estimate  Std.Err  z-value  P(>|z|)   Std.lv  Std.all
 .COMP1 ~~
   .COMP2             0.391    0.088    4.456    0.000    0.391    0.351
 .COMP4 ~~
   .COMP5             0.896    0.111    8.064    0.000    0.896    0.583
 .CONF2 ~~
   .CONF3             0.256    0.056    4.538    0.000    0.256    0.362
 .PRIV1 ~~
   .PRIV2             1.138    0.110   10.328    0.000    1.138    0.687
 .OVER1 ~~
   .OVER2             0.978    0.123    7.934    0.000    0.978    0.629
 .SAFE4 ~~
   .SAFE5             0.693    0.119    5.843    0.000    0.693    0.551
 .USEF1 ~~
   .USEF2             0.483    0.085    5.667    0.000    0.483    0.408
 .INSE4 ~~
   .INSE5             0.169    0.056    3.027    0.002    0.169    0.350
 .SAFE1 ~~
   .SAFE2             0.417    0.094    4.431    0.000    0.417    0.464
 .TECH1 ~~
   .TECH2             0.328    0.087    3.786    0.000    0.328    0.258
  COMP ~~
    CONF              0.781    0.114    6.877    0.000    0.359    0.359
    INSE              0.418    0.079    5.299    0.000    0.313    0.313
```

```
    PRIV              0.565    0.095    5.933    0.000    0.345    0.345
    OVER              1.268    0.126   10.025    0.000    0.622    0.622
    SAFE              0.739    0.098    7.506    0.000    0.409    0.409
    SOCI              0.779    0.095    8.239    0.000    0.460    0.460
    TECH              1.100    0.105   10.493    0.000    0.591    0.591
    USEF              1.766    0.121   14.604    0.000    0.803    0.803
    UNRE              1.189    0.117   10.142    0.000    0.533    0.533
  CONF ~~
    INSE              0.354    0.091    3.910    0.000    0.255    0.255
    PRIV              0.656    0.100    6.587    0.000    0.386    0.386
    OVER              1.210    0.125    9.692    0.000    0.573    0.573
    SAFE              0.701    0.110    6.391    0.000    0.374    0.374
    SOCI              1.224    0.112   10.928    0.000    0.698    0.698
    TECH              0.698    0.106    6.597    0.000    0.362    0.362
    USEF              0.969    0.123    7.852    0.000    0.425    0.425
    UNRE              0.616    0.118    5.208    0.000    0.266    0.266
  INSE ~~
    PRIV              0.410    0.072    5.667    0.000    0.393    0.393
    OVER              0.544    0.087    6.234    0.000    0.419    0.419
    SAFE              0.527    0.088    6.019    0.000    0.458    0.458
    SOCI              0.436    0.075    5.780    0.000    0.404    0.404
    TECH              0.299    0.069    4.343    0.000    0.252    0.252
    USEF              0.479    0.080    5.999    0.000    0.342    0.342
    UNRE              0.397    0.076    5.193    0.000    0.279    0.279
  PRIV ~~
    OVER              0.697    0.100    6.986    0.000    0.438    0.438
    SAFE              0.972    0.106    9.135    0.000    0.689    0.689
    SOCI              0.747    0.093    8.015    0.000    0.566    0.566
    TECH              0.543    0.089    6.128    0.000    0.374    0.374
    USEF              0.758    0.102    7.416    0.000    0.442    0.442
    UNRE              0.661    0.098    6.728    0.000    0.380    0.380
  OVER ~~
    SAFE              0.929    0.108    8.571    0.000    0.529    0.529
    SOCI              1.381    0.122   11.324    0.000    0.841    0.841
    TECH              1.029    0.120    8.610    0.000    0.570    0.570
    USEF              1.568    0.133   11.745    0.000    0.734    0.734
    UNRE              1.332    0.128   10.447    0.000    0.616    0.616
  SAFE ~~
    SOCI              0.825    0.097    8.517    0.000    0.566    0.566
    TECH              0.680    0.094    7.239    0.000    0.425    0.425
    USEF              0.828    0.101    8.206    0.000    0.437    0.437
    UNRE              0.800    0.104    7.688    0.000    0.416    0.416
  SOCI ~~
```

```
    TECH              0.831    0.100    8.327    0.000    0.555    0.555
    USEF              1.191    0.116   10.273    0.000    0.672    0.672
    UNRE              0.987    0.112    8.831    0.000    0.549    0.549
  TECH ~~
    USEF              1.483    0.132   11.217    0.000    0.760    0.760
    UNRE              1.454    0.118   12.277    0.000    0.735    0.735
  USEF ~~
    UNRE              1.731    0.138   12.508    0.000    0.741    0.741

Variances:
                   Estimate  Std.Err  z-value  P(>|z|)   Std.lv  Std.all
   .COMP1             1.034    0.105    9.848    0.000    1.034    0.330
   .COMP2             1.204    0.116   10.348    0.000    1.204    0.314
   .COMP3             0.700    0.080    8.740    0.000    0.700    0.216
   .COMP4             1.564    0.128   12.235    0.000    1.564    0.398
   .COMP5             1.509    0.121   12.427    0.000    1.509    0.358
   .CONF1             1.262    0.104   12.195    0.000    1.262    0.359
   .CONF2             0.657    0.071    9.215    0.000    0.657    0.200
   .CONF3             0.760    0.100    7.619    0.000    0.760    0.205
   .CONF4             0.366    0.048    7.613    0.000    0.366    0.118
   .CONF5             0.422    0.070    6.012    0.000    0.422    0.129
   .INSE1             1.167    0.122    9.568    0.000    1.167    0.578
   .INSE2             0.144    0.033    4.325    0.000    0.144    0.076
   .INSE3             0.457    0.087    5.253    0.000    0.457    0.226
   .INSE4             0.541    0.082    6.563    0.000    0.541    0.298
   .INSE5             0.433    0.077    5.641    0.000    0.433    0.255
   .PRIV1             1.777    0.121   14.709    0.000    1.777    0.582
   .PRIV2             1.545    0.114   13.556    0.000    1.545    0.484
   .PRIV3             0.758    0.089    8.549    0.000    0.758    0.264
   .PRIV4             0.782    0.094    8.294    0.000    0.782    0.282
   .PRIV5             0.896    0.098    9.163    0.000    0.896    0.315
   .OVER1             1.601    0.134   11.905    0.000    1.601    0.447
   .OVER2             1.509    0.141   10.692    0.000    1.509    0.399
   .OVER3             1.785    0.141   12.697    0.000    1.785    0.502
   .OVER4             1.185    0.104   11.349    0.000    1.185    0.397
   .OVER5             1.349    0.102   13.285    0.000    1.349    0.393
   .SAFE1             0.834    0.101    8.258    0.000    0.834    0.349
   .SAFE2             0.967    0.108    8.943    0.000    0.967    0.389
   .SAFE3             1.244    0.130    9.572    0.000    1.244    0.438
   .SAFE4             1.136    0.135    8.424    0.000    1.136    0.372
   .SAFE5             1.393    0.131   10.660    0.000    1.393    0.517
   .SOCI1             1.830    0.149   12.283    0.000    1.830    0.573
   .SOCI2             1.404    0.117   11.995    0.000    1.404    0.373
```

```
 .SOCI3               1.926    0.133   14.474    0.000    1.926    0.645
 .SOCI4               1.305    0.121   10.814    0.000    1.305    0.372
 .SOCI5               1.144    0.093   12.285    0.000    1.144    0.382
 .TECH1               1.382    0.105   13.179    0.000    1.382    0.456
 .TECH2               1.173    0.116   10.088    0.000    1.173    0.310
 .TECH3               0.635    0.075    8.494    0.000    0.635    0.161
 .TECH4               0.629    0.083    7.535    0.000    0.629    0.162
 .TECH5               1.513    0.152    9.959    0.000    1.513    0.390
 .USEF1               1.211    0.097   12.427    0.000    1.211    0.344
 .USEF2               1.160    0.091   12.726    0.000    1.160    0.343
 .USEF3               1.404    0.112   12.524    0.000    1.404    0.428
 .USEF4               2.019    0.130   15.494    0.000    2.019    0.532
 .USEF5               1.564    0.119   13.148    0.000    1.564    0.463
 .UNRE1               1.102    0.107   10.306    0.000    1.102    0.317
 .UNRE2               0.840    0.100    8.408    0.000    0.840    0.233
 .UNRE3               0.934    0.108    8.611    0.000    0.934    0.282
 .UNRE4               0.642    0.066    9.743    0.000    0.642    0.188
 .UNRE5               1.659    0.129   12.840    0.000    1.659    0.406
  COMP                2.101    0.153   13.721    0.000    1.000    1.000
  CONF                2.257    0.174   12.936    0.000    1.000    1.000
  INSE                0.852    0.138    6.189    0.000    1.000    1.000
  PRIV                1.278    0.146    8.771    0.000    1.000    1.000
  OVER                1.977    0.172   11.488    0.000    1.000    1.000
  SAFE                1.557    0.165    9.454    0.000    1.000    1.000
  SOCI                1.363    0.151    9.005    0.000    1.000    1.000
  TECH                1.650    0.152   10.882    0.000    1.000    1.000
  USEF                2.305    0.164   14.085    0.000    1.000    1.000
  UNRE                2.370    0.170   13.947    0.000    1.000    1.000
```

```
fitmeasures(wholemodelMI, c("cfi", "tli", "rmsea", "srmr", "chisq", "df"))
```

```
  cfi      tli    rmsea     srmr    chisq       df
0.937    0.931    0.049    0.065 2637.978 1120.000
```

Reliability analyses

Cronbach's Alpha, Alpha CIs and McDonald's Omega for subscales

```
nor <- read_sav("onlydss.sav")
```

```
#specify the model

wholemodel <- 'COMP=~COMP1 + COMP2 + COMP3 + COMP4 + COMP5
                    CONF=~CONF1 + CONF2 + CONF3 + CONF4 + CONF5
                     INSE =~ INSE1 + INSE2 + INSE3 + INSE4 + INSE5
                     PRIV =~ PRIV1 + PRIV2 + PRIV3 + PRIV4 + PRIV5
                     OVER =~ OVER1 + OVER2 + OVER3 + OVER4+ OVER5
                     SAFE =~ SAFE1 + SAFE2 + SAFE3 + SAFE4 + SAFE5
                     SOCI=~ SOCI1 + SOCI2 + SOCI3 + SOCI4 + SOCI5
                     TECH =~ TECH1 + TECH2 + TECH3 + TECH4 + TECH5
                     USEF =~ USEF1 + USEF2 + USEF3 + USEF4 + USEF5
                     UNRE =~ UNRE1 + UNRE2 + UNRE3 + UNRE4 + UNRE5
                     DSS =~ COMP + CONF + INSE + PRIV + OVER + SAFE + SOCI + TECH + USEF

fitwholemodel <- lavaan::cfa(wholemodel, data=nor, std.lv=T, estimator='MLR')

semTools::reliability(fitwholemodel)
```

```
            COMP      CONF      INSE      PRIV      OVER      SAFE      SOCI
alpha  0.9223086 0.9525503 0.9212304 0.8971104 0.8802417 0.8918915 0.8458445
omega  0.9231965 0.9534250 0.9259134 0.8978868 0.8809843 0.8923868 0.8507658
omega2 0.9231965 0.9534250 0.9259134 0.8978868 0.8809843 0.8923868 0.8507658
omega3 0.9232257 0.9543371 0.9302551 0.8990237 0.8694252 0.8912015 0.8531568
avevar 0.7070190 0.8040866 0.7169984 0.6378380 0.6029353 0.6248687 0.5371404
            TECH      USEF      UNRE
alpha  0.9217941 0.8768010 0.9234861
omega  0.9258957 0.8793928 0.9246151
omega2 0.9258957 0.8793928 0.9246151
omega3 0.9296674 0.8807950 0.9269358
avevar 0.7166171 0.5953924 0.7106778
```

```
# Calculate Cronbach's alpha CI for Complexity factor
alpha_resultf1<- alpha(nor[, 1:5], na.rm = TRUE)
```

```
alpha_valuef1 <- alpha_resultf1$total$raw_alpha
n.obs <- nrow(nor)
n.var <- 5
alpha_confidence_intervalf1 <- alpha.ci(alpha_valuef1, n.obs, n.var, p.val = 0.05)
print(alpha_confidence_intervalf1)
```

```
  95% confidence boundaries (Feldt)
lower alpha upper
 0.91  0.92  0.93
```

```
# Calculate Cronbach's alpha CI for Conflicts factor
alpha_resultf2<- alpha(nor[, 6:10], na.rm = TRUE)
alpha_valuef2 <- alpha_resultf2$total$raw_alpha
n.obs <- nrow(nor)
n.var <- 5
alpha_confidence_intervalf2 <- alpha.ci(alpha_valuef2, n.obs, n.var, p.val = 0.05)
print(alpha_confidence_intervalf2)
```

```
  95% confidence boundaries (Feldt)
lower alpha upper
 0.95  0.95  0.96
```

```
# Calculate Cronbach's alpha CI for Insecurity factor
alpha_resultf3<- alpha(nor[, 11:15], na.rm = TRUE)
alpha_valuef3 <- alpha_resultf3$total$raw_alpha
n.obs <- nrow(nor)
n.var <- 5
alpha_confidence_intervalf3 <- alpha.ci(alpha_valuef3, n.obs, n.var, p.val = 0.05)
print(alpha_confidence_intervalf3)
```

```
  95% confidence boundaries (Feldt)
lower alpha upper
 0.91  0.92  0.93
```

```
# Calculate Cronbach's alpha CI for Privacy factor
alpha_resultf4<- alpha(nor[, 16:20], na.rm = TRUE)
alpha_valuef4 <- alpha_resultf4$total$raw_alpha
n.obs <- nrow(nor)
```

```r
n.var <- 5
alpha_confidence_intervalf4 <- alpha.ci(alpha_valuef4, n.obs, n.var, p.val = 0.05)
print(alpha_confidence_intervalf4)
```

```
  95% confidence boundaries (Feldt)
lower alpha upper
 0.88   0.9  0.91
```

```r
# Calculate Cronbach's alpha CI for Overload factor
alpha_resultf5<- alpha(nor[, 21:25], na.rm = TRUE)
alpha_valuef5 <- alpha_resultf5$total$raw_alpha
n.obs <- nrow(nor)
n.var <- 5
alpha_confidence_intervalf5 <- alpha.ci(alpha_valuef5, n.obs, n.var, p.val = 0.05)
print(alpha_confidence_intervalf5)
```

```
  95% confidence boundaries (Feldt)
lower alpha upper
 0.86  0.88   0.9
```

```r
# Calculate Cronbach's alpha CI for Safety factor
alpha_resultf6<- alpha(nor[, 26:30], na.rm = TRUE)
alpha_valuef6 <- alpha_resultf6$total$raw_alpha
n.obs <- nrow(nor)
n.var <- 5
alpha_confidence_intervalf6 <- alpha.ci(alpha_valuef6, n.obs, n.var, p.val = 0.05)
print(alpha_confidence_intervalf6)
```

```
  95% confidence boundaries (Feldt)
lower alpha upper
 0.88  0.89  0.91
```

```r
# Calculate Cronbach's alpha CI for Social environment factor
alpha_resultf7<- alpha(nor[, 31:35], na.rm = TRUE)
alpha_valuef7 <- alpha_resultf7$total$raw_alpha
n.obs <- nrow(nor)
n.var <- 5
alpha_confidence_intervalf7 <- alpha.ci(alpha_valuef7, n.obs, n.var, p.val = 0.05)
```

```
print(alpha_confidence_intervalf7)
```

```
 95% confidence boundaries (Feldt)
lower alpha upper
 0.82  0.85  0.87
```

```
# Calculate Cronbach's alpha CI for Technical support factor
alpha_resultf8<- alpha(nor[, 36:40], na.rm = TRUE)
alpha_valuef8 <- alpha_resultf8$total$raw_alpha
n.obs <- nrow(nor)
n.var <- 5
alpha_confidence_intervalf8 <- alpha.ci(alpha_valuef8, n.obs, n.var, p.val = 0.05)
print(alpha_confidence_intervalf8)
```

```
 95% confidence boundaries (Feldt)
lower alpha upper
 0.91  0.92  0.93
```

```
# Calculate Cronbach's alpha CI for Usefulness factor
alpha_resultf9<- alpha(nor[, 41:45], na.rm = TRUE)
alpha_valuef9 <- alpha_resultf9$total$raw_alpha
n.obs <- nrow(nor)
n.var <- 5
alpha_confidence_intervalf9 <- alpha.ci(alpha_valuef9, n.obs, n.var, p.val = 0.05)
print(alpha_confidence_intervalf9)
```

```
 95% confidence boundaries (Feldt)
lower alpha upper
 0.86  0.88  0.89
```

```
# Calculate Cronbach's alpha CI for Unreliability factor
alpha_resultf10<- alpha(all[, 46:50], na.rm = TRUE)
alpha_valuef10 <- alpha_resultf10$total$raw_alpha
n.obs <- nrow(nor)
n.var <- 5
alpha_confidence_intervalf10 <- alpha.ci(alpha_valuef10, n.obs, n.var, p.val = 0.05)
print(alpha_confidence_intervalf10)
```

```
   95% confidence boundaries (Feldt)
 lower alpha upper
  0.91  0.92  0.93
```

McDonald's Omega categorical for subscales

```
mod1f <- 'COMP=~COMP1 + COMP2 + COMP3 + COMP4 + COMP5'
fit1f <- lavaan::cfa(mod1f, data = nor, std.lv=T, ordered = T, estimator='WLSMV')
semTools::reliability(fit1f)
```

```
               COMP
alpha     0.9223086
alpha.ord 0.9390312
omega     0.9373191
omega2    0.9373191
omega3    0.9695436
avevar    0.7881904
```

```
mod2f <- 'CONF=~CONF1 + CONF2 + CONF3 + CONF4 + CONF5 '
fit2f <- lavaan::cfa(mod2f, data = nor, std.lv=T, ordered = T, estimator='WLSMV')
semTools::reliability(fit2f)
```

```
               CONF
alpha     0.9525503
alpha.ord 0.9651136
omega     0.9557280
omega2    0.9557280
omega3    0.9594568
avevar    0.8536757
```

```
mod3f <- 'INSE =~ INSE1 + INSE2 + INSE3 + INSE4 + INSE5'
fit3f <- lavaan::cfa(mod3f, data = nor, std.lv=T, ordered = T, estimator='WLSMV')
semTools::reliability(fit3f)
```

```
               INSE
alpha     0.9212304
alpha.ord 0.9532418
omega     0.9342228
```

```
omega2    0.9342228
omega3    0.9422586
avevar    0.8189410
```

```
mod4f<- 'PRIV =~ PRIV1 + PRIV2 + PRIV3 + PRIV4 + PRIV5'
fit4f <- lavaan::cfa(mod4f, data = nor, std.lv=T, ordered = T, estimator='WLSMV')
semTools::reliability(fit4f)
```

```
               PRIV
alpha     0.8971104
alpha.ord 0.9135530
omega     0.9245980
omega2    0.9245980
omega3    0.9869025
avevar    0.7413698
```

```
mod5f <- 'OVER =~ OVER1 + OVER2 + OVER3 + OVER4+ OVER5'
fit5f <- lavaan::cfa(mod5f, data = nor, std.lv=T, ordered = T, estimator='WLSMV')
semTools::reliability(fit5f)
```

```
               OVER
alpha     0.8802417
alpha.ord 0.8975339
omega     0.8965199
omega2    0.8965199
omega3    0.9187907
avevar    0.6669756
```

```
mod6f <- 'SAFE =~ SAFE1 + SAFE2 + SAFE3 + SAFE4 + SAFE5'
fit6f <- lavaan::cfa(mod6f, data = nor, std.lv=T, ordered = T, estimator='WLSMV')
semTools::reliability(fit6f)
```

```
               SAFE
alpha     0.8918915
alpha.ord 0.9168541
omega     0.9167681
omega2    0.9167681
omega3    0.9609817
avevar    0.7390061
```

```r
mod7f <- 'SOCI=~ SOCI1 + SOCI2 + SOCI3 + SOCI4 + SOCI5'
fit7f <- lavaan::cfa(mod7f, data = nor, std.lv=T, ordered = T, estimator='WLSMV')
semTools::reliability(fit7f)
```

```
               SOCI
alpha     0.8458445
alpha.ord 0.8720752
omega     0.8600985
omega2    0.8600985
omega3    0.8677873
avevar    0.5909105
```

```r
mod8f <- 'TECH =~ TECH1 + TECH2 + TECH3 + TECH4 + TECH5'
fit8f <- lavaan::cfa(mod8f, data = nor, std.lv=T, ordered = T, estimator='WLSMV')
semTools::reliability(fit8f)
```

```
               TECH
alpha     0.9217941
alpha.ord 0.9374415
omega     0.9297820
omega2    0.9297820
omega3    0.9360668
avevar    0.7610845
```

```r
mod9f <- 'USEF =~ USEF1 + USEF2 + USEF3 + USEF4 + USEF5'
fit9f <- lavaan::cfa(mod9f, data = nor, std.lv=T, ordered = T, estimator='WLSMV')
semTools::reliability(fit9f)
```

```
               USEF
alpha     0.8768010
alpha.ord 0.8959749
omega     0.8854424
omega2    0.8854424
omega3    0.8921972
avevar    0.6485025
```

```r
mod10f <- 'UNRE =~ UNRE1 + UNRE2 + UNRE3 + UNRE4 + UNRE5'
fit10f <- lavaan::cfa(mod10f, data = nor, std.lv=T, ordered = T, estimator='WLSMV')
```

```r
semTools::reliability(fit10f)
```

```
               UNRE
alpha     0.9234861
alpha.ord 0.9374552
omega     0.9269894
omega2    0.9269894
omega3    0.9327248
avevar    0.7589063
```

Exploratory Factor Analysis

```r
#bartkett and kmo
bartlett.test(nor)
```

```
	Bartlett test of homogeneity of variances

data:  nor
Bartlett's K-squared = 565.04, df = 49, p-value < 2.2e-16
```

```r
KMO(nor)
```

```
Kaiser-Meyer-Olkin factor adequacy
Call: KMO(r = nor)
Overall MSA =  0.95
MSA for each item = 
COMP1 COMP2 COMP3 COMP4 COMP5 CONF1 CONF2 CONF3 CONF4 CONF5 INSE1 INSE2 INSE3 
 0.95  0.95  0.97  0.93  0.93  0.97  0.94  0.94  0.94  0.95  0.97  0.91  0.94 
INSE4 INSE5 PRIV1 PRIV2 PRIV3 PRIV4 PRIV5 OVER1 OVER2 OVER3 OVER4 OVER5 SAFE1 
 0.89  0.93  0.92  0.92  0.92  0.94  0.94  0.93  0.94  0.96  0.97  0.97  0.93 
SAFE2 SAFE3 SAFE4 SAFE5 SOCI1 SOCI2 SOCI3 SOCI4 SOCI5 TECH1 TECH2 TECH3 TECH4 
 0.92  0.95  0.91  0.92  0.97  0.96  0.95  0.96  0.97  0.98  0.97  0.94  0.95 
TECH5 USEF1 USEF2 USEF3 USEF4 USEF5 UNRE1 UNRE2 UNRE3 UNRE4 UNRE5 
 0.97  0.96  0.95  0.96  0.96  0.98  0.96  0.95  0.94  0.95  0.96 
```

```r
nofactors <- fa.parallel(nor, fm="ml", fa="fa")
```

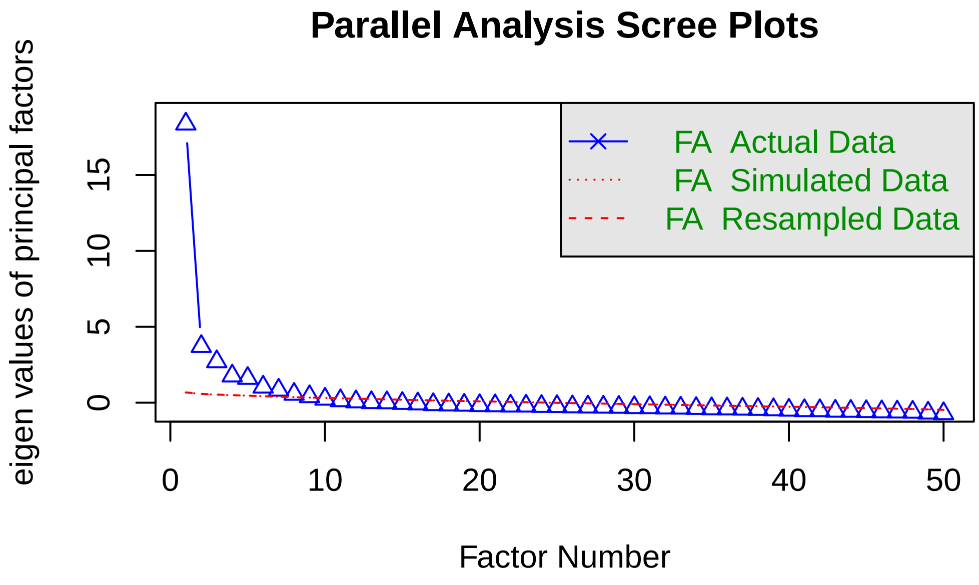


```
Parallel analysis suggests that the number of factors =  9  and the number of components = [illegible]
```

```
sum(nofactors$fa.values > 1.0) #old kaiser criterion
```

```
[1] 6
```

```
sum(nofactors$fa.values > 0.7) #new kaiser criterion
```

```
[1] 7
```

```
ev<- eigen(cor(nor)) #get eigenvalues
ev$value
```

```
 [1] 18.96908403  4.36505867  3.43285496  2.51319986  2.20679202  1.62549918
 [7]  1.53550288  1.15735485  0.97961450  0.83855123  0.77008880  0.68995233
[13]  0.62995930  0.58243235  0.57345945  0.51970848  0.50738560  0.48380699
[19]  0.44180426  0.43237343  0.41137254  0.40848963  0.39566502  0.35225845
[25]  0.33985380  0.33022586  0.30729896  0.30023907  0.28144891  0.27750124
[31]  0.26199064  0.24803133  0.23354230  0.22489594  0.22245364  0.19644480
```

```
[37]  0.19254976  0.18350794  0.17460299  0.16210412  0.15519726  0.15106452
[43]  0.14270434  0.13310231  0.12962238  0.12262696  0.11534691  0.10188053
[49]  0.09758593  0.09190875
```

```
#test the 8 factor solution
solution8 = fa(nor, nfactors=8, rotate="oblimin", fm="ml")
solution8
```

```
Factor Analysis using method =  ml
Call: fa(r = nor, nfactors = 8, rotate = "oblimin", fm = "ml")
Standardized loadings (pattern matrix) based upon correlation matrix
        ML2   ML5   ML6   ML1   ML8   ML3   ML4   ML7   h2    u2 com
COMP1  0.00  0.77  0.04  0.04  0.00  0.05 -0.06  0.05 0.69 0.314 1.0
COMP2  0.00  0.81  0.05  0.03 -0.05  0.01 -0.02  0.09 0.73 0.274 1.0
COMP3  0.01  0.80  0.01  0.03  0.04  0.07 -0.03  0.02 0.74 0.258 1.0
COMP4  0.09  0.72 -0.01  0.01  0.11 -0.06  0.14 -0.01 0.69 0.305 1.2
COMP5  0.06  0.81  0.03 -0.05  0.00 -0.01  0.10 -0.01 0.72 0.284 1.1
CONF1  0.72  0.13  0.00  0.07  0.03  0.04 -0.03  0.03 0.67 0.326 1.1
CONF2  0.94  0.00 -0.01  0.03 -0.03 -0.02 -0.03  0.01 0.85 0.154 1.0
CONF3  0.93 -0.01 -0.02 -0.02 -0.01 -0.03  0.05 -0.01 0.85 0.154 1.0
CONF4  0.93  0.01 -0.02  0.02 -0.02  0.02 -0.01  0.00 0.86 0.136 1.0
CONF5  0.88 -0.02  0.04 -0.05  0.06  0.02  0.00  0.02 0.85 0.150 1.0
INSE1  0.12  0.16  0.10  0.10  0.01  0.54 -0.03 -0.01 0.52 0.475 1.5
INSE2  0.01  0.02 -0.03  0.00  0.00  0.95  0.03 -0.02 0.90 0.097 1.0
INSE3  0.02  0.05  0.06 -0.05  0.00  0.86 -0.01  0.00 0.78 0.224 1.0
INSE4 -0.05 -0.07 -0.03  0.02  0.00  0.88  0.01  0.03 0.75 0.246 1.0
INSE5 -0.02 -0.03 -0.04  0.01  0.01  0.88  0.01  0.04 0.79 0.211 1.0
PRIV1  0.09  0.09  0.12 -0.04  0.02  0.13  0.66 -0.10 0.61 0.392 1.3
PRIV2  0.05  0.04  0.08 -0.01  0.03  0.07  0.77 -0.09 0.67 0.326 1.1
PRIV3 -0.01  0.01 -0.04  0.03 -0.10  0.00  0.73  0.25 0.73 0.275 1.3
PRIV4 -0.02 -0.01 -0.04  0.11  0.02  0.03  0.78  0.03 0.69 0.305 1.1
PRIV5 -0.03  0.02  0.00  0.01  0.05 -0.04  0.68  0.18 0.62 0.384 1.2
OVER1  0.02  0.24  0.06  0.14  0.57  0.00 -0.10  0.04 0.65 0.351 1.6
OVER2 -0.07  0.25  0.04  0.14  0.61  0.07 -0.05  0.02 0.70 0.298 1.5
OVER3  0.06 -0.09 -0.02  0.04  0.68  0.05  0.09  0.02 0.56 0.438 1.1
OVER4  0.05  0.02  0.08 -0.04  0.68  0.08 -0.02  0.05 0.60 0.401 1.1
OVER5  0.10  0.22 -0.05  0.05  0.52  0.00  0.04  0.12 0.58 0.418 1.6
SAFE1  0.03  0.00  0.12 -0.07  0.11  0.04  0.07  0.67 0.64 0.359 1.2
SAFE2 -0.01 -0.03  0.06 -0.02  0.12  0.06  0.03  0.70 0.63 0.374 1.1
SAFE3  0.02 -0.03  0.01  0.03  0.08  0.00  0.13  0.60 0.51 0.489 1.1
SAFE4 -0.01  0.04 -0.03  0.08 -0.04  0.03  0.03  0.83 0.76 0.239 1.0
SAFE5  0.07  0.10  0.03  0.02 -0.08  0.04 -0.01  0.74 0.64 0.356 1.1
```

```
SOCI1  0.08 -0.07  0.08  0.12  0.50  0.13  0.01  0.02 0.48 0.522 1.4
SOCI2  0.33 -0.02 -0.02  0.08  0.45 -0.02  0.17  0.00 0.59 0.410 2.2
SOCI3  0.18 -0.09  0.20 -0.08  0.26  0.13  0.11  0.01 0.30 0.696 4.4
SOCI4  0.16 -0.08  0.10  0.01  0.43 -0.02  0.23  0.14 0.55 0.449 2.3
SOCI5  0.22 -0.13  0.20  0.05  0.39  0.05  0.10  0.10 0.54 0.459 2.9
TECH1  0.13  0.00  0.54  0.09  0.12  0.05 -0.01  0.11 0.62 0.376 1.4
TECH2  0.04  0.03  0.76  0.01  0.05  0.00  0.02  0.04 0.70 0.298 1.0
TECH3 -0.02  0.02  0.94 -0.03 -0.02 -0.04  0.03  0.00 0.85 0.150 1.0
TECH4 -0.02 -0.02  0.90  0.08 -0.03  0.03 -0.01 -0.03 0.85 0.151 1.0
TECH5  0.00  0.04  0.67  0.07 -0.01  0.01 -0.02  0.12 0.61 0.387 1.1
USEF1  0.00  0.31  0.29  0.22  0.16  0.03  0.06 -0.10 0.61 0.395 3.7
USEF2  0.00  0.38  0.29  0.13  0.12  0.05  0.05 -0.03 0.59 0.407 2.5
USEF3 -0.03  0.36  0.20  0.11  0.16 -0.03  0.14 -0.03 0.48 0.520 2.7
USEF4 -0.01  0.23  0.01  0.20  0.36 -0.01  0.12 -0.05 0.45 0.554 2.7
USEF5  0.04  0.33  0.16  0.21  0.09  0.07  0.05 -0.03 0.47 0.526 2.7
UNRE1  0.00  0.06  0.06  0.73  0.01 -0.01  0.11 -0.04 0.69 0.313 1.1
UNRE2 -0.02  0.04 -0.01  0.88 -0.05  0.05  0.04 -0.03 0.78 0.222 1.0
UNRE3  0.08 -0.12  0.06  0.84  0.05 -0.01 -0.03  0.00 0.74 0.255 1.1
UNRE4  0.01  0.00  0.05  0.85 -0.01  0.00 -0.03  0.08 0.81 0.185 1.0
UNRE5 -0.06  0.08  0.01  0.67  0.13 -0.04  0.00  0.07 0.61 0.391 1.2

                       ML2  ML5  ML6  ML1  ML8  ML3  ML4  ML7
SS loadings           4.81 4.74 4.43 4.36 4.30 3.90 3.50 3.30
Proportion Var        0.10 0.09 0.09 0.09 0.09 0.08 0.07 0.07
Cumulative Var        0.10 0.19 0.28 0.37 0.45 0.53 0.60 0.67
Proportion Explained  0.14 0.14 0.13 0.13 0.13 0.12 0.11 0.10
Cumulative Proportion 0.14 0.29 0.42 0.55 0.68 0.80 0.90 1.00

 With factor correlations of
     ML2  ML5  ML6  ML1  ML8  ML3  ML4  ML7
ML2 1.00 0.29 0.34 0.22 0.50 0.22 0.37 0.27
ML5 0.29 1.00 0.52 0.46 0.40 0.24 0.25 0.26
ML6 0.34 0.52 1.00 0.68 0.43 0.23 0.31 0.30
ML1 0.22 0.46 0.68 1.00 0.47 0.25 0.29 0.29
ML8 0.50 0.40 0.43 0.47 1.00 0.31 0.31 0.28
ML3 0.22 0.24 0.23 0.25 0.31 1.00 0.33 0.38
ML4 0.37 0.25 0.31 0.29 0.31 0.33 1.00 0.50
ML7 0.27 0.26 0.30 0.29 0.28 0.38 0.50 1.00

Mean item complexity =  1.5
Test of the hypothesis that 8 factors are sufficient.

df null model =  1225  with the objective function =  45.29 with Chi Square =  24522.93
```

```
df of  the model are 853  and the objective function was  5.49

The root mean square of the residuals (RMSR) is  0.02
The df corrected root mean square of the residuals is  0.03

The harmonic n.obs is  560 with the empirical chi square  857.15  with prob <  0.45
The total n.obs was  560  with Likelihood Chi Square =  2943.84  with prob <  2.1e-227

Tucker Lewis Index of factoring reliability =  0.87
RMSEA index =  0.066  and the 90 % confidence intervals are  0.064 0.069
BIC =  -2453.89
Fit based upon off diagonal values = 1
Measures of factor score adequacy
                                                   ML2  ML5  ML6  ML1  ML8  ML3
Correlation of (regression) scores with factors   0.98 0.96 0.97 0.97 0.94 0.98
Multiple R square of scores with factors          0.96 0.93 0.95 0.94 0.89 0.95
Minimum correlation of possible factor scores     0.93 0.86 0.89 0.87 0.78 0.91
                                                   ML4  ML7
Correlation of (regression) scores with factors   0.95 0.95
Multiple R square of scores with factors          0.90 0.90
Minimum correlation of possible factor scores     0.80 0.80
```

```
#exclude cross loadings and low loadings for the 8 factor solution stpwise
new8factorwithoutSOCI2<- c("SOCI2")
new8f_SOCI2 <- nor[ ,!(names(nor) %in% new8factorwithoutSOCI2)]
solution8f1 = fa(new8f_SOCI2, nfactors=8, rotate="oblimin", fm="ml")
solution8f1
```

```
Factor Analysis using method =  ml
Call: fa(r = new8f_SOCI2, nfactors = 8, rotate = "oblimin", fm = "ml")
Standardized loadings (pattern matrix) based upon correlation matrix
        ML2   ML5   ML6   ML1   ML8   ML3   ML7   ML4   h2    u2 com
COMP1  0.00  0.77  0.04  0.04  0.01  0.05 -0.06  0.05 0.69 0.312 1.0
COMP2  0.00  0.81  0.05  0.03 -0.05  0.01 -0.02  0.09 0.73 0.272 1.0
COMP3  0.01  0.80  0.01  0.03  0.05  0.07 -0.03  0.02 0.74 0.258 1.0
COMP4  0.09  0.71 -0.01  0.01  0.13 -0.06  0.14 -0.02 0.69 0.306 1.2
COMP5  0.06  0.80  0.03 -0.05  0.01 -0.01  0.10 -0.01 0.71 0.285 1.1
CONF1  0.72  0.13  0.00  0.07  0.02  0.04 -0.03  0.03 0.67 0.327 1.1
CONF2  0.94  0.00 -0.01  0.03 -0.02 -0.02 -0.03  0.01 0.85 0.154 1.0
CONF3  0.93 -0.01 -0.02 -0.02 -0.02 -0.03  0.05 -0.01 0.85 0.154 1.0
CONF4  0.93  0.01 -0.02  0.02 -0.02  0.02  0.00  0.00 0.86 0.135 1.0
CONF5  0.89 -0.02  0.04 -0.05  0.05  0.02  0.00  0.01 0.85 0.150 1.0
```

INSE1	0.12	0.15	0.10	0.10	0.02	0.53	-0.03	-0.01	0.52	0.476	1.4
INSE2	0.01	0.02	-0.03	0.00	0.01	0.95	0.03	-0.02	0.90	0.098	1.0
INSE3	0.02	0.05	0.06	-0.06	0.00	0.86	-0.01	0.00	0.78	0.224	1.0
INSE4	-0.04	-0.07	-0.03	0.02	-0.01	0.88	0.01	0.03	0.76	0.245	1.0
INSE5	-0.02	-0.02	-0.04	0.01	0.01	0.88	0.01	0.04	0.79	0.211	1.0
PRIV1	0.09	0.09	0.11	-0.04	0.03	0.12	0.67	-0.10	0.62	0.384	1.3
PRIV2	0.05	0.03	0.07	0.00	0.03	0.07	0.78	-0.09	0.68	0.320	1.1
PRIV3	-0.01	0.01	-0.04	0.03	-0.09	0.00	0.73	0.25	0.72	0.278	1.3
PRIV4	-0.01	-0.01	-0.04	0.11	0.02	0.02	0.78	0.04	0.69	0.311	1.1
PRIV5	-0.02	0.02	0.00	0.01	0.05	-0.04	0.68	0.18	0.61	0.388	1.2
OVER1	0.02	0.21	0.05	0.12	0.62	-0.01	-0.09	0.03	0.67	0.325	1.4
OVER2	-0.07	0.22	0.04	0.13	0.65	0.06	-0.04	0.02	0.72	0.277	1.4
OVER3	0.08	-0.12	-0.03	0.04	0.69	0.04	0.11	0.01	0.56	0.436	1.2
OVER4	0.06	0.00	0.07	-0.05	0.70	0.07	0.00	0.04	0.61	0.395	1.1
OVER5	0.11	0.20	-0.05	0.05	0.52	0.00	0.05	0.12	0.57	0.426	1.6
SAFE1	0.03	-0.01	0.12	-0.07	0.13	0.04	0.09	0.65	0.63	0.368	1.2
SAFE2	-0.01	-0.03	0.07	-0.02	0.14	0.06	0.05	0.68	0.62	0.382	1.1
SAFE3	0.02	-0.04	0.02	0.02	0.09	0.00	0.14	0.59	0.51	0.491	1.2
SAFE4	0.00	0.05	-0.03	0.08	-0.04	0.03	0.03	0.83	0.77	0.227	1.0
SAFE5	0.07	0.10	0.03	0.02	-0.09	0.05	-0.02	0.75	0.66	0.341	1.1
SOCI1	0.10	-0.08	0.07	0.12	0.48	0.13	0.01	0.02	0.46	0.537	1.5
SOCI3	0.20	-0.09	0.19	-0.07	0.23	0.13	0.12	0.01	0.30	0.704	4.8
SOCI4	0.19	-0.08	0.09	0.03	0.39	-0.02	0.23	0.14	0.53	0.474	2.8
SOCI5	0.24	-0.14	0.19	0.06	0.36	0.05	0.10	0.10	0.53	0.472	3.3
TECH1	0.13	0.00	0.54	0.09	0.12	0.04	0.00	0.10	0.62	0.375	1.4
TECH2	0.04	0.03	0.76	0.01	0.05	0.00	0.02	0.04	0.70	0.298	1.0
TECH3	-0.02	0.02	0.95	-0.03	-0.02	-0.04	0.03	0.00	0.85	0.150	1.0
TECH4	-0.02	-0.01	0.90	0.08	-0.03	0.03	-0.01	-0.03	0.85	0.152	1.0
TECH5	0.00	0.04	0.68	0.07	-0.01	0.01	-0.03	0.12	0.61	0.386	1.1
USEF1	0.01	0.30	0.29	0.23	0.14	0.03	0.06	-0.10	0.60	0.398	3.6
USEF2	0.01	0.38	0.29	0.13	0.11	0.05	0.05	-0.03	0.59	0.409	2.5
USEF3	-0.02	0.36	0.20	0.11	0.15	-0.03	0.14	-0.03	0.48	0.522	2.6
USEF4	0.01	0.22	0.00	0.21	0.36	-0.01	0.13	-0.06	0.44	0.559	2.7
USEF5	0.05	0.33	0.15	0.22	0.08	0.07	0.05	-0.03	0.47	0.526	2.7
UNRE1	0.01	0.06	0.06	0.73	0.01	-0.01	0.11	-0.04	0.69	0.313	1.1
UNRE2	-0.02	0.04	-0.01	0.88	-0.05	0.05	0.04	-0.03	0.78	0.222	1.0
UNRE3	0.08	-0.12	0.05	0.84	0.04	-0.01	-0.03	0.00	0.74	0.256	1.1
UNRE4	0.01	0.00	0.05	0.85	0.00	0.00	-0.02	0.08	0.81	0.185	1.0
UNRE5	-0.06	0.07	0.01	0.66	0.14	-0.04	0.00	0.07	0.61	0.392	1.2

	ML2	ML5	ML6	ML1	ML8	ML3	ML7	ML4
SS loadings	4.70	4.64	4.40	4.32	4.04	3.89	3.49	3.23
Proportion Var	0.10	0.09	0.09	0.09	0.08	0.08	0.07	0.07

```
Cumulative Var        0.10 0.19 0.28 0.37 0.45 0.53 0.60 0.67
Proportion Explained  0.14 0.14 0.13 0.13 0.12 0.12 0.11 0.10
Cumulative Proportion 0.14 0.29 0.42 0.55 0.68 0.79 0.90 1.00

 With factor correlations of
     ML2  ML5  ML6  ML1  ML8  ML3  ML7  ML4
ML2 1.00 0.29 0.35 0.22 0.49 0.23 0.37 0.27
ML5 0.29 1.00 0.52 0.45 0.42 0.23 0.25 0.25
ML6 0.35 0.52 1.00 0.68 0.45 0.23 0.32 0.29
ML1 0.22 0.45 0.68 1.00 0.49 0.25 0.29 0.28
ML8 0.49 0.42 0.45 0.49 1.00 0.32 0.31 0.28
ML3 0.23 0.23 0.23 0.25 0.32 1.00 0.34 0.37
ML7 0.37 0.25 0.32 0.29 0.31 0.34 1.00 0.50
ML4 0.27 0.25 0.29 0.28 0.28 0.37 0.50 1.00

Mean item complexity =  1.5
Test of the hypothesis that 8 factors are sufficient.

df null model =  1176  with the objective function =  44.25 with Chi Square =  23976.59
df of  the model are 812  and the objective function was  5.27

The root mean square of the residuals (RMSR) is  0.03
The df corrected root mean square of the residuals is  0.03

The harmonic n.obs is  560 with the empirical chi square  827.6  with prob <  0.34
The total n.obs was  560  with Likelihood Chi Square =  2825.92  with prob <  3e-220

Tucker Lewis Index of factoring reliability =  0.871
RMSEA index =  0.067  and the 90 % confidence intervals are  0.064 0.069
BIC =  -2312.36
Fit based upon off diagonal values = 1
Measures of factor score adequacy
                                                   ML2  ML5  ML6  ML1  ML8  ML3
Correlation of (regression) scores with factors   0.98 0.96 0.97 0.97 0.94 0.98
Multiple R square of scores with factors          0.96 0.93 0.95 0.94 0.89 0.95
Minimum correlation of possible factor scores     0.93 0.86 0.89 0.87 0.78 0.91
                                                   ML7  ML4
Correlation of (regression) scores with factors   0.95 0.95
Multiple R square of scores with factors          0.90 0.90
Minimum correlation of possible factor scores     0.80 0.80
```

```r
new8factorwithoutSOCI3<- c("SOCI2", "SOCI3")
new8f_SOCI3 <- nor[ ,!(names(nor) %in% new8factorwithoutSOCI3)]
solution8f2 = fa(new8f_SOCI3, nfactors=8, rotate="oblimin", fm="ml")
solution8f2
```

```
Factor Analysis using method =  ml
Call: fa(r = new8f_SOCI3, nfactors = 8, rotate = "oblimin", fm = "ml")
Standardized loadings (pattern matrix) based upon correlation matrix
        ML2   ML5   ML1   ML6   ML8   ML3   ML7   ML4   h2    u2 com
COMP1  0.00  0.77  0.04  0.04  0.01  0.05 -0.06  0.05 0.69 0.311 1.0
COMP2  0.00  0.81  0.03  0.05 -0.04  0.01 -0.02  0.09 0.73 0.271 1.0
COMP3  0.01  0.80  0.04  0.01  0.04  0.07 -0.03  0.02 0.74 0.255 1.0
COMP4  0.08  0.70  0.00 -0.01  0.13 -0.06  0.14 -0.02 0.69 0.307 1.2
COMP5  0.06  0.80 -0.05  0.03  0.01 -0.01  0.10 -0.01 0.71 0.286 1.1
CONF1  0.72  0.13  0.07  0.00  0.03  0.04 -0.03  0.03 0.67 0.327 1.1
CONF2  0.94  0.00  0.03 -0.01 -0.02 -0.02 -0.03  0.01 0.85 0.153 1.0
CONF3  0.93 -0.01 -0.02 -0.02 -0.02 -0.03  0.05 -0.01 0.85 0.154 1.0
CONF4  0.93  0.01  0.02 -0.02 -0.01  0.02  0.00  0.00 0.86 0.136 1.0
CONF5  0.89 -0.02 -0.05  0.04  0.05  0.02  0.00  0.01 0.85 0.151 1.0
INSE1  0.11  0.15  0.09  0.11  0.04  0.53 -0.02 -0.01 0.52 0.476 1.4
INSE2  0.01  0.02  0.00 -0.03  0.01  0.95  0.03 -0.02 0.90 0.097 1.0
INSE3  0.02  0.04 -0.06  0.07  0.01  0.86 -0.01  0.00 0.78 0.224 1.0
INSE4 -0.04 -0.07  0.02 -0.03 -0.02  0.88  0.01  0.03 0.76 0.245 1.0
INSE5 -0.02 -0.02  0.01 -0.04  0.01  0.88  0.01  0.04 0.79 0.210 1.0
PRIV1  0.10  0.08 -0.04  0.11  0.03  0.12  0.67 -0.11 0.62 0.384 1.3
PRIV2  0.06  0.03  0.00  0.07  0.02  0.07  0.78 -0.09 0.68 0.319 1.1
PRIV3 -0.01  0.01  0.03 -0.03 -0.09  0.00  0.73  0.25 0.72 0.279 1.3
PRIV4 -0.01 -0.01  0.11 -0.04  0.02  0.02  0.78  0.04 0.69 0.311 1.1
PRIV5 -0.03  0.02  0.00  0.00  0.05 -0.04  0.68  0.18 0.61 0.387 1.2
OVER1  0.01  0.19  0.10  0.06  0.66 -0.02 -0.08  0.02 0.69 0.305 1.3
OVER2 -0.07  0.20  0.11  0.04  0.69  0.06 -0.03  0.01 0.74 0.258 1.3
OVER3  0.09 -0.13  0.03 -0.03  0.68  0.04  0.12  0.01 0.56 0.445 1.2
OVER4  0.07 -0.02 -0.05  0.07  0.70  0.07  0.00  0.03 0.60 0.399 1.1
OVER5  0.12  0.19  0.05 -0.05  0.51  0.00  0.06  0.11 0.57 0.431 1.6
SAFE1  0.03 -0.01 -0.07  0.12  0.13  0.04  0.09  0.65 0.63 0.367 1.2
SAFE2 -0.01 -0.04 -0.03  0.07  0.14  0.06  0.05  0.68 0.62 0.381 1.1
SAFE3  0.02 -0.04  0.03  0.02  0.09  0.00  0.14  0.59 0.51 0.491 1.2
SAFE4 -0.01  0.05  0.08 -0.03 -0.04  0.03  0.03  0.83 0.77 0.229 1.0
SAFE5  0.07  0.11  0.02  0.03 -0.09  0.05 -0.02  0.75 0.66 0.341 1.1
SOCI1  0.11 -0.09  0.12  0.07  0.48  0.13  0.02  0.02 0.46 0.539 1.5
SOCI4  0.20 -0.08  0.04  0.08  0.36 -0.02  0.23  0.14 0.51 0.488 3.1
SOCI5  0.26 -0.13  0.07  0.18  0.34  0.05  0.10  0.11 0.51 0.488 3.5
```

```
TECH1  0.13  0.00  0.10  0.53  0.12  0.05  0.00  0.10 0.62 0.378 1.4
TECH2  0.05  0.02  0.00  0.76  0.06  0.00  0.02  0.04 0.70 0.297 1.0
TECH3 -0.02  0.02 -0.04  0.95 -0.02 -0.04  0.03  0.00 0.85 0.147 1.0
TECH4 -0.02 -0.02  0.08  0.90 -0.03  0.03 -0.01 -0.03 0.85 0.152 1.0
TECH5  0.00  0.04  0.07  0.68 -0.01  0.01 -0.03  0.12 0.61 0.387 1.1
USEF1  0.02  0.30  0.23  0.28  0.14  0.03  0.06 -0.10 0.60 0.398 3.7
USEF2  0.01  0.38  0.14  0.28  0.11  0.05  0.05 -0.03 0.59 0.409 2.5
USEF3 -0.01  0.36  0.12  0.19  0.14 -0.03  0.14 -0.03 0.48 0.523 2.6
USEF4  0.01  0.21  0.21  0.00  0.35 -0.01  0.13 -0.06 0.44 0.563 2.8
USEF5  0.06  0.33  0.23  0.14  0.07  0.07  0.05 -0.03 0.47 0.525 2.6
UNRE1  0.00  0.06  0.72  0.06  0.02 -0.01  0.11 -0.04 0.69 0.314 1.1
UNRE2 -0.02  0.05  0.88 -0.01 -0.05  0.05  0.04 -0.03 0.78 0.224 1.0
UNRE3  0.08 -0.11  0.84  0.05  0.04 -0.01 -0.03  0.00 0.75 0.254 1.1
UNRE4  0.01  0.00  0.85  0.05  0.00  0.00 -0.03  0.08 0.82 0.184 1.0
UNRE5 -0.06  0.07  0.66  0.01  0.15 -0.04  0.00  0.07 0.61 0.392 1.2

                       ML2  ML5  ML1  ML6  ML8  ML3  ML7  ML4
SS loadings           4.64 4.61 4.32 4.32 3.98 3.84 3.47 3.22
Proportion Var        0.10 0.10 0.09 0.09 0.08 0.08 0.07 0.07
Cumulative Var        0.10 0.19 0.28 0.37 0.46 0.54 0.61 0.68
Proportion Explained  0.14 0.14 0.13 0.13 0.12 0.12 0.11 0.10
Cumulative Proportion 0.14 0.29 0.42 0.55 0.68 0.79 0.90 1.00

 With factor correlations of
     ML2  ML5  ML1  ML6  ML8  ML3  ML7  ML4
ML2 1.00 0.29 0.22 0.34 0.49 0.22 0.37 0.27
ML5 0.29 1.00 0.45 0.52 0.44 0.23 0.25 0.25
ML1 0.22 0.45 1.00 0.68 0.50 0.25 0.29 0.28
ML6 0.34 0.52 0.68 1.00 0.46 0.23 0.32 0.29
ML8 0.49 0.44 0.50 0.46 1.00 0.32 0.31 0.28
ML3 0.22 0.23 0.25 0.23 0.32 1.00 0.34 0.37
ML7 0.37 0.25 0.29 0.32 0.31 0.34 1.00 0.50
ML4 0.27 0.25 0.28 0.29 0.28 0.37 0.50 1.00

Mean item complexity =  1.4
Test of the hypothesis that 8 factors are sufficient.

df null model =  1128  with the objective function =  43.66 with Chi Square =  23669.39
df of  the model are 772  and the objective function was  5

The root mean square of the residuals (RMSR) is  0.02
The df corrected root mean square of the residuals is  0.03
```

```
The harmonic n.obs is  560 with the empirical chi square  729.42  with prob <  0.86
The total n.obs was  560  with Likelihood Chi Square =  2686.02  with prob <  2e-209

Tucker Lewis Index of factoring reliability =  0.875
RMSEA index =  0.067  and the 90 % confidence intervals are  0.064 0.069
BIC =  -2199.15
Fit based upon off diagonal values = 1
Measures of factor score adequacy
                                                   ML2  ML5  ML1  ML6  ML8  ML3
Correlation of (regression) scores with factors   0.98 0.96 0.97 0.97 0.95 0.98
Multiple R square of scores with factors          0.96 0.93 0.94 0.95 0.89 0.95
Minimum correlation of possible factor scores     0.93 0.86 0.87 0.89 0.79 0.91
                                                   ML7  ML4
Correlation of (regression) scores with factors   0.95 0.95
Multiple R square of scores with factors          0.90 0.90
Minimum correlation of possible factor scores     0.80 0.80
```

```
new8factorwithoutUSEF1<- c("SOCI2", "SOCI3", "USEF1")
new8f_USEF1 <- nor[ ,!(names(nor) %in% new8factorwithoutUSEF1)]
solution8f3 = fa(new8f_USEF1, nfactors=8, rotate="oblimin", fm="ml")
solution8f3
```

```
Factor Analysis using method =  ml
Call: fa(r = new8f_USEF1, nfactors = 8, rotate = "oblimin", fm = "ml")
Standardized loadings (pattern matrix) based upon correlation matrix
        ML2   ML5   ML1   ML6   ML8   ML3   ML7   ML4   h2    u2 com
COMP1  0.00  0.77  0.04  0.04  0.01  0.05 -0.05  0.04 0.69 0.307 1.0
COMP2  0.00  0.81  0.03  0.05 -0.04  0.01 -0.02  0.09 0.73 0.268 1.0
COMP3  0.01  0.80  0.04  0.01  0.05  0.06 -0.03  0.02 0.74 0.257 1.0
COMP4  0.08  0.71  0.01 -0.01  0.13 -0.06  0.15 -0.02 0.70 0.301 1.2
COMP5  0.06  0.80 -0.04  0.03  0.01 -0.01  0.10 -0.01 0.72 0.283 1.1
CONF1  0.72  0.13  0.07  0.00  0.03  0.04 -0.03  0.03 0.67 0.327 1.1
CONF2  0.94  0.00  0.03 -0.01 -0.02 -0.02 -0.03  0.01 0.85 0.153 1.0
CONF3  0.93 -0.01 -0.02 -0.02 -0.02 -0.03  0.05 -0.01 0.85 0.153 1.0
CONF4  0.93  0.01  0.02 -0.02 -0.01  0.02  0.00  0.00 0.86 0.136 1.0
CONF5  0.89 -0.02 -0.05  0.04  0.05  0.02  0.00  0.02 0.85 0.151 1.0
INSE1  0.11  0.15  0.09  0.11  0.04  0.53 -0.03 -0.01 0.52 0.476 1.4
INSE2  0.01  0.02  0.00 -0.03  0.01  0.94  0.03 -0.02 0.90 0.097 1.0
INSE3  0.02  0.05 -0.06  0.07  0.01  0.86 -0.01  0.00 0.78 0.224 1.0
INSE4 -0.04 -0.07  0.02 -0.03 -0.02  0.88  0.01  0.03 0.76 0.245 1.0
INSE5 -0.01 -0.02  0.02 -0.04  0.01  0.88  0.01  0.04 0.79 0.210 1.0
```

PRIV1	0.09	0.09	-0.04	0.11	0.02	0.12	0.68	-0.11	0.62	0.377	1.3
PRIV2	0.05	0.04	0.00	0.07	0.02	0.06	0.78	-0.10	0.69	0.312	1.1
PRIV3	-0.01	0.01	0.03	-0.03	-0.08	0.00	0.72	0.25	0.72	0.283	1.3
PRIV4	-0.01	-0.01	0.11	-0.04	0.02	0.02	0.77	0.05	0.68	0.316	1.1
PRIV5	-0.02	0.02	0.00	0.00	0.05	-0.04	0.67	0.18	0.61	0.392	1.2
OVER1	0.01	0.19	0.09	0.06	0.67	-0.02	-0.08	0.02	0.70	0.296	1.3
OVER2	-0.08	0.20	0.10	0.04	0.70	0.05	-0.04	0.01	0.75	0.250	1.3
OVER3	0.09	-0.13	0.04	-0.03	0.68	0.04	0.12	0.01	0.55	0.448	1.2
OVER4	0.07	-0.02	-0.05	0.08	0.69	0.07	0.01	0.03	0.60	0.402	1.1
OVER5	0.12	0.19	0.05	-0.05	0.51	0.00	0.06	0.11	0.57	0.435	1.6
SAFE1	0.03	-0.01	-0.07	0.12	0.13	0.04	0.10	0.63	0.62	0.381	1.3
SAFE2	-0.01	-0.04	-0.03	0.07	0.14	0.06	0.06	0.66	0.60	0.395	1.2
SAFE3	0.02	-0.04	0.02	0.02	0.09	0.00	0.14	0.58	0.50	0.498	1.2
SAFE4	-0.01	0.04	0.07	-0.03	-0.03	0.03	0.03	0.85	0.79	0.213	1.0
SAFE5	0.07	0.10	0.02	0.03	-0.09	0.05	-0.03	0.77	0.67	0.326	1.1
SOCI1	0.11	-0.09	0.12	0.07	0.48	0.13	0.02	0.02	0.46	0.540	1.5
SOCI4	0.20	-0.08	0.04	0.08	0.36	-0.02	0.23	0.15	0.51	0.490	3.1
SOCI5	0.26	-0.13	0.07	0.17	0.33	0.05	0.10	0.10	0.51	0.489	3.6
TECH1	0.14	0.00	0.10	0.53	0.12	0.05	0.00	0.10	0.62	0.381	1.4
TECH2	0.05	0.02	0.01	0.76	0.06	0.00	0.02	0.04	0.70	0.298	1.0
TECH3	-0.02	0.02	-0.04	0.95	-0.02	-0.04	0.03	-0.01	0.86	0.143	1.0
TECH4	-0.02	-0.01	0.08	0.90	-0.03	0.03	-0.01	-0.04	0.85	0.151	1.0
TECH5	0.00	0.04	0.07	0.68	-0.01	0.01	-0.03	0.12	0.61	0.386	1.1
USEF2	0.02	0.36	0.14	0.28	0.11	0.05	0.04	-0.01	0.57	0.429	2.5
USEF3	-0.01	0.35	0.12	0.19	0.14	-0.03	0.13	-0.02	0.47	0.530	2.6
USEF4	0.02	0.21	0.22	0.00	0.34	-0.01	0.12	-0.05	0.43	0.573	2.8
USEF5	0.06	0.33	0.23	0.14	0.07	0.07	0.05	-0.03	0.47	0.530	2.6
UNRE1	0.00	0.06	0.72	0.06	0.02	-0.01	0.11	-0.04	0.68	0.317	1.1
UNRE2	-0.03	0.05	0.88	-0.01	-0.05	0.05	0.04	-0.04	0.78	0.222	1.0
UNRE3	0.08	-0.11	0.84	0.05	0.04	-0.01	-0.03	-0.01	0.75	0.252	1.1
UNRE4	0.01	0.01	0.85	0.05	0.00	0.00	-0.02	0.07	0.82	0.182	1.0
UNRE5	-0.06	0.07	0.66	0.01	0.15	-0.04	0.00	0.07	0.61	0.394	1.2

	ML2	ML5	ML1	ML6	ML8	ML3	ML7	ML4
SS loadings	4.63	4.41	4.19	4.13	3.92	3.83	3.43	3.24
Proportion Var	0.10	0.09	0.09	0.09	0.08	0.08	0.07	0.07
Cumulative Var	0.10	0.19	0.28	0.37	0.45	0.53	0.61	0.68
Proportion Explained	0.15	0.14	0.13	0.13	0.12	0.12	0.11	0.10
Cumulative Proportion	0.15	0.28	0.42	0.55	0.67	0.79	0.90	1.00

With factor correlations of

	ML2	ML5	ML1	ML6	ML8	ML3	ML7	ML4
ML2	1.00	0.29	0.22	0.34	0.48	0.22	0.37	0.27

```
ML5 0.29 1.00 0.44 0.52 0.44 0.23 0.25 0.26
ML1 0.22 0.44 1.00 0.68 0.50 0.25 0.29 0.29
ML6 0.34 0.52 0.68 1.00 0.46 0.23 0.31 0.30
ML8 0.48 0.44 0.50 0.46 1.00 0.32 0.31 0.28
ML3 0.22 0.23 0.25 0.23 0.32 1.00 0.34 0.37
ML7 0.37 0.25 0.29 0.31 0.31 0.34 1.00 0.50
ML4 0.27 0.26 0.29 0.30 0.28 0.37 0.50 1.00

Mean item complexity =  1.4
Test of the hypothesis that 8 factors are sufficient.

df null model =  1081  with the objective function =  42.35 with Chi Square =  22977.37
df of  the model are 733  and the objective function was  4.58

The root mean square of the residuals (RMSR) is  0.02
The df corrected root mean square of the residuals is  0.03

The harmonic n.obs is  560 with the empirical chi square  656.95  with prob <  0.98
The total n.obs was  560  with Likelihood Chi Square =  2458.48  with prob <  7.6e-185

Tucker Lewis Index of factoring reliability =  0.883
RMSEA index =  0.065  and the 90 % confidence intervals are  0.062 0.068
BIC =  -2179.9
Fit based upon off diagonal values = 1
Measures of factor score adequacy
                                                   ML2  ML5  ML1  ML6  ML8  ML3
Correlation of (regression) scores with factors   0.98 0.96 0.97 0.97 0.95 0.98
Multiple R square of scores with factors          0.96 0.93 0.94 0.95 0.89 0.95
Minimum correlation of possible factor scores     0.92 0.85 0.87 0.89 0.79 0.91
                                                   ML7  ML4
Correlation of (regression) scores with factors   0.95 0.95
Multiple R square of scores with factors          0.90 0.90
Minimum correlation of possible factor scores     0.80 0.80
```

```r
new8factorwithoutUSEF2<- c("SOCI2", "SOCI3", "USEF1", "USEF2")
new8f_USEF2 <- nor[ ,!(names(nor) %in% new8factorwithoutUSEF2)]
solution8f4 = fa(new8f_USEF2, nfactors=8, rotate="oblimin", fm="ml")
solution8f4
```

```
Factor Analysis using method =  ml
Call: fa(r = new8f_USEF2, nfactors = 8, rotate = "oblimin", fm = "ml")
```

Standardized loadings (pattern matrix) based upon correlation matrix

	ML2	ML5	ML1	ML6	ML8	ML3	ML7	ML4	h2	u2	com
COMP1	0.00	0.78	0.04	0.04	0.01	0.05	-0.06	0.04	0.70	0.302	1.0
COMP2	0.00	0.81	0.03	0.06	-0.04	0.01	-0.02	0.09	0.74	0.264	1.0
COMP3	0.01	0.79	0.04	0.01	0.05	0.06	-0.03	0.02	0.74	0.261	1.0
COMP4	0.08	0.71	0.01	-0.01	0.13	-0.06	0.15	-0.03	0.70	0.297	1.2
COMP5	0.06	0.80	-0.04	0.03	0.01	-0.01	0.10	-0.01	0.72	0.283	1.1
CONF1	0.72	0.13	0.07	0.01	0.03	0.04	-0.03	0.03	0.67	0.327	1.1
CONF2	0.94	0.00	0.02	-0.01	-0.02	-0.02	-0.03	0.01	0.85	0.153	1.0
CONF3	0.93	-0.01	-0.02	-0.02	-0.02	-0.03	0.05	-0.01	0.85	0.153	1.0
CONF4	0.93	0.01	0.02	-0.02	-0.01	0.02	0.00	0.00	0.86	0.136	1.0
CONF5	0.89	-0.02	-0.05	0.04	0.05	0.02	0.00	0.02	0.85	0.151	1.0
INSE1	0.11	0.15	0.09	0.11	0.04	0.53	-0.03	-0.01	0.52	0.477	1.4
INSE2	0.01	0.02	0.00	-0.03	0.01	0.94	0.03	-0.02	0.90	0.097	1.0
INSE3	0.02	0.05	-0.06	0.07	0.01	0.86	-0.01	0.00	0.78	0.224	1.0
INSE4	-0.04	-0.07	0.02	-0.03	-0.02	0.88	0.01	0.03	0.76	0.245	1.0
INSE5	-0.01	-0.02	0.02	-0.04	0.01	0.88	0.01	0.04	0.79	0.210	1.0
PRIV1	0.09	0.09	-0.04	0.11	0.02	0.12	0.68	-0.11	0.62	0.375	1.3
PRIV2	0.05	0.04	0.00	0.07	0.02	0.06	0.78	-0.10	0.69	0.310	1.1
PRIV3	-0.01	0.01	0.03	-0.03	-0.08	0.00	0.72	0.25	0.72	0.284	1.3
PRIV4	-0.01	-0.01	0.11	-0.04	0.02	0.02	0.77	0.05	0.68	0.317	1.1
PRIV5	-0.02	0.02	0.00	0.00	0.05	-0.04	0.67	0.18	0.61	0.393	1.2
OVER1	0.01	0.19	0.09	0.06	0.67	-0.02	-0.08	0.02	0.71	0.292	1.3
OVER2	-0.08	0.19	0.10	0.05	0.70	0.05	-0.04	0.01	0.75	0.249	1.3
OVER3	0.09	-0.13	0.04	-0.03	0.67	0.04	0.12	0.01	0.55	0.448	1.2
OVER4	0.07	-0.01	-0.05	0.08	0.69	0.08	0.01	0.02	0.60	0.402	1.1
OVER5	0.12	0.19	0.05	-0.05	0.51	0.00	0.06	0.11	0.57	0.434	1.6
SAFE1	0.03	-0.01	-0.08	0.12	0.13	0.05	0.11	0.63	0.62	0.384	1.3
SAFE2	-0.01	-0.04	-0.03	0.07	0.14	0.06	0.06	0.66	0.60	0.398	1.2
SAFE3	0.02	-0.04	0.02	0.02	0.09	0.00	0.14	0.58	0.50	0.499	1.2
SAFE4	-0.01	0.04	0.07	-0.03	-0.03	0.03	0.03	0.85	0.79	0.210	1.0
SAFE5	0.07	0.10	0.02	0.03	-0.08	0.05	-0.03	0.77	0.68	0.324	1.1
SOCI1	0.11	-0.10	0.12	0.07	0.48	0.13	0.02	0.02	0.46	0.541	1.5
SOCI4	0.21	-0.08	0.05	0.08	0.35	-0.02	0.23	0.15	0.51	0.490	3.2
SOCI5	0.26	-0.14	0.08	0.17	0.33	0.05	0.10	0.10	0.51	0.490	3.6
TECH1	0.14	-0.01	0.10	0.53	0.12	0.05	0.00	0.10	0.62	0.383	1.4
TECH2	0.05	0.02	0.01	0.76	0.06	0.00	0.02	0.03	0.70	0.298	1.0
TECH3	-0.02	0.02	-0.04	0.96	-0.02	-0.04	0.03	-0.01	0.86	0.138	1.0
TECH4	-0.02	-0.01	0.08	0.90	-0.03	0.03	-0.01	-0.04	0.85	0.152	1.0
TECH5	0.00	0.04	0.07	0.67	-0.01	0.01	-0.03	0.12	0.61	0.387	1.1
USEF3	-0.01	0.34	0.13	0.19	0.14	-0.03	0.13	-0.02	0.46	0.540	2.7
USEF4	0.02	0.20	0.22	-0.01	0.34	-0.01	0.12	-0.04	0.42	0.577	2.8
USEF5	0.06	0.32	0.23	0.14	0.07	0.07	0.05	-0.02	0.47	0.535	2.7

```
UNRE1  0.00  0.06  0.72  0.06  0.02 -0.01  0.11 -0.04 0.68 0.319 1.1
UNRE2 -0.03  0.05  0.87 -0.01 -0.04  0.05  0.04 -0.04 0.78 0.222 1.0
UNRE3  0.08 -0.10  0.84  0.05  0.04 -0.01 -0.02 -0.01 0.75 0.250 1.1
UNRE4  0.00  0.01  0.85  0.05  0.00  0.00 -0.02  0.07 0.82 0.182 1.0
UNRE5 -0.06  0.07  0.66  0.01  0.15 -0.04  0.00  0.07 0.61 0.394 1.2

                       ML2  ML5  ML1  ML6  ML8  ML3  ML7  ML4
SS loadings           4.62 4.16 4.12 3.97 3.86 3.82 3.41 3.23
Proportion Var        0.10 0.09 0.09 0.09 0.08 0.08 0.07 0.07
Cumulative Var        0.10 0.19 0.28 0.37 0.45 0.53 0.61 0.68
Proportion Explained  0.15 0.13 0.13 0.13 0.12 0.12 0.11 0.10
Cumulative Proportion 0.15 0.28 0.41 0.54 0.66 0.79 0.90 1.00

 With factor correlations of
     ML2  ML5  ML1  ML6  ML8  ML3  ML7  ML4
ML2 1.00 0.29 0.22 0.35 0.48 0.22 0.37 0.27
ML5 0.29 1.00 0.44 0.52 0.43 0.23 0.25 0.26
ML1 0.22 0.44 1.00 0.68 0.50 0.25 0.29 0.29
ML6 0.35 0.52 0.68 1.00 0.46 0.23 0.31 0.30
ML8 0.48 0.43 0.50 0.46 1.00 0.32 0.31 0.28
ML3 0.22 0.23 0.25 0.23 0.32 1.00 0.34 0.37
ML7 0.37 0.25 0.29 0.31 0.31 0.34 1.00 0.50
ML4 0.27 0.26 0.29 0.30 0.28 0.37 0.50 1.00

Mean item complexity =  1.3
Test of the hypothesis that 8 factors are sufficient.

df null model =  1035  with the objective function =  41.32 with Chi Square =  22431.04
df of  the model are 695  and the objective function was  4.35

The root mean square of the residuals (RMSR) is  0.02
The df corrected root mean square of the residuals is  0.03

The harmonic n.obs is  560 with the empirical chi square  609.9  with prob <  0.99
The total n.obs was  560  with Likelihood Chi Square =  2340.66  with prob <  7.2e-177

Tucker Lewis Index of factoring reliability =  0.884
RMSEA index =  0.065  and the 90 % confidence intervals are  0.062 0.068
BIC =  -2057.25
Fit based upon off diagonal values = 1
Measures of factor score adequacy
                                                 ML2  ML5  ML1  ML6  ML8  ML3
Correlation of (regression) scores with factors   0.98 0.96 0.97 0.97 0.95 0.98
```

```
Multiple R square of scores with factors          0.96 0.93 0.94 0.95 0.89 0.95
Minimum correlation of possible factor scores     0.92 0.85 0.87 0.89 0.79 0.91
                                                   ML7  ML4
Correlation of (regression) scores with factors   0.95 0.95
Multiple R square of scores with factors          0.90 0.90
Minimum correlation of possible factor scores     0.80 0.80
```

```
#stepwise deletion of items with loading below .55
final8withoutUSEF5<- c("SOCI2", "SOCI3", "USEF1", "USEF2", "USEF5")
final8_USEF5 <- nor[ ,!(names(nor) %in% final8withoutUSEF5)]
final8f1 = fa(final8_USEF5, nfactors=8, rotate="oblimin", fm="ml")
final8f1
```

```
Factor Analysis using method =  ml
Call: fa(r = final8_USEF5, nfactors = 8, rotate = "oblimin", fm = "ml")
Standardized loadings (pattern matrix) based upon correlation matrix
        ML2   ML1   ML5   ML6   ML8   ML3   ML7   ML4   h2    u2 com
COMP1 -0.01  0.04  0.77  0.05  0.01  0.05 -0.06  0.04 0.69 0.310 1.0
COMP2 -0.01  0.03  0.80  0.06 -0.04  0.01 -0.03  0.09 0.73 0.271 1.0
COMP3  0.01  0.04  0.78  0.02  0.05  0.07 -0.04  0.02 0.73 0.268 1.0
COMP4  0.08  0.01  0.72 -0.01  0.13 -0.06  0.15 -0.03 0.72 0.285 1.2
COMP5  0.06 -0.04  0.81  0.03  0.00 -0.01  0.10 -0.02 0.73 0.271 1.0
CONF1  0.72  0.07  0.13  0.01  0.03  0.04 -0.03  0.03 0.67 0.327 1.1
CONF2  0.94  0.03  0.00 -0.01 -0.02 -0.02 -0.03  0.01 0.85 0.153 1.0
CONF3  0.93 -0.02 -0.01 -0.02 -0.02 -0.03  0.05 -0.01 0.85 0.153 1.0
CONF4  0.93  0.02  0.01 -0.02 -0.01  0.02  0.00  0.00 0.86 0.136 1.0
CONF5  0.89 -0.05 -0.02  0.04  0.05  0.02  0.00  0.02 0.85 0.151 1.0
INSE1  0.11  0.09  0.15  0.11  0.04  0.53 -0.03 -0.01 0.52 0.477 1.4
INSE2  0.01  0.00  0.02 -0.03  0.01  0.94  0.03 -0.02 0.90 0.097 1.0
INSE3  0.02 -0.06  0.05  0.07  0.01  0.86 -0.01  0.00 0.78 0.224 1.0
INSE4 -0.04  0.02 -0.07 -0.03 -0.02  0.88  0.01  0.03 0.76 0.245 1.0
INSE5 -0.01  0.02 -0.02 -0.04  0.01  0.88  0.01  0.04 0.79 0.210 1.0
PRIV1  0.09 -0.03  0.09  0.11  0.02  0.12  0.67 -0.11 0.62 0.378 1.3
PRIV2  0.05  0.00  0.04  0.08  0.02  0.07  0.78 -0.10 0.69 0.313 1.1
PRIV3 -0.01  0.03  0.01 -0.03 -0.08  0.00  0.72  0.25 0.72 0.283 1.3
PRIV4 -0.01  0.11 -0.01 -0.03  0.02  0.02  0.77  0.05 0.68 0.316 1.1
PRIV5 -0.02  0.00  0.02  0.01  0.05 -0.04  0.67  0.18 0.61 0.392 1.2
OVER1  0.01  0.09  0.19  0.06  0.67 -0.02 -0.08  0.02 0.71 0.291 1.3
OVER2 -0.08  0.11  0.19  0.05  0.70  0.06 -0.04  0.01 0.75 0.250 1.3
OVER3  0.09  0.04 -0.13 -0.03  0.67  0.04  0.12  0.01 0.55 0.448 1.2
OVER4  0.07 -0.05 -0.01  0.08  0.69  0.08  0.01  0.02 0.60 0.402 1.1
OVER5  0.12  0.05  0.19 -0.05  0.51  0.00  0.06  0.11 0.57 0.434 1.6
```

```
SAFE1  0.03 -0.08 -0.01  0.12  0.13  0.04  0.11  0.63 0.62 0.383 1.3
SAFE2 -0.01 -0.03 -0.04  0.07  0.14  0.06  0.06  0.66 0.60 0.397 1.2
SAFE3  0.02  0.02 -0.03  0.02  0.09  0.00  0.14  0.58 0.50 0.500 1.2
SAFE4 -0.01  0.07  0.04 -0.03 -0.03  0.03  0.03  0.85 0.79 0.211 1.0
SAFE5  0.07  0.02  0.10  0.03 -0.09  0.05 -0.03  0.77 0.68 0.325 1.1
SOCI1  0.11  0.12 -0.09  0.07  0.48  0.13  0.02  0.02 0.46 0.540 1.5
SOCI4  0.21  0.04 -0.08  0.08  0.36 -0.02  0.23  0.15 0.51 0.490 3.2
SOCI5  0.26  0.07 -0.14  0.17  0.33  0.05  0.10  0.11 0.51 0.489 3.6
TECH1  0.14  0.10 -0.01  0.53  0.12  0.05  0.00  0.10 0.62 0.384 1.4
TECH2  0.05  0.01  0.02  0.76  0.06  0.00  0.02  0.03 0.70 0.298 1.0
TECH3 -0.02 -0.04  0.02  0.96 -0.02 -0.04  0.03 -0.01 0.86 0.137 1.0
TECH4 -0.02  0.08 -0.01  0.90 -0.03  0.03 -0.01 -0.04 0.85 0.152 1.0
TECH5  0.00  0.07  0.04  0.67  0.00  0.01 -0.03  0.12 0.61 0.388 1.1
USEF3 -0.01  0.12  0.33  0.20  0.14 -0.03  0.13 -0.01 0.45 0.547 2.8
USEF4  0.02  0.22  0.19  0.00  0.34 -0.01  0.12 -0.04 0.42 0.580 2.7
UNRE1  0.00  0.72  0.06  0.06  0.02 -0.01  0.11 -0.04 0.68 0.318 1.1
UNRE2 -0.02  0.88  0.05 -0.01 -0.05  0.05  0.04 -0.04 0.78 0.218 1.0
UNRE3  0.08  0.84 -0.10  0.05  0.04 -0.01 -0.03 -0.01 0.75 0.251 1.1
UNRE4  0.00  0.85  0.01  0.05  0.00  0.00 -0.02  0.07 0.82 0.185 1.0
UNRE5 -0.06  0.66  0.07  0.01  0.15 -0.04  0.00  0.07 0.61 0.393 1.2

                       ML2  ML1  ML5  ML6  ML8  ML3  ML7  ML4
SS loadings           4.60 4.01 3.97 3.90 3.82 3.80 3.39 3.24
Proportion Var        0.10 0.09 0.09 0.09 0.08 0.08 0.08 0.07
Cumulative Var        0.10 0.19 0.28 0.37 0.45 0.54 0.61 0.68
Proportion Explained  0.15 0.13 0.13 0.13 0.12 0.12 0.11 0.11
Cumulative Proportion 0.15 0.28 0.41 0.54 0.66 0.78 0.89 1.00

 With factor correlations of
     ML2  ML1  ML5  ML6  ML8  ML3  ML7  ML4
ML2 1.00 0.22 0.29 0.34 0.48 0.22 0.37 0.27
ML1 0.22 1.00 0.44 0.68 0.50 0.25 0.28 0.29
ML5 0.29 0.44 1.00 0.51 0.43 0.23 0.25 0.26
ML6 0.34 0.68 0.51 1.00 0.46 0.23 0.31 0.31
ML8 0.48 0.50 0.43 0.46 1.00 0.32 0.30 0.29
ML3 0.22 0.25 0.23 0.23 0.32 1.00 0.34 0.37
ML7 0.37 0.28 0.25 0.31 0.30 0.34 1.00 0.50
ML4 0.27 0.29 0.26 0.31 0.29 0.37 0.50 1.00

Mean item complexity =  1.3
Test of the hypothesis that 8 factors are sufficient.

df null model =  990  with the objective function =  40.53 with Chi Square =  22014.77
```

```
df of  the model are 658  and the objective function was  4.16

The root mean square of the residuals (RMSR) is  0.02
The df corrected root mean square of the residuals is  0.03

The harmonic n.obs is  560 with the empirical chi square  559.77  with prob <  1
The total n.obs was  560  with Likelihood Chi Square =  2239.71  with prob <  3.3e-171

Tucker Lewis Index of factoring reliability =  0.886
RMSEA index =  0.065  and the 90 % confidence intervals are  0.063 0.069
BIC =  -1924.07
Fit based upon off diagonal values = 1
Measures of factor score adequacy
                                                   ML2  ML1  ML5  ML6  ML8  ML3
Correlation of (regression) scores with factors   0.98 0.97 0.96 0.97 0.95 0.98
Multiple R square of scores with factors          0.96 0.94 0.92 0.95 0.89 0.95
Minimum correlation of possible factor scores     0.92 0.87 0.85 0.89 0.79 0.91
                                                   ML7  ML4
Correlation of (regression) scores with factors   0.95 0.95
Multiple R square of scores with factors          0.90 0.90
Minimum correlation of possible factor scores     0.80 0.80
```

```
final8withoutUSEF4<- c("SOCI2", "SOCI3", "USEF1", "USEF2", "USEF5", "USEF4")
final8_USEF4 <- nor[ ,!(names(nor) %in% final8withoutUSEF4)]
final8f2 = fa(final8_USEF4, nfactors=8, rotate="oblimin", fm="ml")
final8f2
```

```
Factor Analysis using method =  ml
Call: fa(r = final8_USEF4, nfactors = 8, rotate = "oblimin", fm = "ml")
Standardized loadings (pattern matrix) based upon correlation matrix
        ML2   ML8   ML1   ML5   ML3   ML6   ML7   ML4   h2    u2 com
COMP1 -0.01  0.05  0.05  0.76  0.05  0.02 -0.06  0.04 0.69 0.312 1.0
COMP2 -0.01  0.03  0.06  0.79  0.01 -0.02 -0.03  0.09 0.73 0.274 1.0
COMP3  0.01  0.05  0.02  0.78  0.07  0.05 -0.04  0.02 0.73 0.271 1.0
COMP4  0.08  0.02 -0.02  0.72 -0.06  0.13  0.15 -0.03 0.72 0.280 1.2
COMP5  0.06 -0.03  0.02  0.81 -0.01  0.00  0.10 -0.02 0.73 0.266 1.0
CONF1  0.72  0.07  0.01  0.13  0.04  0.04 -0.02  0.03 0.67 0.327 1.1
CONF2  0.94  0.03 -0.01  0.01 -0.02 -0.02 -0.03  0.01 0.85 0.153 1.0
CONF3  0.93 -0.01 -0.02  0.00 -0.03 -0.03  0.05 -0.01 0.85 0.153 1.0
CONF4  0.93  0.01 -0.02  0.01  0.02 -0.01  0.00  0.00 0.86 0.137 1.0
CONF5  0.89 -0.05  0.04 -0.02  0.02  0.05  0.00  0.02 0.85 0.151 1.0
```

INSE1	0.11	0.08	0.11	0.14	0.53	0.06	-0.02	-0.02	0.52	0.477	1.4
INSE2	0.01	0.00	-0.02	0.02	0.94	0.01	0.03	-0.02	0.90	0.097	1.0
INSE3	0.02	-0.06	0.07	0.04	0.86	0.02	-0.01	0.00	0.78	0.225	1.0
INSE4	-0.04	0.03	-0.03	-0.06	0.88	-0.03	0.01	0.03	0.76	0.244	1.0
INSE5	-0.01	0.02	-0.04	-0.02	0.88	0.00	0.01	0.04	0.79	0.210	1.0
PRIV1	0.09	-0.03	0.11	0.09	0.11	0.03	0.70	-0.11	0.64	0.359	1.2
PRIV2	0.05	0.01	0.07	0.04	0.06	0.02	0.80	-0.10	0.71	0.294	1.1
PRIV3	-0.02	0.03	-0.03	0.02	-0.01	-0.08	0.71	0.27	0.71	0.294	1.3
PRIV4	-0.01	0.11	-0.04	-0.01	0.02	0.02	0.75	0.06	0.67	0.327	1.1
PRIV5	-0.03	0.01	0.00	0.01	-0.04	0.06	0.65	0.20	0.60	0.403	1.2
OVER1	-0.01	0.07	0.05	0.16	-0.03	0.74	-0.07	0.01	0.75	0.246	1.1
OVER2	-0.09	0.08	0.04	0.16	0.05	0.76	-0.02	0.01	0.79	0.210	1.2
OVER3	0.11	0.04	-0.04	-0.14	0.04	0.64	0.13	0.01	0.53	0.475	1.3
OVER4	0.09	-0.04	0.07	-0.03	0.08	0.66	0.02	0.02	0.57	0.431	1.1
OVER5	0.14	0.06	-0.05	0.18	0.00	0.47	0.06	0.11	0.54	0.457	1.7
SAFE1	0.04	-0.07	0.12	-0.01	0.05	0.11	0.10	0.64	0.62	0.380	1.2
SAFE2	0.00	-0.03	0.07	-0.04	0.06	0.13	0.06	0.67	0.61	0.395	1.1
SAFE3	0.02	0.03	0.01	-0.03	0.00	0.08	0.14	0.58	0.50	0.498	1.2
SAFE4	-0.01	0.06	-0.03	0.04	0.03	-0.03	0.02	0.85	0.78	0.219	1.0
SAFE5	0.07	0.01	0.03	0.10	0.05	-0.08	-0.04	0.77	0.67	0.334	1.1
SOCI1	0.12	0.12	0.07	-0.11	0.12	0.48	0.03	0.02	0.46	0.545	1.6
SOCI4	0.22	0.05	0.08	-0.09	-0.02	0.32	0.23	0.15	0.50	0.502	3.6
SOCI5	0.27	0.08	0.17	-0.14	0.05	0.31	0.11	0.11	0.50	0.497	3.9
TECH1	0.14	0.10	0.53	-0.01	0.05	0.10	0.00	0.10	0.62	0.385	1.4
TECH2	0.05	0.01	0.76	0.02	0.00	0.05	0.01	0.04	0.70	0.297	1.0
TECH3	-0.02	-0.04	0.95	0.02	-0.04	-0.01	0.03	-0.01	0.86	0.138	1.0
TECH4	-0.03	0.08	0.90	-0.01	0.03	-0.03	-0.01	-0.04	0.85	0.152	1.0
TECH5	0.00	0.06	0.67	0.04	0.01	0.01	-0.03	0.12	0.61	0.389	1.1
USEF3	0.01	0.13	0.20	0.33	-0.03	0.11	0.12	0.00	0.44	0.556	2.7
UNRE1	0.00	0.72	0.06	0.06	-0.01	0.01	0.10	-0.04	0.68	0.320	1.1
UNRE2	-0.03	0.88	-0.01	0.06	0.05	-0.04	0.04	-0.05	0.78	0.218	1.0
UNRE3	0.08	0.85	0.05	-0.10	-0.01	0.03	-0.03	-0.01	0.75	0.250	1.1
UNRE4	0.00	0.85	0.04	0.01	0.00	0.00	-0.03	0.07	0.82	0.183	1.0
UNRE5	-0.07	0.65	0.01	0.07	-0.04	0.16	0.00	0.07	0.61	0.392	1.2

	ML2	ML8	ML1	ML5	ML3	ML6	ML7	ML4
SS loadings	4.63	3.90	3.86	3.80	3.79	3.66	3.35	3.30
Proportion Var	0.11	0.09	0.09	0.09	0.09	0.08	0.08	0.07
Cumulative Var	0.11	0.19	0.28	0.37	0.45	0.54	0.61	0.69
Proportion Explained	0.15	0.13	0.13	0.13	0.13	0.12	0.11	0.11
Cumulative Proportion	0.15	0.28	0.41	0.53	0.66	0.78	0.89	1.00

With factor correlations of

```
     ML2  ML8  ML1  ML5  ML3  ML6  ML7  ML4
ML2 1.00 0.22 0.34 0.28 0.22 0.48 0.37 0.27
ML8 0.22 1.00 0.68 0.43 0.25 0.52 0.29 0.30
ML1 0.34 0.68 1.00 0.51 0.23 0.48 0.31 0.31
ML5 0.28 0.43 0.51 1.00 0.22 0.45 0.24 0.26
ML3 0.22 0.25 0.23 0.22 1.00 0.32 0.34 0.37
ML6 0.48 0.52 0.48 0.45 0.32 1.00 0.30 0.29
ML7 0.37 0.29 0.31 0.24 0.34 0.30 1.00 0.49
ML4 0.27 0.30 0.31 0.26 0.37 0.29 0.49 1.00

Mean item complexity =  1.3
Test of the hypothesis that 8 factors are sufficient.

df null model =  946  with the objective function =  39.78 with Chi Square =  21620.65
df of  the model are 622  and the objective function was  3.92

The root mean square of the residuals (RMSR) is  0.02
The df corrected root mean square of the residuals is  0.03

The harmonic n.obs is  560 with the empirical chi square  511.5  with prob <  1
The total n.obs was  560  with Likelihood Chi Square =  2111.02  with prob <  4.9e-161

Tucker Lewis Index of factoring reliability =  0.889
RMSEA index =  0.065  and the 90 % confidence intervals are  0.062 0.069
BIC =  -1824.96
Fit based upon off diagonal values = 1
Measures of factor score adequacy
                                                   ML2  ML8  ML1  ML5  ML3  ML6
Correlation of (regression) scores with factors   0.98 0.97 0.97 0.96 0.98 0.95
Multiple R square of scores with factors          0.96 0.94 0.95 0.92 0.95 0.90
Minimum correlation of possible factor scores     0.92 0.87 0.89 0.85 0.91 0.81
                                                   ML7  ML4
Correlation of (regression) scores with factors   0.95 0.95
Multiple R square of scores with factors          0.90 0.90
Minimum correlation of possible factor scores     0.80 0.80
```

```r
final8withoutUSEF3<- c("SOCI2", "SOCI3", "USEF1", "USEF2", "USEF5", "USEF4", "USEF3")
final8_USEF3 <- nor[ ,!(names(nor) %in% final8withoutUSEF3)]
final8f3 = fa(final8_USEF3, nfactors=8, rotate="oblimin", fm="ml")
final8f3
```

```
Factor Analysis using method =  ml
```

Call: fa(r = final8_USEF3, nfactors = 8, rotate = "oblimin", fm = "ml")

Standardized loadings (pattern matrix) based upon correlation matrix

	ML2	ML8	ML3	ML1	ML6	ML5	ML4	ML7	h2	u2	com
COMP1	-0.01	0.05	0.05	0.05	0.03	0.76	0.04	-0.06	0.69	0.312	1.0
COMP2	-0.01	0.03	0.01	0.06	-0.02	0.79	0.08	-0.02	0.73	0.275	1.0
COMP3	0.02	0.05	0.07	0.02	0.05	0.77	0.02	-0.04	0.73	0.274	1.0
COMP4	0.08	0.02	-0.06	-0.02	0.13	0.72	-0.03	0.15	0.72	0.279	1.2
COMP5	0.06	-0.02	-0.01	0.02	0.00	0.81	-0.01	0.10	0.73	0.267	1.0
CONF1	0.72	0.07	0.04	0.01	0.04	0.13	0.02	-0.02	0.67	0.326	1.1
CONF2	0.94	0.03	-0.02	0.00	-0.02	0.01	0.00	-0.03	0.85	0.153	1.0
CONF3	0.93	-0.01	-0.03	-0.02	-0.03	0.00	-0.01	0.05	0.85	0.152	1.0
CONF4	0.93	0.01	0.02	-0.01	0.00	0.01	0.00	0.00	0.86	0.138	1.0
CONF5	0.89	-0.05	0.02	0.04	0.05	-0.02	0.02	0.00	0.85	0.152	1.0
INSE1	0.10	0.08	0.53	0.11	0.07	0.14	-0.02	-0.02	0.52	0.477	1.4
INSE2	0.01	0.00	0.94	-0.02	0.01	0.02	-0.02	0.03	0.90	0.098	1.0
INSE3	0.02	-0.06	0.86	0.07	0.02	0.04	0.00	0.00	0.78	0.225	1.0
INSE4	-0.04	0.03	0.88	-0.03	-0.03	-0.06	0.03	0.00	0.76	0.243	1.0
INSE5	-0.01	0.02	0.88	-0.04	0.00	-0.02	0.05	0.01	0.79	0.209	1.0
PRIV1	0.07	-0.03	0.11	0.10	0.03	0.09	-0.11	0.72	0.67	0.329	1.2
PRIV2	0.04	0.01	0.05	0.07	0.02	0.04	-0.09	0.82	0.74	0.263	1.1
PRIV3	-0.02	0.04	-0.01	-0.03	-0.08	0.02	0.29	0.68	0.69	0.309	1.4
PRIV4	-0.01	0.12	0.02	-0.04	0.02	-0.01	0.10	0.72	0.65	0.347	1.1
PRIV5	-0.02	0.01	-0.04	0.00	0.06	0.01	0.23	0.61	0.58	0.422	1.3
OVER1	-0.01	0.06	-0.03	0.05	0.76	0.16	0.00	-0.06	0.76	0.239	1.1
OVER2	-0.09	0.07	0.04	0.04	0.77	0.15	0.00	-0.02	0.80	0.204	1.1
OVER3	0.12	0.04	0.04	-0.04	0.64	-0.15	0.02	0.13	0.52	0.479	1.3
OVER4	0.09	-0.04	0.08	0.07	0.65	-0.03	0.02	0.03	0.56	0.435	1.1
OVER5	0.15	0.06	0.00	-0.05	0.47	0.18	0.12	0.06	0.54	0.461	1.8
SAFE1	0.04	-0.07	0.05	0.12	0.10	-0.01	0.64	0.10	0.62	0.377	1.2
SAFE2	0.00	-0.03	0.06	0.07	0.12	-0.04	0.67	0.05	0.61	0.392	1.1
SAFE3	0.03	0.03	0.00	0.01	0.07	-0.03	0.59	0.12	0.50	0.496	1.1
SAFE4	-0.01	0.06	0.03	-0.03	-0.03	0.04	0.84	0.02	0.77	0.227	1.0
SAFE5	0.07	0.01	0.05	0.04	-0.07	0.11	0.76	-0.04	0.66	0.340	1.1
SOCI1	0.12	0.12	0.12	0.07	0.48	-0.11	0.02	0.03	0.45	0.546	1.6
SOCI4	0.22	0.05	-0.02	0.08	0.32	-0.09	0.16	0.23	0.50	0.503	3.8
SOCI5	0.28	0.08	0.05	0.17	0.30	-0.14	0.11	0.11	0.50	0.498	4.0
TECH1	0.14	0.10	0.05	0.53	0.10	-0.01	0.10	0.00	0.61	0.386	1.4
TECH2	0.05	0.01	0.00	0.76	0.05	0.02	0.04	0.01	0.70	0.299	1.0
TECH3	-0.02	-0.04	-0.04	0.95	-0.01	0.02	-0.01	0.03	0.86	0.139	1.0
TECH4	-0.03	0.08	0.03	0.90	-0.03	-0.01	-0.05	0.00	0.85	0.151	1.0
TECH5	0.00	0.06	0.01	0.67	0.01	0.04	0.12	-0.03	0.61	0.388	1.1
UNRE1	0.00	0.72	-0.01	0.06	0.01	0.06	-0.03	0.10	0.68	0.323	1.1
UNRE2	-0.03	0.88	0.05	-0.01	-0.04	0.06	-0.05	0.04	0.78	0.217	1.0

```
UNRE3  0.08  0.85 -0.01  0.05  0.03 -0.10 -0.02 -0.02 0.75 0.250 1.1
UNRE4  0.00  0.85  0.00  0.04  0.00  0.01  0.07 -0.03 0.82 0.183 1.0
UNRE5 -0.06  0.65 -0.04  0.01  0.16  0.07  0.07 -0.01 0.61 0.392 1.2

                       ML2  ML8  ML3  ML1  ML6  ML5  ML4  ML7
SS loadings           4.63 3.85 3.79 3.75 3.60 3.60 3.35 3.26
Proportion Var        0.11 0.09 0.09 0.09 0.08 0.08 0.08 0.08
Cumulative Var        0.11 0.20 0.29 0.37 0.46 0.54 0.62 0.69
Proportion Explained  0.16 0.13 0.13 0.13 0.12 0.12 0.11 0.11
Cumulative Proportion 0.16 0.28 0.41 0.54 0.66 0.78 0.89 1.00

 With factor correlations of
     ML2  ML8  ML3  ML1  ML6  ML5  ML4  ML7
ML2 1.00 0.23 0.22 0.34 0.47 0.28 0.27 0.38
ML8 0.23 1.00 0.25 0.68 0.52 0.42 0.30 0.28
ML3 0.22 0.25 1.00 0.23 0.32 0.22 0.37 0.34
ML1 0.34 0.68 0.23 1.00 0.48 0.50 0.30 0.31
ML6 0.47 0.52 0.32 0.48 1.00 0.45 0.29 0.30
ML5 0.28 0.42 0.22 0.50 0.45 1.00 0.26 0.24
ML4 0.27 0.30 0.37 0.30 0.29 0.26 1.00 0.48
ML7 0.38 0.28 0.34 0.31 0.30 0.24 0.48 1.00

Mean item complexity =  1.3
Test of the hypothesis that 8 factors are sufficient.

df null model =  903  with the objective function =  39.11 with Chi Square =  21267.69
df of  the model are 587  and the objective function was  3.81

The root mean square of the residuals (RMSR) is  0.02
The df corrected root mean square of the residuals is  0.03

The harmonic n.obs is  560 with the empirical chi square  493.12  with prob <  1
The total n.obs was  560  with Likelihood Chi Square =  2052.69  with prob <  1.9e-161

Tucker Lewis Index of factoring reliability =  0.888
RMSEA index =  0.067  and the 90 % confidence intervals are  0.064 0.07
BIC =  -1661.8
Fit based upon off diagonal values = 1
Measures of factor score adequacy
                                                   ML2  ML8  ML3  ML1  ML6  ML5
Correlation of (regression) scores with factors   0.98 0.97 0.98 0.97 0.95 0.96
Multiple R square of scores with factors          0.96 0.94 0.95 0.94 0.90 0.92
Minimum correlation of possible factor scores     0.92 0.87 0.91 0.89 0.81 0.84
```

```
                                                 ML4  ML7
Correlation of (regression) scores with factors   0.95 0.95
Multiple R square of scores with factors          0.90 0.90
Minimum correlation of possible factor scores     0.80 0.80
```

```
final8withoutSOCI5<- c("SOCI2", "SOCI3", "USEF1", "USEF2", "USEF5", "USEF4", "USEF3", "SOC
final8_SOCI5 <- nor[ ,!(names(nor) %in% final8withoutSOCI5)]
final8f4 = fa(final8_SOCI5, nfactors=8, rotate="oblimin", fm="ml")
final8f4
```

```
Factor Analysis using method =  ml
Call: fa(r = final8_SOCI5, nfactors = 8, rotate = "oblimin", fm = "ml")
Standardized loadings (pattern matrix) based upon correlation matrix
        ML2   ML8   ML3   ML1   ML5   ML6   ML4   ML7   h2    u2 com
COMP1 -0.01  0.04  0.05  0.05  0.76  0.03  0.04 -0.06 0.69 0.315 1.0
COMP2 -0.02  0.03  0.01  0.06  0.79 -0.01  0.08 -0.03 0.72 0.280 1.0
COMP3  0.01  0.05  0.07  0.02  0.78  0.05  0.02 -0.04 0.73 0.273 1.0
COMP4  0.08  0.03 -0.06 -0.02  0.73  0.12 -0.03  0.15 0.73 0.274 1.2
COMP5  0.06 -0.02 -0.01  0.02  0.83 -0.02 -0.02  0.10 0.74 0.260 1.0
CONF1  0.72  0.07  0.04  0.01  0.13  0.04  0.02 -0.02 0.67 0.326 1.1
CONF2  0.94  0.03 -0.02  0.00  0.01 -0.02  0.01 -0.03 0.85 0.153 1.0
CONF3  0.93 -0.01 -0.03 -0.01  0.00 -0.03 -0.01  0.05 0.85 0.151 1.0
CONF4  0.92  0.01  0.02 -0.01  0.01  0.00  0.00  0.00 0.86 0.138 1.0
CONF5  0.89 -0.05  0.02  0.04 -0.02  0.04  0.02  0.00 0.85 0.153 1.0
INSE1  0.10  0.07  0.52  0.12  0.13  0.09 -0.02 -0.02 0.52 0.476 1.4
INSE2  0.00  0.00  0.94 -0.02  0.02  0.01 -0.02  0.03 0.90 0.098 1.0
INSE3  0.01 -0.06  0.86  0.07  0.04  0.03  0.00 -0.01 0.78 0.225 1.0
INSE4 -0.03  0.03  0.88 -0.03 -0.06 -0.04  0.03  0.00 0.76 0.242 1.0
INSE5 -0.01  0.02  0.88 -0.04 -0.02  0.00  0.05  0.01 0.79 0.209 1.0
PRIV1  0.08 -0.03  0.11  0.10  0.09  0.03 -0.11  0.72 0.66 0.336 1.2
PRIV2  0.04  0.01  0.05  0.07  0.04  0.02 -0.09  0.81 0.73 0.271 1.1
PRIV3 -0.02  0.03 -0.01 -0.03  0.01 -0.07  0.29  0.68 0.69 0.305 1.4
PRIV4 -0.01  0.12  0.02 -0.03 -0.02  0.02  0.09  0.73 0.66 0.341 1.1
PRIV5 -0.02  0.01 -0.04  0.00  0.01  0.06  0.23  0.62 0.58 0.416 1.3
OVER1 -0.01  0.04 -0.04  0.05  0.11  0.80  0.00 -0.05 0.79 0.209 1.1
OVER2 -0.08  0.05  0.04  0.04  0.11  0.82  0.00 -0.01 0.82 0.175 1.1
OVER3  0.14  0.05  0.04 -0.04 -0.16  0.61  0.02  0.13 0.50 0.503 1.4
OVER4  0.12 -0.03  0.08  0.06 -0.04  0.62  0.03  0.03 0.54 0.463 1.1
OVER5  0.17  0.07  0.01 -0.06  0.18  0.43  0.12  0.06 0.52 0.477 2.0
SAFE1  0.05 -0.06  0.05  0.12 -0.01  0.09  0.64  0.10 0.62 0.380 1.2
SAFE2  0.01 -0.02  0.06  0.07 -0.04  0.11  0.67  0.06 0.61 0.394 1.1
```

SAFE3	0.03	0.03	0.00	0.01	-0.03	0.07	0.59	0.13	0.50	0.496	1.1
SAFE4	-0.01	0.06	0.03	-0.03	0.04	-0.02	0.84	0.02	0.78	0.225	1.0
SAFE5	0.06	0.01	0.05	0.04	0.11	-0.07	0.76	-0.04	0.66	0.341	1.1
SOCI1	0.13	0.12	0.13	0.06	-0.12	0.47	0.02	0.04	0.44	0.555	1.7
SOCI4	0.24	0.07	-0.01	0.07	-0.08	0.28	0.16	0.23	0.48	0.523	4.1
TECH1	0.15	0.11	0.05	0.52	-0.01	0.09	0.11	0.00	0.61	0.388	1.4
TECH2	0.06	0.01	0.00	0.75	0.02	0.06	0.04	0.01	0.70	0.299	1.0
TECH3	-0.02	-0.04	-0.04	0.95	0.02	-0.01	-0.01	0.03	0.86	0.137	1.0
TECH4	-0.02	0.08	0.03	0.90	-0.01	-0.02	-0.05	0.00	0.85	0.151	1.0
TECH5	0.00	0.06	0.01	0.67	0.04	0.01	0.12	-0.03	0.61	0.389	1.1
UNRE1	0.00	0.72	-0.01	0.06	0.06	0.02	-0.03	0.10	0.68	0.323	1.1
UNRE2	-0.04	0.88	0.05	-0.01	0.07	-0.03	-0.05	0.04	0.78	0.219	1.0
UNRE3	0.09	0.85	0.00	0.05	-0.09	0.02	-0.02	-0.02	0.75	0.250	1.1
UNRE4	0.00	0.85	0.00	0.04	0.02	0.00	0.07	-0.03	0.82	0.181	1.0
UNRE5	-0.06	0.65	-0.04	0.01	0.06	0.17	0.07	-0.01	0.61	0.391	1.2

	ML2	ML8	ML3	ML1	ML5	ML6	ML4	ML7
SS loadings	4.53	3.79	3.77	3.64	3.62	3.41	3.30	3.23
Proportion Var	0.11	0.09	0.09	0.09	0.09	0.08	0.08	0.08
Cumulative Var	0.11	0.20	0.29	0.37	0.46	0.54	0.62	0.70
Proportion Explained	0.15	0.13	0.13	0.12	0.12	0.12	0.11	0.11
Cumulative Proportion	0.15	0.28	0.41	0.54	0.66	0.78	0.89	1.00

With factor correlations of

	ML2	ML8	ML3	ML1	ML5	ML6	ML4	ML7
ML2	1.00	0.22	0.22	0.33	0.29	0.46	0.27	0.37
ML8	0.22	1.00	0.25	0.68	0.43	0.54	0.30	0.28
ML3	0.22	0.25	1.00	0.22	0.23	0.32	0.37	0.34
ML1	0.33	0.68	0.22	1.00	0.51	0.49	0.30	0.30
ML5	0.29	0.43	0.23	0.51	1.00	0.49	0.27	0.25
ML6	0.46	0.54	0.32	0.49	0.49	1.00	0.29	0.29
ML4	0.27	0.30	0.37	0.30	0.27	0.29	1.00	0.49
ML7	0.37	0.28	0.34	0.30	0.25	0.29	0.49	1.00

```
Mean item complexity =  1.2
Test of the hypothesis that 8 factors are sufficient.

df null model =  861  with the objective function =  38.23 with Chi Square =  20805.39
df of  the model are 553  and the objective function was  3.59

The root mean square of the residuals (RMSR) is  0.02
The df corrected root mean square of the residuals is  0.03
```

```
The harmonic n.obs is  560 with the empirical chi square  463.94  with prob <  1
The total n.obs was  560  with Likelihood Chi Square =  1936.22  with prob <  1.3e-152

Tucker Lewis Index of factoring reliability =  0.891
RMSEA index =  0.067  and the 90 % confidence intervals are  0.064 0.07
BIC =  -1563.13
Fit based upon off diagonal values = 1
Measures of factor score adequacy
                                                   ML2  ML8  ML3  ML1  ML5  ML6
Correlation of (regression) scores with factors   0.98 0.97 0.98 0.97 0.96 0.96
Multiple R square of scores with factors          0.96 0.94 0.95 0.94 0.92 0.91
Minimum correlation of possible factor scores     0.92 0.87 0.91 0.89 0.84 0.83
                                                   ML4  ML7
Correlation of (regression) scores with factors   0.95 0.95
Multiple R square of scores with factors          0.90 0.90
Minimum correlation of possible factor scores     0.80 0.80
```

```
final8withoutSOCI4<- c("SOCI2", "SOCI3", "USEF1", "USEF2", "USEF5", "USEF4", "USEF3", "SOC
final8_SOCI4 <- nor[ ,!(names(nor) %in% final8withoutSOCI4)]
final8f5 = fa(final8_SOCI4, nfactors=8, rotate="oblimin", fm="ml")
final8f5
```

```
Factor Analysis using method =  ml
Call: fa(r = final8_SOCI4, nfactors = 8, rotate = "oblimin", fm = "ml")
Standardized loadings (pattern matrix) based upon correlation matrix
        ML2   ML3   ML8   ML5   ML1   ML6   ML4   ML7   h2    u2 com
COMP1 -0.02  0.05  0.04  0.77  0.04  0.03  0.04 -0.07 0.69 0.314 1.0
COMP2 -0.02  0.01  0.03  0.80  0.06 -0.01  0.08 -0.03 0.72 0.281 1.0
COMP3  0.01  0.07  0.05  0.79  0.01  0.05  0.02 -0.05 0.73 0.271 1.0
COMP4  0.08 -0.06  0.02  0.73 -0.03  0.12 -0.04  0.15 0.73 0.274 1.2
COMP5  0.05 -0.01 -0.02  0.83  0.01 -0.02 -0.02  0.09 0.74 0.260 1.0
CONF1  0.71  0.04  0.07  0.12  0.01  0.04  0.03 -0.02 0.67 0.325 1.1
CONF2  0.94 -0.02  0.03  0.00  0.00 -0.02  0.01 -0.03 0.85 0.152 1.0
CONF3  0.93 -0.03 -0.01  0.00 -0.01 -0.03 -0.01  0.05 0.85 0.150 1.0
CONF4  0.92  0.02  0.01  0.01 -0.01  0.00  0.00  0.00 0.86 0.138 1.0
CONF5  0.89  0.02 -0.05 -0.02  0.04  0.04  0.02  0.00 0.85 0.155 1.0
INSE1  0.10  0.52  0.07  0.13  0.12  0.10 -0.02 -0.02 0.52 0.476 1.4
INSE2  0.00  0.94  0.00  0.02 -0.02  0.01 -0.02  0.03 0.90 0.098 1.0
INSE3  0.01  0.86 -0.07  0.04  0.07  0.03  0.00 -0.01 0.77 0.225 1.0
INSE4 -0.03  0.88  0.03 -0.05 -0.03 -0.04  0.03  0.00 0.76 0.242 1.0
INSE5 -0.01  0.88  0.02 -0.02 -0.04  0.00  0.05  0.01 0.79 0.209 1.0
```

```
PRIV1  0.09  0.11 -0.03  0.09  0.11  0.03 -0.11  0.69 0.64 0.365 1.2
PRIV2  0.06  0.06  0.01  0.04  0.07  0.01 -0.10  0.79 0.70 0.302 1.1
PRIV3 -0.02 -0.01  0.02  0.01 -0.02 -0.06  0.26  0.71 0.71 0.289 1.3
PRIV4 -0.01  0.02  0.11 -0.02 -0.03  0.03  0.06  0.76 0.68 0.320 1.1
PRIV5 -0.02 -0.04  0.00  0.00  0.01  0.07  0.20  0.66 0.60 0.398 1.2
OVER1  0.00 -0.04  0.03  0.09  0.05  0.83  0.01 -0.04 0.81 0.193 1.0
OVER2 -0.08  0.03  0.04  0.10  0.03  0.84  0.01  0.00 0.84 0.164 1.1
OVER3  0.16  0.05  0.06 -0.16 -0.05  0.59  0.02  0.13 0.48 0.520 1.5
OVER4  0.13  0.08 -0.02 -0.04  0.05  0.60  0.03  0.03 0.52 0.478 1.2
OVER5  0.18  0.01  0.08  0.18 -0.06  0.41  0.12  0.06 0.51 0.489 2.2
SAFE1  0.05  0.05 -0.06 -0.01  0.11  0.08  0.64  0.10 0.62 0.380 1.2
SAFE2  0.01  0.06 -0.02 -0.04  0.06  0.11  0.67  0.06 0.61 0.394 1.1
SAFE3  0.04  0.00  0.03 -0.04  0.01  0.06  0.59  0.14 0.50 0.497 1.2
SAFE4 -0.01  0.03  0.06  0.03 -0.03 -0.01  0.85  0.02 0.78 0.221 1.0
SAFE5  0.06  0.04  0.01  0.11  0.03 -0.06  0.76 -0.03 0.66 0.340 1.1
SOCI1  0.14  0.13  0.12 -0.13  0.06  0.46  0.02  0.04 0.44 0.561 1.8
TECH1  0.15  0.05  0.11 -0.01  0.52  0.09  0.11  0.00 0.61 0.389 1.5
TECH2  0.06  0.00  0.01  0.01  0.75  0.06  0.04  0.02 0.70 0.299 1.0
TECH3 -0.02 -0.04 -0.04  0.02  0.95  0.00 -0.01  0.04 0.86 0.136 1.0
TECH4 -0.02  0.03  0.08 -0.01  0.89 -0.02 -0.04 -0.01 0.85 0.152 1.0
TECH5  0.00  0.01  0.06  0.04  0.67  0.02  0.12 -0.03 0.61 0.389 1.1
UNRE1  0.00 -0.01  0.71  0.06  0.07  0.02 -0.04  0.10 0.68 0.323 1.1
UNRE2 -0.04  0.05  0.87  0.07 -0.01 -0.03 -0.05  0.04 0.78 0.221 1.0
UNRE3  0.09  0.00  0.85 -0.09  0.05  0.02 -0.01 -0.03 0.75 0.249 1.1
UNRE4  0.00  0.00  0.85  0.02  0.04  0.00  0.07 -0.03 0.82 0.179 1.0
UNRE5 -0.07 -0.05  0.64  0.05  0.01  0.18  0.07  0.00 0.61 0.392 1.2

                       ML2  ML3  ML8  ML5  ML1  ML6  ML4  ML7
SS loadings           4.43 3.77 3.74 3.63 3.59 3.25 3.19 3.18
Proportion Var        0.11 0.09 0.09 0.09 0.09 0.08 0.08 0.08
Cumulative Var        0.11 0.20 0.29 0.38 0.47 0.55 0.62 0.70
Proportion Explained  0.15 0.13 0.13 0.13 0.12 0.11 0.11 0.11
Cumulative Proportion 0.15 0.28 0.41 0.54 0.67 0.78 0.89 1.00

 With factor correlations of
     ML2  ML3  ML8  ML5  ML1  ML6  ML4  ML7
ML2 1.00 0.22 0.22 0.30 0.33 0.45 0.26 0.37
ML3 0.22 1.00 0.24 0.23 0.22 0.32 0.37 0.34
ML8 0.22 0.24 1.00 0.43 0.68 0.54 0.30 0.29
ML5 0.30 0.23 0.43 1.00 0.52 0.51 0.28 0.26
ML1 0.33 0.22 0.68 0.52 1.00 0.49 0.30 0.30
ML6 0.45 0.32 0.54 0.51 0.49 1.00 0.29 0.28
ML4 0.26 0.37 0.30 0.28 0.30 0.29 1.00 0.50
```

```
ML7 0.37 0.34 0.29 0.26 0.30 0.28 0.50 1.00

Mean item complexity =  1.2
Test of the hypothesis that 8 factors are sufficient.

df null model =  820  with the objective function =  37.43 with Chi Square =  20383.22
df of  the model are 520  and the objective function was  3.41

The root mean square of the residuals (RMSR) is  0.02
The df corrected root mean square of the residuals is  0.03

The harmonic n.obs is  560 with the empirical chi square  432.54  with prob <  1
The total n.obs was  560  with Likelihood Chi Square =  1836.75  with prob <  3.6e-146

Tucker Lewis Index of factoring reliability =  0.893
RMSEA index =  0.067  and the 90 % confidence intervals are  0.064 0.071
BIC =  -1453.77
Fit based upon off diagonal values = 1
Measures of factor score adequacy
                                                   ML2  ML3  ML8  ML5  ML1  ML6
Correlation of (regression) scores with factors   0.98 0.98 0.97 0.96 0.97 0.96
Multiple R square of scores with factors          0.96 0.95 0.94 0.92 0.94 0.92
Minimum correlation of possible factor scores     0.92 0.91 0.87 0.85 0.89 0.84
                                                   ML4  ML7
Correlation of (regression) scores with factors   0.95 0.95
Multiple R square of scores with factors          0.90 0.90
Minimum correlation of possible factor scores     0.80 0.80
```

```
final8withoutSOCI1<- c("SOCI2", "SOCI3", "USEF1", "USEF2", "USEF5", "USEF4", "USEF3", "SOC
final8_SOCI1 <- nor[ ,!(names(nor) %in% final8withoutSOCI1)]
final8f6 = fa(final8_SOCI1, nfactors=8, rotate="oblimin", fm="ml")
final8f6
```

```
Factor Analysis using method =  ml
Call: fa(r = final8_SOCI1, nfactors = 8, rotate = "oblimin", fm = "ml")
Standardized loadings (pattern matrix) based upon correlation matrix
        ML2   ML3   ML8   ML5   ML1   ML4   ML7   ML6   h2    u2 com
COMP1 -0.02  0.05  0.04  0.77  0.04  0.04 -0.07  0.03 0.68 0.316 1.0
COMP2 -0.03  0.00  0.02  0.80  0.06  0.08 -0.04 -0.01 0.72 0.283 1.0
COMP3  0.00  0.06  0.04  0.79  0.01  0.02 -0.05  0.05 0.73 0.272 1.0
COMP4  0.08 -0.06  0.02  0.74 -0.03 -0.04  0.14  0.11 0.73 0.272 1.2
```

COMP5	0.05	-0.01	-0.02	0.84	0.01	-0.02	0.09	-0.03	0.74	0.256	1.0
CONF1	0.72	0.04	0.07	0.13	0.01	0.03	-0.02	0.04	0.67	0.325	1.1
CONF2	0.93	-0.02	0.03	0.00	0.00	0.01	-0.03	-0.02	0.85	0.152	1.0
CONF3	0.93	-0.03	-0.01	0.00	-0.01	-0.01	0.05	-0.03	0.85	0.150	1.0
CONF4	0.92	0.02	0.01	0.00	-0.01	0.00	0.00	0.01	0.86	0.138	1.0
CONF5	0.89	0.02	-0.04	-0.02	0.04	0.02	0.00	0.04	0.84	0.155	1.0
INSE1	0.10	0.52	0.07	0.12	0.12	-0.02	-0.02	0.10	0.52	0.476	1.4
INSE2	0.00	0.94	0.00	0.02	-0.02	-0.02	0.03	0.01	0.90	0.097	1.0
INSE3	0.01	0.86	-0.07	0.04	0.07	0.00	-0.01	0.04	0.78	0.225	1.0
INSE4	-0.03	0.88	0.04	-0.05	-0.03	0.03	0.00	-0.05	0.76	0.242	1.0
INSE5	-0.01	0.88	0.02	-0.02	-0.04	0.05	0.01	-0.01	0.79	0.210	1.0
PRIV1	0.09	0.12	-0.03	0.10	0.11	-0.11	0.68	0.02	0.63	0.368	1.3
PRIV2	0.06	0.06	0.01	0.04	0.07	-0.10	0.79	0.01	0.69	0.306	1.1
PRIV3	-0.03	-0.01	0.02	0.01	-0.02	0.26	0.72	-0.06	0.71	0.288	1.3
PRIV4	-0.01	0.02	0.11	-0.02	-0.03	0.06	0.76	0.02	0.68	0.319	1.1
PRIV5	-0.03	-0.04	-0.01	0.00	0.01	0.19	0.66	0.08	0.61	0.394	1.2
OVER1	0.01	-0.04	0.02	0.07	0.05	0.01	-0.04	0.84	0.81	0.188	1.0
OVER2	-0.07	0.04	0.04	0.07	0.03	0.00	0.00	0.86	0.85	0.152	1.0
OVER3	0.17	0.06	0.07	-0.16	-0.05	0.02	0.13	0.57	0.47	0.535	1.6
OVER4	0.15	0.09	-0.01	-0.04	0.05	0.03	0.03	0.58	0.51	0.490	1.2
OVER5	0.19	0.01	0.09	0.18	-0.07	0.11	0.06	0.40	0.51	0.493	2.3
SAFE1	0.06	0.05	-0.05	-0.01	0.11	0.64	0.10	0.07	0.62	0.381	1.2
SAFE2	0.02	0.06	-0.02	-0.04	0.06	0.67	0.06	0.10	0.60	0.395	1.1
SAFE3	0.04	0.00	0.04	-0.03	0.01	0.59	0.14	0.06	0.50	0.498	1.2
SAFE4	-0.01	0.02	0.06	0.03	-0.03	0.85	0.02	-0.01	0.78	0.220	1.0
SAFE5	0.06	0.04	0.00	0.11	0.03	0.76	-0.03	-0.06	0.66	0.340	1.1
TECH1	0.16	0.05	0.11	-0.01	0.52	0.11	0.00	0.09	0.61	0.389	1.5
TECH2	0.06	0.00	0.01	0.01	0.75	0.04	0.02	0.06	0.70	0.299	1.0
TECH3	-0.02	-0.04	-0.04	0.02	0.95	-0.01	0.04	0.00	0.86	0.136	1.0
TECH4	-0.02	0.03	0.08	-0.01	0.89	-0.04	-0.01	-0.02	0.85	0.152	1.0
TECH5	0.00	0.01	0.06	0.04	0.67	0.12	-0.02	0.02	0.61	0.389	1.1
UNRE1	0.00	-0.01	0.71	0.06	0.06	-0.04	0.10	0.02	0.68	0.323	1.1
UNRE2	-0.04	0.05	0.87	0.07	-0.01	-0.05	0.04	-0.03	0.78	0.220	1.0
UNRE3	0.09	0.00	0.85	-0.09	0.04	-0.01	-0.03	0.01	0.75	0.250	1.1
UNRE4	0.00	0.00	0.85	0.02	0.04	0.07	-0.03	0.00	0.82	0.180	1.0
UNRE5	-0.06	-0.04	0.64	0.05	0.01	0.07	0.01	0.18	0.61	0.391	1.2

	ML2	ML3	ML8	ML5	ML1	ML4	ML7	ML6
SS loadings	4.40	3.73	3.70	3.66	3.55	3.17	3.17	2.95
Proportion Var	0.11	0.09	0.09	0.09	0.09	0.08	0.08	0.07
Cumulative Var	0.11	0.20	0.30	0.39	0.48	0.56	0.63	0.71
Proportion Explained	0.16	0.13	0.13	0.13	0.13	0.11	0.11	0.10
Cumulative Proportion	0.16	0.29	0.42	0.55	0.67	0.78	0.90	1.00

```
 With factor correlations of 
     ML2  ML3  ML8  ML5  ML1  ML4  ML7  ML6
ML2 1.00 0.22 0.22 0.31 0.33 0.26 0.37 0.44
ML3 0.22 1.00 0.24 0.24 0.22 0.37 0.34 0.31
ML8 0.22 0.24 1.00 0.44 0.68 0.30 0.29 0.54
ML5 0.31 0.24 0.44 1.00 0.52 0.28 0.27 0.53
ML1 0.33 0.22 0.68 0.52 1.00 0.30 0.30 0.50
ML4 0.26 0.37 0.30 0.28 0.30 1.00 0.50 0.29
ML7 0.37 0.34 0.29 0.27 0.30 0.50 1.00 0.28
ML6 0.44 0.31 0.54 0.53 0.50 0.29 0.28 1.00

Mean item complexity =  1.1
Test of the hypothesis that 8 factors are sufficient.

df null model =  780  with the objective function =  36.81 with Chi Square =  20056.48
df of  the model are 488  and the objective function was  3.32 

The root mean square of the residuals (RMSR) is  0.02 
The df corrected root mean square of the residuals is  0.03 

The harmonic n.obs is  560 with the empirical chi square  423.5  with prob <  0.98 
The total n.obs was  560  with Likelihood Chi Square =  1791.46  with prob <  5.5e-148 

Tucker Lewis Index of factoring reliability =  0.891
RMSEA index =  0.069  and the 90 % confidence intervals are  0.066 0.073
BIC =  -1296.57
Fit based upon off diagonal values = 1
Measures of factor score adequacy             
                                                   ML2  ML3  ML8  ML5  ML1  ML4
Correlation of (regression) scores with factors   0.98 0.98 0.97 0.96 0.97 0.95
Multiple R square of scores with factors          0.96 0.95 0.94 0.92 0.94 0.90
Minimum correlation of possible factor scores     0.92 0.91 0.87 0.85 0.89 0.80
                                                   ML7  ML6
Correlation of (regression) scores with factors   0.95 0.96
Multiple R square of scores with factors          0.90 0.92
Minimum correlation of possible factor scores     0.80 0.84
```

```
final8withoutOVER5<- c("SOCI2", "SOCI3", "USEF1", "USEF2", "USEF5", "USEF4", "USEF3", "SOC
final8_OVER5 <- nor[ ,!(names(nor) %in% final8withoutOVER5)]
final8f7 = fa(final8_OVER5, nfactors=8, rotate="oblimin", fm="ml")
final8f7
```

Factor Analysis using method = ml

Call: fa(r = final8_OVER5, nfactors = 8, rotate = "oblimin", fm = "ml")

Standardized loadings (pattern matrix) based upon correlation matrix

	ML2	ML3	ML8	ML5	ML1	ML7	ML4	ML6	h2	u2	com
COMP1	-0.02	0.04	0.04	0.78	0.04	-0.07	0.04	0.03	0.69	0.311	1.0
COMP2	-0.03	0.00	0.02	0.80	0.05	-0.04	0.08	0.00	0.72	0.278	1.0
COMP3	0.00	0.06	0.04	0.79	0.01	-0.05	0.02	0.05	0.73	0.270	1.0
COMP4	0.08	-0.06	0.02	0.73	-0.03	0.15	-0.04	0.11	0.72	0.280	1.2
COMP5	0.05	-0.01	-0.02	0.84	0.01	0.09	-0.02	-0.03	0.74	0.262	1.0
CONF1	0.72	0.04	0.07	0.13	0.01	-0.02	0.03	0.04	0.67	0.325	1.1
CONF2	0.93	-0.02	0.03	0.00	0.00	-0.03	0.01	-0.01	0.85	0.152	1.0
CONF3	0.93	-0.03	-0.01	0.00	-0.01	0.05	-0.01	-0.04	0.85	0.152	1.0
CONF4	0.92	0.01	0.01	0.00	-0.01	0.00	0.00	0.01	0.86	0.136	1.0
CONF5	0.89	0.02	-0.04	-0.02	0.04	0.00	0.02	0.04	0.85	0.154	1.0
INSE1	0.10	0.52	0.07	0.12	0.11	-0.02	-0.01	0.10	0.52	0.476	1.4
INSE2	0.00	0.94	0.00	0.02	-0.02	0.03	-0.02	0.01	0.90	0.097	1.0
INSE3	0.01	0.85	-0.07	0.04	0.07	-0.01	0.00	0.04	0.78	0.225	1.0
INSE4	-0.03	0.88	0.04	-0.05	-0.02	0.00	0.03	-0.05	0.76	0.242	1.0
INSE5	-0.01	0.88	0.02	-0.02	-0.04	0.01	0.05	-0.01	0.79	0.210	1.0
PRIV1	0.10	0.12	-0.03	0.10	0.11	0.68	-0.11	0.02	0.63	0.370	1.3
PRIV2	0.06	0.06	0.01	0.04	0.07	0.78	-0.10	0.01	0.69	0.308	1.1
PRIV3	-0.03	-0.01	0.02	0.01	-0.02	0.72	0.25	-0.05	0.71	0.286	1.3
PRIV4	-0.01	0.02	0.11	-0.02	-0.03	0.77	0.06	0.03	0.68	0.317	1.1
PRIV5	-0.02	-0.04	-0.01	0.00	0.01	0.67	0.19	0.08	0.61	0.393	1.2
OVER1	0.02	-0.04	0.02	0.07	0.04	-0.04	0.01	0.85	0.82	0.181	1.0
OVER2	-0.06	0.04	0.03	0.07	0.02	0.01	0.01	0.87	0.86	0.139	1.0
OVER3	0.18	0.06	0.07	-0.16	-0.05	0.14	0.01	0.54	0.45	0.551	1.7
OVER4	0.16	0.10	-0.01	-0.04	0.05	0.03	0.02	0.55	0.49	0.511	1.3
SAFE1	0.06	0.05	-0.05	0.00	0.11	0.10	0.63	0.06	0.61	0.386	1.2
SAFE2	0.02	0.06	-0.01	-0.04	0.06	0.06	0.67	0.10	0.60	0.399	1.1
SAFE3	0.04	0.01	0.04	-0.03	0.01	0.14	0.58	0.05	0.50	0.501	1.2
SAFE4	-0.01	0.02	0.06	0.03	-0.03	0.02	0.85	0.00	0.78	0.216	1.0
SAFE5	0.06	0.04	0.00	0.11	0.03	-0.03	0.77	-0.05	0.66	0.337	1.1
TECH1	0.16	0.05	0.11	-0.01	0.52	0.00	0.11	0.09	0.61	0.389	1.5
TECH2	0.06	0.01	0.01	0.01	0.75	0.02	0.04	0.06	0.70	0.298	1.0
TECH3	-0.02	-0.04	-0.04	0.02	0.95	0.04	-0.01	0.00	0.86	0.136	1.0
TECH4	-0.03	0.03	0.08	-0.01	0.90	-0.01	-0.04	-0.02	0.85	0.152	1.0
TECH5	0.00	0.01	0.06	0.04	0.67	-0.03	0.12	0.02	0.61	0.390	1.1
UNRE1	0.00	-0.01	0.71	0.06	0.06	0.10	-0.04	0.02	0.68	0.324	1.1
UNRE2	-0.04	0.05	0.87	0.07	-0.01	0.04	-0.04	-0.02	0.78	0.220	1.0
UNRE3	0.09	0.00	0.85	-0.08	0.05	-0.03	-0.01	0.01	0.75	0.251	1.1
UNRE4	0.00	0.00	0.85	0.02	0.04	-0.03	0.07	0.00	0.82	0.179	1.0
UNRE5	-0.06	-0.04	0.64	0.05	0.00	0.01	0.07	0.18	0.61	0.391	1.2

```
                     ML2  ML3  ML8  ML5  ML1  ML7  ML4  ML6
SS loadings          4.34 3.72 3.65 3.58 3.56 3.16 3.12 2.68
Proportion Var       0.11 0.10 0.09 0.09 0.09 0.08 0.08 0.07
Cumulative Var       0.11 0.21 0.30 0.39 0.48 0.56 0.64 0.71
Proportion Explained 0.16 0.13 0.13 0.13 0.13 0.11 0.11 0.10
Cumulative Proportion 0.16 0.29 0.42 0.55 0.68 0.79 0.90 1.00

 With factor correlations of
     ML2  ML3  ML8  ML5  ML1  ML7  ML4  ML6
ML2 1.00 0.22 0.22 0.31 0.33 0.37 0.26 0.43
ML3 0.22 1.00 0.24 0.24 0.22 0.34 0.37 0.31
ML8 0.22 0.24 1.00 0.44 0.68 0.29 0.29 0.55
ML5 0.31 0.24 0.44 1.00 0.53 0.27 0.28 0.53
ML1 0.33 0.22 0.68 0.53 1.00 0.31 0.30 0.51
ML7 0.37 0.34 0.29 0.27 0.31 1.00 0.50 0.27
ML4 0.26 0.37 0.29 0.28 0.30 0.50 1.00 0.28
ML6 0.43 0.31 0.55 0.53 0.51 0.27 0.28 1.00

Mean item complexity =  1.1
Test of the hypothesis that 8 factors are sufficient.

df null model =  741  with the objective function =  35.95 with Chi Square =  19600.64
df of  the model are 457  and the objective function was  3.13

The root mean square of the residuals (RMSR) is  0.02
The df corrected root mean square of the residuals is  0.03

The harmonic n.obs is  560 with the empirical chi square  381.47  with prob <  1
The total n.obs was  560  with Likelihood Chi Square =  1688.57  with prob <  1.8e-140

Tucker Lewis Index of factoring reliability =  0.893
RMSEA index =  0.069  and the 90 % confidence intervals are  0.066 0.073
BIC =  -1203.3
Fit based upon off diagonal values = 1
Measures of factor score adequacy
                                                   ML2  ML3  ML8  ML5  ML1  ML7
Correlation of (regression) scores with factors   0.98 0.98 0.97 0.96 0.97 0.95
Multiple R square of scores with factors          0.96 0.95 0.93 0.92 0.94 0.90
Minimum correlation of possible factor scores     0.92 0.91 0.87 0.85 0.89 0.80
                                                   ML4  ML6
Correlation of (regression) scores with factors   0.95 0.96
Multiple R square of scores with factors          0.90 0.92
```

```
Minimum correlation of possible factor scores     0.80 0.84
```

```
#final solution
final8withoutINSE1<- c("SOCI2", "SOCI3", "USEF1", "USEF2", "USEF5", "USEF4", "USEF3", "SOC
final8_INSE1 <- nor[ ,!(names(nor) %in% final8withoutINSE1)]
final8f8 = fa(final8_INSE1, nfactors=8, rotate="oblimin", fm="ml")
final8f8
```

```
Factor Analysis using method =  ml
Call: fa(r = final8_INSE1, nfactors = 8, rotate = "oblimin", fm = "ml")
Standardized loadings (pattern matrix) based upon correlation matrix
        ML2   ML8   ML5   ML1   ML3   ML4   ML7   ML6   h2   u2 com
COMP1 -0.02  0.04  0.78  0.04  0.05 -0.07  0.03  0.03 0.69 0.31 1.0
COMP2 -0.03  0.02  0.80  0.05  0.00 -0.04  0.08  0.00 0.72 0.28 1.0
COMP3  0.00  0.04  0.79  0.01  0.06 -0.05  0.02  0.05 0.73 0.27 1.0
COMP4  0.08  0.02  0.73 -0.03 -0.06  0.14 -0.04  0.11 0.72 0.28 1.2
COMP5  0.05 -0.02  0.84  0.01 -0.01  0.09 -0.02 -0.03 0.74 0.26 1.0
CONF1  0.72  0.07  0.13  0.01  0.04 -0.02  0.03  0.04 0.67 0.33 1.1
CONF2  0.93  0.03  0.00  0.00 -0.02 -0.03  0.01 -0.01 0.85 0.15 1.0
CONF3  0.93 -0.01  0.00 -0.01 -0.03  0.05 -0.01 -0.04 0.85 0.15 1.0
CONF4  0.92  0.01  0.01 -0.01  0.01  0.00  0.00  0.01 0.86 0.14 1.0
CONF5  0.89 -0.04 -0.02  0.04  0.02  0.00  0.02  0.04 0.85 0.15 1.0
INSE2  0.01  0.00  0.03 -0.02  0.93  0.03 -0.02  0.02 0.90 0.10 1.0
INSE3  0.02 -0.06  0.04  0.07  0.85 -0.01  0.00  0.05 0.77 0.23 1.0
INSE4 -0.03  0.04 -0.05 -0.02  0.89  0.00  0.03 -0.05 0.77 0.23 1.0
INSE5  0.00  0.02 -0.01 -0.03  0.88  0.01  0.04 -0.01 0.80 0.20 1.0
PRIV1  0.10 -0.03  0.10  0.11  0.11  0.68 -0.11  0.02 0.63 0.37 1.3
PRIV2  0.06  0.01  0.04  0.07  0.06  0.78 -0.10  0.01 0.69 0.31 1.1
PRIV3 -0.03  0.02  0.01 -0.02 -0.01  0.72  0.25 -0.05 0.71 0.29 1.3
PRIV4 -0.01  0.11 -0.02 -0.03  0.02  0.77  0.06  0.03 0.68 0.32 1.1
PRIV5 -0.02 -0.01  0.00  0.01 -0.04  0.67  0.19  0.08 0.61 0.39 1.2
OVER1  0.02  0.02  0.07  0.04 -0.04 -0.04  0.01  0.84 0.82 0.18 1.0
OVER2 -0.06  0.03  0.07  0.02  0.04  0.01  0.00  0.87 0.86 0.14 1.0
OVER3  0.19  0.07 -0.16 -0.05  0.06  0.13  0.01  0.54 0.45 0.55 1.7
OVER4  0.16 -0.01 -0.04  0.06  0.10  0.03  0.02  0.55 0.49 0.51 1.3
SAFE1  0.06 -0.05  0.00  0.11  0.05  0.10  0.63  0.06 0.61 0.39 1.2
SAFE2  0.02 -0.01 -0.04  0.06  0.06  0.06  0.67  0.10 0.60 0.40 1.1
SAFE3  0.04  0.04 -0.03  0.01  0.01  0.14  0.58  0.05 0.50 0.50 1.2
SAFE4 -0.01  0.06  0.03 -0.03  0.02  0.02  0.85  0.00 0.79 0.21 1.0
SAFE5  0.06  0.00  0.10  0.03  0.04 -0.03  0.77 -0.05 0.66 0.34 1.1
TECH1  0.16  0.11 -0.01  0.52  0.05 -0.01  0.11  0.09 0.61 0.39 1.5
TECH2  0.06  0.01  0.01  0.75  0.01  0.02  0.04  0.06 0.70 0.30 1.0
```

```
TECH3 -0.02 -0.04  0.02  0.96 -0.04  0.03 -0.01  0.00 0.87 0.13 1.0
TECH4 -0.02  0.08 -0.01  0.89  0.03 -0.01 -0.04 -0.02 0.85 0.15 1.0
TECH5  0.00  0.06  0.04  0.67  0.01 -0.03  0.12  0.03 0.61 0.39 1.1
UNRE1  0.00  0.71  0.06  0.06 -0.02  0.10 -0.04  0.02 0.68 0.32 1.1
UNRE2 -0.04  0.87  0.07 -0.01  0.05  0.04 -0.04 -0.02 0.78 0.22 1.0
UNRE3  0.09  0.85 -0.08  0.05  0.00 -0.03 -0.01  0.01 0.75 0.25 1.1
UNRE4  0.00  0.85  0.02  0.04  0.00 -0.03  0.07  0.00 0.82 0.18 1.0
UNRE5 -0.06  0.64  0.05  0.00 -0.04  0.01  0.07  0.18 0.61 0.39 1.2

                       ML2  ML8  ML5  ML1  ML3  ML4  ML7  ML6
SS loadings           4.31 3.62 3.54 3.53 3.39 3.16 3.12 2.63
Proportion Var        0.11 0.10 0.09 0.09 0.09 0.08 0.08 0.07
Cumulative Var        0.11 0.21 0.30 0.39 0.48 0.57 0.65 0.72
Proportion Explained  0.16 0.13 0.13 0.13 0.12 0.12 0.11 0.10
Cumulative Proportion 0.16 0.29 0.42 0.55 0.67 0.79 0.90 1.00

 With factor correlations of
     ML2  ML8  ML5  ML1  ML3  ML4  ML7  ML6
ML2 1.00 0.22 0.31 0.33 0.21 0.37 0.26 0.43
ML8 0.22 1.00 0.44 0.68 0.23 0.29 0.29 0.55
ML5 0.31 0.44 1.00 0.53 0.23 0.27 0.28 0.53
ML1 0.33 0.68 0.53 1.00 0.21 0.31 0.31 0.50
ML3 0.21 0.23 0.23 0.21 1.00 0.34 0.37 0.30
ML4 0.37 0.29 0.27 0.31 0.34 1.00 0.50 0.28
ML7 0.26 0.29 0.28 0.31 0.37 0.50 1.00 0.29
ML6 0.43 0.55 0.53 0.50 0.30 0.28 0.29 1.00

Mean item complexity =  1.1
Test of the hypothesis that 8 factors are sufficient.

df null model =  703  with the objective function =  35.14 with Chi Square =  19167.86
df of  the model are 427  and the objective function was  3.02

The root mean square of the residuals (RMSR) is  0.02
The df corrected root mean square of the residuals is  0.03

The harmonic n.obs is  560 with the empirical chi square  363.37  with prob <  0.99
The total n.obs was  560  with Likelihood Chi Square =  1630.98  with prob <  6.4e-140

Tucker Lewis Index of factoring reliability =  0.892
RMSEA index =  0.071  and the 90 % confidence intervals are  0.067 0.075
BIC =  -1071.05
Fit based upon off diagonal values = 1
```

```
Measures of factor score adequacy
                                                   ML2  ML8  ML5  ML1  ML3  ML4
Correlation of (regression) scores with factors   0.98 0.97 0.96 0.97 0.97 0.95
Multiple R square of scores with factors          0.96 0.93 0.92 0.94 0.95 0.90
Minimum correlation of possible factor scores     0.92 0.87 0.85 0.89 0.90 0.80
                                                   ML7  ML6
Correlation of (regression) scores with factors   0.95 0.96
Multiple R square of scores with factors          0.90 0.92
Minimum correlation of possible factor scores     0.80 0.84
```

```
#final solution
final8withoutTECH1<- c("SOCI2", "SOCI3", "USEF1", "USEF2", "USEF5", "USEF4", "USEF3", "SOC
final8_TECH1 <- nor[ ,!(names(nor) %in% final8withoutTECH1)]
final8f8a = fa(final8_TECH1, nfactors=8, rotate="oblimin", fm="ml")
final8f8a
```

```
Factor Analysis using method =  ml
Call: fa(r = final8_TECH1, nfactors = 8, rotate = "oblimin", fm = "ml")
Standardized loadings (pattern matrix) based upon correlation matrix
        ML2   ML8   ML5   ML3   ML7   ML1   ML4   ML6   h2   u2 com
COMP1 -0.02  0.04  0.78  0.05 -0.07  0.04  0.03  0.03 0.69 0.31 1.0
COMP2 -0.03  0.02  0.80  0.00 -0.04  0.05  0.08  0.00 0.72 0.28 1.0
COMP3  0.00  0.04  0.80  0.06 -0.05  0.00  0.02  0.05 0.73 0.27 1.0
COMP4  0.08  0.02  0.73 -0.06  0.14 -0.03 -0.04  0.11 0.72 0.28 1.2
COMP5  0.05 -0.02  0.84 -0.01  0.09  0.01 -0.02 -0.03 0.74 0.26 1.0
CONF1  0.72  0.07  0.13  0.04 -0.02  0.01  0.03  0.04 0.68 0.32 1.1
CONF2  0.93  0.03  0.00 -0.02 -0.03 -0.01  0.01 -0.01 0.85 0.15 1.0
CONF3  0.93 -0.01  0.00 -0.03  0.05 -0.01 -0.01 -0.04 0.85 0.15 1.0
CONF4  0.92  0.01  0.00  0.01  0.00 -0.01  0.01  0.01 0.86 0.14 1.0
CONF5  0.89 -0.04 -0.02  0.02  0.00  0.04  0.02  0.04 0.85 0.15 1.0
INSE2  0.01  0.00  0.03  0.93  0.03 -0.02 -0.02  0.02 0.90 0.10 1.0
INSE3  0.02 -0.06  0.04  0.84 -0.01  0.07  0.00  0.05 0.77 0.23 1.0
INSE4 -0.03  0.04 -0.05  0.89  0.00 -0.01  0.03 -0.05 0.77 0.23 1.0
INSE5  0.00  0.03 -0.01  0.88  0.01 -0.03  0.04 -0.01 0.80 0.20 1.0
PRIV1  0.09 -0.03  0.10  0.11  0.69  0.10 -0.11  0.02 0.63 0.37 1.2
PRIV2  0.06  0.01  0.05  0.06  0.79  0.07 -0.10  0.01 0.69 0.31 1.1
PRIV3 -0.03  0.02  0.01 -0.01  0.72 -0.02  0.26 -0.05 0.71 0.29 1.3
PRIV4 -0.01  0.11 -0.02  0.02  0.76 -0.03  0.06  0.03 0.68 0.32 1.1
PRIV5 -0.02 -0.01  0.00 -0.04  0.67  0.01  0.19  0.08 0.61 0.39 1.2
OVER1  0.02  0.02  0.07 -0.04 -0.04  0.04  0.01  0.84 0.82 0.18 1.0
OVER2 -0.06  0.03  0.06  0.04  0.01  0.02  0.01  0.87 0.86 0.14 1.0
OVER3  0.19  0.08 -0.15  0.06  0.14 -0.05  0.01  0.54 0.45 0.55 1.7
```

```
OVER4  0.17  0.00 -0.04  0.10  0.03  0.05  0.02  0.55 0.49 0.51 1.3
SAFE1  0.06 -0.05  0.00  0.05  0.11  0.11  0.63  0.06 0.61 0.39 1.2
SAFE2  0.03 -0.01 -0.04  0.06  0.06  0.06  0.66  0.10 0.60 0.40 1.1
SAFE3  0.04  0.04 -0.04  0.01  0.14  0.02  0.58  0.05 0.50 0.50 1.2
SAFE4 -0.01  0.06  0.03  0.02  0.02 -0.03  0.85  0.00 0.79 0.21 1.0
SAFE5  0.06  0.00  0.10  0.04 -0.03  0.03  0.77 -0.05 0.67 0.33 1.1
TECH2  0.07  0.02  0.02  0.01  0.02  0.73  0.04  0.06 0.69 0.31 1.0
TECH3 -0.01 -0.04  0.01 -0.04  0.03  0.95  0.00  0.00 0.87 0.13 1.0
TECH4 -0.01  0.08 -0.01  0.03 -0.01  0.90 -0.04 -0.02 0.85 0.15 1.0
TECH5  0.00  0.06  0.03  0.01 -0.03  0.67  0.12  0.03 0.61 0.39 1.1
UNRE1  0.00  0.71  0.06 -0.01  0.10  0.07 -0.03  0.02 0.68 0.32 1.1
UNRE2 -0.04  0.87  0.07  0.05  0.04 -0.01 -0.04 -0.02 0.78 0.22 1.0
UNRE3  0.09  0.85 -0.08  0.00 -0.03  0.04 -0.01  0.01 0.75 0.25 1.1
UNRE4  0.00  0.85  0.02  0.00 -0.03  0.04  0.07  0.00 0.82 0.18 1.0
UNRE5 -0.06  0.64  0.05 -0.04  0.01  0.00  0.07  0.18 0.61 0.39 1.2

                       ML2  ML8  ML5  ML3  ML7  ML1  ML4  ML6
SS loadings           4.26 3.57 3.54 3.37 3.16 3.12 3.08 2.59
Proportion Var        0.12 0.10 0.10 0.09 0.09 0.08 0.08 0.07
Cumulative Var        0.12 0.21 0.31 0.40 0.48 0.57 0.65 0.72
Proportion Explained  0.16 0.13 0.13 0.13 0.12 0.12 0.12 0.10
Cumulative Proportion 0.16 0.29 0.43 0.55 0.67 0.79 0.90 1.00

 With factor correlations of
     ML2  ML8  ML5  ML3  ML7  ML1  ML4  ML6
ML2 1.00 0.22 0.31 0.21 0.37 0.32 0.26 0.42
ML8 0.22 1.00 0.44 0.23 0.29 0.68 0.29 0.55
ML5 0.31 0.44 1.00 0.23 0.27 0.53 0.28 0.53
ML3 0.21 0.23 0.23 1.00 0.34 0.20 0.37 0.30
ML7 0.37 0.29 0.27 0.34 1.00 0.30 0.50 0.28
ML1 0.32 0.68 0.53 0.20 0.30 1.00 0.30 0.50
ML4 0.26 0.29 0.28 0.37 0.50 0.30 1.00 0.28
ML6 0.42 0.55 0.53 0.30 0.28 0.50 0.28 1.00

Mean item complexity =  1.1
Test of the hypothesis that 8 factors are sufficient.

df null model =  666  with the objective function =  34.1 with Chi Square =  18614.83
df of  the model are 398  and the objective function was  2.88

The root mean square of the residuals (RMSR) is  0.02
The df corrected root mean square of the residuals is  0.03
```

```
The harmonic n.obs is  560 with the empirical chi square  346.81  with prob <  0.97
The total n.obs was  560  with Likelihood Chi Square =  1556.07  with prob <  2.2e-136

Tucker Lewis Index of factoring reliability =  0.891
RMSEA index =  0.072  and the 90 % confidence intervals are  0.068 0.076
BIC =  -962.45
Fit based upon off diagonal values = 1
Measures of factor score adequacy
                                                   ML2  ML8  ML5  ML3  ML7  ML1
Correlation of (regression) scores with factors   0.98 0.97 0.96 0.97 0.95 0.97
Multiple R square of scores with factors          0.96 0.93 0.92 0.95 0.90 0.94
Minimum correlation of possible factor scores     0.92 0.87 0.85 0.90 0.80 0.89
                                                   ML4  ML6
Correlation of (regression) scores with factors   0.95 0.96
Multiple R square of scores with factors          0.90 0.92
Minimum correlation of possible factor scores     0.80 0.84
```
